# Long‐Term Changes in Survival of Eurasian Lynx in Three Reintroduced Populations in Switzerland

**DOI:** 10.1002/ece3.71095

**Published:** 2025-03-30

**Authors:** K. Vogt, F. Korner‐Nievergelt, S. Signer, F. Zimmermann, I. Marti, A. Ryser, A. Molinari‐Jobin, U. Breitenmoser, Ch. Breitenmoser‐Würsten

**Affiliations:** ^1^ Foundation KORA (Carnivore Ecology & Wildlife Management) Ittigen Switzerland; ^2^ Oikostat GmbH Ettiswil Switzerland; ^3^ Department of Environmental Sciences University of Basel Basel Switzerland; ^4^ Department of Ecology and Evolution University of Lausanne, Quartier UNIL‐Sorge, Bâtiment Biophore Lausanne Switzerland; ^5^ Institute for Fish and Wildlife Health, Vetsuisse Faculty University of Bern Bern Switzerland

**Keywords:** Eurasian lynx, *Lynx lynx*, mortality, reintroduction, survival, Switzerland

## Abstract

For conservation or management programs, basic data on vital rates are important but often hard to acquire for long‐lived and elusive wildlife species such as large carnivores. In this study, we analyzed long‐term changes in survival rates for different sexes and age classes (juvenile, subadult, adult) in three reintroduced Swiss lynx populations (Alps, Jura, Northeastern Switzerland). A novel modeling approach allowed us to combine picture data from camera trapping and lynx pictures resulting from chance observations, telemetry data, and dead recoveries over a monitoring period of 25 years (1997–2022). Mean annual survival of adult lynx varied between 0.71 and 0.81 for males and between 0.70 and 0.85 for females. Mean survival of subadults ranged between 0.59 and 0.89 among populations. Juvenile survival was highly variable and low on average (< 0.4). Our findings highlight that unknown sources of mortality exist in some populations and that future studies on mortality causes and potential effects of inbreeding on survival are needed to ensure long‐term conservation of the lynx in Switzerland. Our study can serve as a basis for future studies on population viability and conservation threats to the species in human‐dominated landscapes and demonstrates the complexity and high variation of survival between different age and sex classes in space and time, potentially leading to source‐sink dynamics.

## Graphical abstract

In this study, we analyzed the long‐term development of survival rates for different sexes and age classes in three reintroduced Swiss lynx populations. A novel modeling approach allowed us to combine picture data, telemetry data, and dead recoveries over a monitoring period of 25 years (1997–2022). Our findings highlight that unknown sources of mortality exist in some populations and that future studies on mortality causes and potential effects of inbreeding on survival are needed to ensure the long‐term conservation of the lynx in Switzerland. Copyright: KORA.

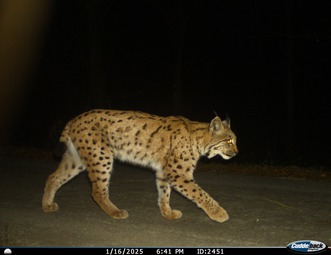

## 1 Introduction

By the beginning of the 20th century, most large carnivore species had been eradicated throughout Western Europe. Their successful recovery during the last decades has been the result of strict protection, the recovery of forests and prey species, the spread of autochthonous populations, and, in some cases, reintroductions (Breitenmoser 1998; Boitani and Linnell 2015; Linnell et al. 2009). Today, large carnivores in Europe live in multi‐use landscapes. Fragmentation of their habitat, traffic accidents, and human persecution are among the threats challenging their conservation (Litvaitis et al. 2015; Ripple et al. 2014). All of Europe's large carnivore species have proved to survive and reproduce successfully in multi‐use landscapes (Chapron et al. 2014). But while these landscapes provide an abundant prey base (e.g., high roe deer *Capreolus capreolus* densities), the associated high anthropogenic mortality may turn them into attractive sinks (Basille et al. 2009; Hansen 2011; Linnell et al. 2020). Therefore, conservation management of endangered carnivore species living in anthropogenic landscapes hinges upon understanding the factors influencing their survival and causes of mortality (Goodrich et al. 2008).

One of the species that has celebrated a comeback in several Western European countries is the Eurasian *lynx Lynx lynx* (hereafter lynx). Unlike other large carnivore species (e.g., wolves *Canis lupus* ), lynx are conservative colonizers (Zimmermann et al. 2005, 2007) and especially female lynx do not easily cross fragmented habitats (e.g., Herdtfelder et al. 2021). Due to their social system of female philopatry, lynx populations tend to grow at the edges, and long‐distance dispersals leading to the formation of new population nuclei far from the source population are rarely documented (Gajdárová et al. 2021; Port et al. 2020). Although this particular spatial organization was not yet well studied at that time, wildlife managers in the 1960's already had the insight that Western Europe would not be recolonized by lynx naturally. As a consequence, several reintroduction projects started from the 1970's resulting in successful establishment of lynx populations in Switzerland, France, Slovenia, the Czech Republic, and Germany (Linnell et al. 2009). 50 years after the first reintroductions, a narrow genetic base, slow population growth, and isolation of the reintroduced populations remain a challenge for lynx conservation in Western Europe (von von Arx et al. 2021). Known underlying causes are high anthropogenic mortality due to traffic collisions and, in some areas, illegal killings (Molinari‐Jobin et al. 2010; Arlettaz et al. 2021; von Arx et al. 2021), as well as loss of genetic diversity (Mueller et al. 2022). It is, therefore, important to monitor the demographic and genetic status of the reintroduced populations and their survival in Europe's multi‐use landscapes.

**FIGURE 1:**
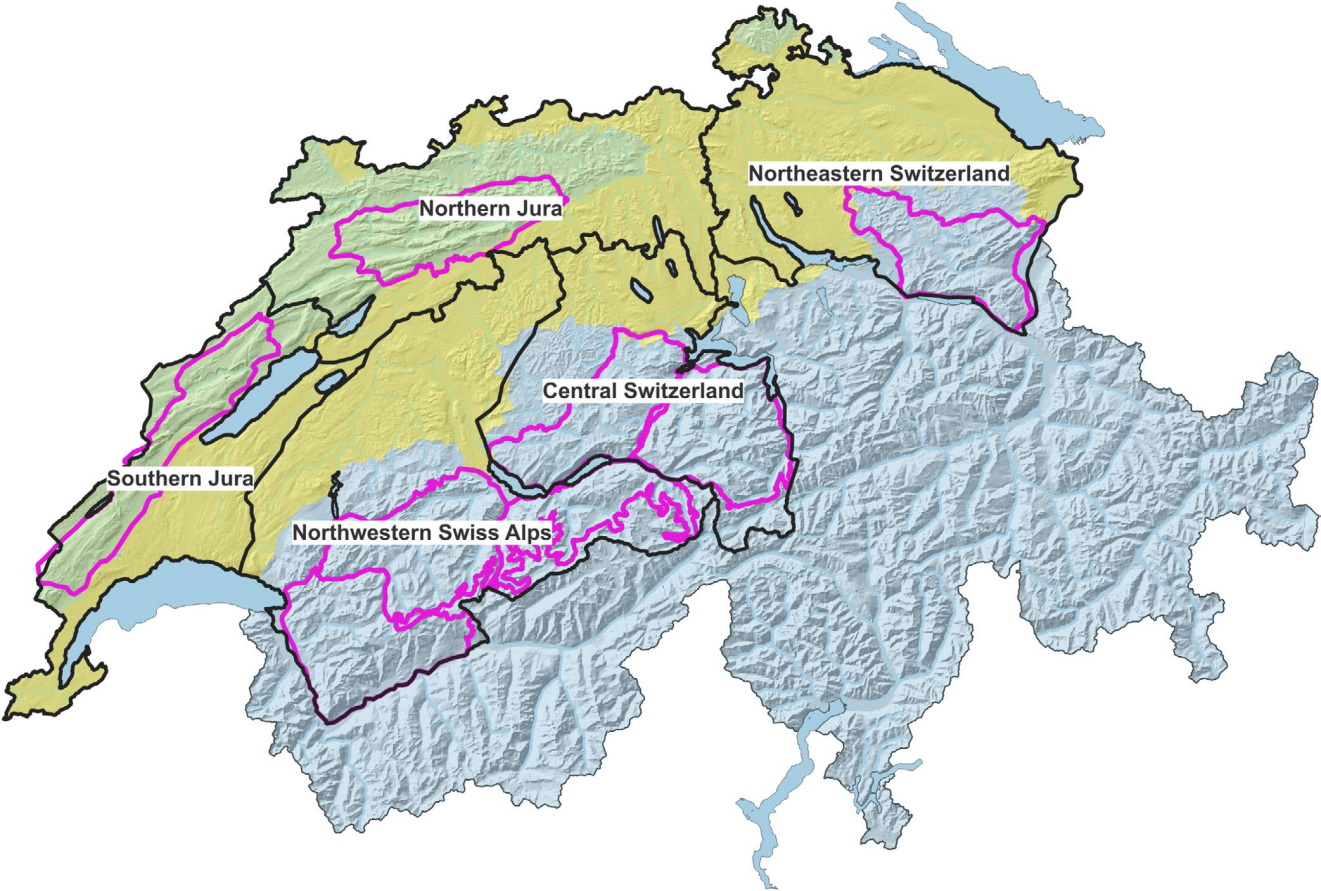
Geographic regions and study areas for lynx in Switzerland. Green shaded area = Jura Mountains, orange shaded area = Swiss Plateau, blue shaded area = Alps. Black lines show the subareas in which lynx survival was estimated during this study. Pink lines show reference areas where systematic camera‐trap monitoring for the lynx is conducted.

The lynx has been reintroduced to the Swiss Alps and the Jura Mountains (Figure 1) in the 1970's and 1980's. Between 2001 and 2008, another translocation of 12 individuals from the Swiss Jura and the Swiss Alps to Northeastern Switzerland (Figure 1) took place (Breitenmoser and Breitenmoser‐Würsten 2008). Lynx populations have subsequently developed in all three regions. For the first decades after the reintroductions, the Swiss populations grew slowly. Since the middle of the 1990's, increasing densities have been reported from some areas, while other areas showed an opposite trend (e.g., Breitenmoser‐Würsten et al. 2001; Kunz et al. 2021a, 2021b; Le Grand et al. 2022; Sterrer et al. 2022; Zimmermann et al. 2020). As of 2021, the Swiss Alps, including Northeastern Switzerland, held about 76% of the Swiss lynx population (~290 individuals), whereas the Swiss Jura Mountains are habitat to the remaining 24% (~70 individuals) (Foundation KORA 2024c). The lynx population in the entire Alpine arc is still considered endangered by IUCN Red List criteria, and much of the suitable habitat is still uncolonized (Molinari‐Jobin et al. 2020; von Arx 2020). Switzerland holds the core of the Alpine lynx population and has, therefore, a responsibility for the conservation of the species in this region. The Jura population, shared by Switzerland and France, is part of the Upper Rhine Lynx Metapopulation, and could serve as a source for the colonization of the southern Vosges mountains, the Black Forest, and the Schwäbische Alb (Drouet‐Hoguet et al. 2021).

In this study, we analyze long‐term changes in survival rates for different sexes and age classes and average survival time in the three reintroduced Swiss lynx populations. The aim of our study was to identify spatial and temporal variance in survival and compare our findings to other European populations. For conservation or management programs, basic data on vital rates are important (e.g., Andrén et al. 2020) but often hard to acquire for long‐lived and elusive wildlife species such as large carnivores (Brongo et al. 2005). We used an integrated modeling approach that allowed us to include all available data from the Swiss monitoring program spanning over 25 years (1997–2022). Particularly, we integrated picture data from systematic and opportunistic camera‐trap monitoring, lynx pictures resulting from chance observations, telemetry data, dead recoveries, and exact age of dead lynx into one combined statistical model within a Bayesian framework. This novel approach allowed us to (1) extend our analysis beyond the typically shorter study periods covered by survival studies based on intensive telemetry (Breitenmoser‐Würsten et al. 2007), and (2) include also areas of Switzerland where systematic camera‐trap monitoring was not conducted intensively enough to apply open population spatial capture–recapture models (Palmero et al. 2021). The integration of movement rates and age at death data further enabled us to estimate true survival (as opposed to apparent survival estimated by open population models; Palmero et al. 2021). Our study can serve as a basis for future studies on population viability and conservation threats to the species in human‐dominated landscapes. The approach can be applied to other long‐lived mammal species where aging of dead animals is feasible and records of individually identified animals are available.

## 2 Methods

### 2.1 Study areas

The Swiss Alps cover two‐thirds of Switzerland, with mountains up to 4634 m a.s.l. high. The Alpine region is characterized by a mixture of forest, open grassland, and rugged mountain terrain that is interspersed with settlements, including larger cities in the valleys and transportation networks (Swiss Federal Authorities 2023). The forested area is distributed along the valley sides, strongly fragmented by pastures and human settlements, and covers roughly 40% (KAWA 2015). Lynx were first reintroduced to Central Switzerland (Figure 1). In later years, a second population nucleus formed in the Northwestern Swiss Alps (Figure 1) which has today developed into the core area of the Alpine lynx population with the highest lynx densities recorded (Kunz et al. 2021a). The southeastern part of the Swiss Alps is only scarcely populated by lynx (Arlettaz et al. 2021; Foundation KORA 2024d) and was excluded from the analysis.

Northeastern Switzerland (Figure 1) geographically belongs to the Swiss Alps. However, the lynx population in this part of the country is genetically more similar to the Jura population (more founder animals from the Jura Mountains were successful; Foundation KORA 2024a). As of today, a small population of 22 (21–37, 95% CI) individuals has been estimated by camera‐trapping in the reference area (pink line, Figure 1; Sterrer et al. 2022). The population still remains rather isolated, and genetic admixture with the population in Central Switzerland has not yet been detected (Foundation KORA 2024a).

The Jura Mountains are shared by France and Switzerland, with the larger part lying in France. They cover about 10% of Switzerland, corresponding to approximately 4200 km^2^. Elevations range up to 1979 m a.s.l. About half of the landscape is covered by forests, followed by pastures and agricultural land, and only 8% of settlement area (Swiss Federal Authorities 2021a). About two‐thirds of the Jura lynx population resides in the French part of the mountain range (Von Arx et al. 2017; Molinari‐Jobin et al. 2020; Drouet‐Hoguet et al. 2021). The habitat is continuous, and there are no barriers between the two countries. In this study, however, we only refer to the Swiss part of the Jura population. The south of the Swiss Jura Mountains was colonized first by lynx, with a subsequent spread towards the north (Figure 1; Breitenmoser and Breitenmoser‐Würsten 2008).

The Jura Mountains and the Alps are separated by the densely populated Swiss Plateau, which harbors about two‐thirds of the country's inhabitants (Swiss Federal Authorities 2021b) and is fragmented by many motorways, railway lines, and settlements (Figure 1). This barrier has caused very limited genetic exchange between the Jura and the Alpine populations since the reintroduction (Breitenmoser‐Würsten and Obexer‐Ruff 2003), although lynx have started to colonize the Swiss Plateau in recent years (Foundation KORA 2020; Zimmermann and von Arx 2021).

### 2.2 Data collection

For estimating lynx survival, we combined all available data from 5 different sources: (1) lynx found dead, (2) lynx captured alive, (3) telemetry data, (4) data from systematic camera‐trap monitoring in reference areas, and (5) data from opportunistic camera‐trap monitoring (e.g., Zimmermann 2019) and pictures taken by chance (e.g., by hunters or nature photographers).

Since the reintroduction of the species to Switzerland in the 1970's and 1980's, all lynx found dead in Switzerland were reported to the responsible state authorities and were sent to the Institute for Fish and Wildlife Health (FIWI) for necropsy. Date of recovery, coordinates of the recovery site, sex, age class (juvenile, subadult, adult) and cause of death are documented for each individual. Pictures of both flanks are taken for each individual found dead and compared to the picture database maintained by KORA (Carnivore Ecology & Wildlife Management). For a subsample of dead individuals, the birth year was known (either from body size and dentition for juveniles and subadults, Marti and Ryser‐Degiorgis 2018, from previous camera trap monitoring records or determined by cementum annuli enumeration, Zapata et al. 1997).

Starting from the 1980's, lynx have been live‐captured in the frame of several scientific studies (e.g., Breitenmoser and Haller 1993; Breitenmoser‐Würsten et al. 2001; Breitenmoser‐Würsten et al. 2007; Jobin et al. 2000; Nagl et al. 2022; Vogt et al. 2016; Zimmermann et al. 2007) and translocation programmes (e.g., Ryser et al. 2006; Signer et al. 2021). Trapping methods include box traps, foot snares, and a remote‐controlled tele‐injection system (for details see: Ryser et al. 2005; Signer et al. 2021; Vogt et al. 2016). Most of the captured animals were equipped with VHF and/or (starting from 2005) with GPS collars (Wagener, Lotek, Vectronics; for details on models see: Nagl et al. 2022). The capture date, capture site coordinates, sex, and age category (according to body size and dentition; Marti and Ryser‐Degiorgis 2018) were documented for each individual, and pictures of both flanks were taken for subsequent identification in camera‐trap monitoring and comparison to the camera‐trap monitoring picture database maintained by KORA.

Starting from 1998, KORA has carried out regular systematic camera‐trap monitoring sessions within defined reference areas across the three populations (pink lines, Figure 1) in cooperation with the Swiss Federal Office for Environment and the responsible Cantonal hunting authorities. Sessions were conducted between late November and mid‐April for 60 consecutive days. Each reference area was monitored every 2–4 years, and lynx densities were estimated using non‐spatial capture‐recapture models (e.g., Zimmermann and Foresti 2016; Kunz et al. 2021a, 2021b; Sterrer et al. 2022; Sterrer et al. 2023a). However, not all reference areas were monitored since the beginning, and some changes in reference area sizes and delineations have occurred over the years (e.g., reference area in the Northwestern Swiss Alps; Foundation KORA 2021).

KORA also collects all lynx pictures taken during opportunistic monitoring conducted by the Cantonal hunting authorities or reported by hunters, naturalists, or the general public. All identifiable individuals from deterministic or opportunistic monitoring are entered in the picture database. For each individual, sex, reproduction, and birth year are recorded wherever possible. Sex can be assigned for females accompanied by cubs or from observation of the genital region (Zimmermann and Foresti 2016). Birth year can be assigned for individuals that were pictured for the first time as juveniles. The intensity of opportunistic monitoring has varied over time and between Cantons. We collected information on opportunistic monitoring effort from our own data (number of records received) and from expert opinions of the responsible hunting authorities for each Canton and each year (1997–2022). For each year, opportunistic monitoring effort was categorized as 1 (only passive collection of reported chance observations), 2 (Cantonal game wardens are equipped with camera traps but do not monitor at every possible occasion; or increased opportunistic monitoring effort in small part of territory), or 3 (intensive opportunistic camera‐trapping study or radio telemetry study has taken place in a substantial part of the territory; or game wardens set camera‐traps at every possible occasion; or management system in place that rewards hunters financially for reporting lynx pictures to Cantonal authorities; Von Arx et al. 2017).

### 2.3 Data preparation

#### 2.3.1 Alpine Population

For the Alpine lynx population, we included data from all identified individuals who had at least one record in the two subareas (Central Switzerland, Northwestern Swiss Alps; Figure 1). We included all data between 1 May 1997 and 30 April 2022 (Table 1).

**TABLE 1:** Number of individuals that were part of the final dataset for each study population. Assignment to subarea: Live individuals are assigned to the subarea where the majority of their detections took place. Dead individuals are assigned to the subarea where they have been found dead. The sum of the two subareas does not equal the total number of dead lynx because some individuals were found dead after emigration. Deleted = these individuals were not considered for the analysis.

	Pictured lynx			Lynx with radio‐collar^a^	Lynx found dead^a^	Dead lynx with known age^b^
	Both flanks	Right flank	Left flank			
Alpine population	459	81	70 (deleted)	82	64	229
Northwestern Swiss Alps	349	56		79	56	186
Central Switzerland	110	25		3	8	43
Northeastern Switzerland population	103	21 (deleted)	27	14	16	37
Jura population	310	70	70 (deleted)	24	151	152
Northern Jura	202	38		11	30	69
Southern Jura	146	32		13	31	83

^a^ All of these individuals were also pictured alive.

^b^ Includes also individuals who were not pictured alive before they were found dead.

To account for substantial trapping effort differences between months, data were grouped into capture occasions of 2‐month‐intervals within each “lynx year” (according to lynx reproductive cycle from 1st May to 30th of April; Molinari‐Jobin 2003). Two‐month‐intervals instead of one‐month‐intervals were chosen due to computational feasibility. Individuals can unambiguously be identified based on pictures only after they could be pictured from both sides and a left (L) and right (R) side could be assigned to the same individual. Unmatched left and right sides were kept as “L‐” and “R‐individuals” in the data. Therefore, a single individual could appear twice in the data, and if such an individual dies undetected, two apparent individuals disappear from the data. As a consequence, we would underestimate survival, if we kept all L‐ and R‐individuals in the dataset. However, deleting all unmatched L‐ and R‐individuals from the dataset would also introduce a bias, since lynx dying in their first year have a lower chance to have both sides matched than lynx who survive for several years. To reduce these biases, we deleted those single sided individuals which belonged to the less abundant group (in this case, 70 “L”‐individuals). A sensitivity analysis showed that deleting those individuals increased estimated juvenile survival by 3%–5% but had little effect on survival of the older age classes (see Supporting Information). Similarly, a non‐exhaustive simulation showed that deleting one of virtual “L” or “R” individuals produced essentially unbiased survival estimates (see Supporting Information). Consequently, our approach may only slightly overestimate survival. We further excluded orphaned juveniles that had been taken to a rehabilitation centre and later released as subadults. Data of individuals that were removed from the Alpine population in the frame of translocation projects were censored at the date of translocation. If the capture for translocation was their first known record, they were excluded (5 cases). The final data set was based on 11,445 pictures of 541 individuals of which 80 individuals were observed by means of telemetry (Table 1) yielding a total of 73,592 fixes.

When the birth year of an individual was known, we considered this individual as juvenile in its first year of life, subadult in its second year and adult from the third year of life onwards (≥ 2 years old). Lynx for which the birth year was not known were considered to be adults from their first detection. In this manner, we probably misclassified some subadults as adults. However, since most of these animals were individuals that appeared for the first time in an area during a systematic monitoring session and since systematic monitoring sessions were only conducted in winter/early spring every 2–3 years, we believe that our approach leads to less bias than considering these animals all as subadults or excluding them from the dataset. Animals with unknown sex (263 out of 541 individuals) were included in the dataset since excluding them would have led to an overestimation of survival (excluding all those individuals who did not live long enough to be sexed). Individuals with unknown sex were assigned a probability of belonging to one of the two sexes (see model description below). We considered individuals that had been detected outside the Alpine population only once and then moved back as if they had always been inside. To do so, we deleted the single record outside from the data (4 such cases). Including individual as a random factor in the model for detection probability accounts for temporary migration (see model description below).

Two hundred thirty‐one lynx with known age were found dead in the Alpine population during the study period. Of those, 43 were also included in the picture/telemetry datasets. For the analyses of the age‐at‐death data, all dead lynx found in the Alpine population for which the year of birth was known were included (*N* = 231), irrespective of whether they had been observed alive before.

#### 2.3.2 Northeastern Switzerland Population

For the Northeastern Switzerland lynx population, we included data from all identified individuals who had at least one record in the Northeastern Switzerland subarea (Figure 1). We included all data between 1 May 2001 and 30 April 2022 (Table 1). This also included data from the founder individuals of the population. To account for substantial trapping effort differences between months, data were grouped into capture occasions of 1‐month intervals within each “lynx year” (according to lynx reproductive cycle from 1st May to 30th of April). Shorter time intervals allow for more exact survival estimates. Since the dataset spanned a shorter time period and comprised only one subarea, model fitting was feasible (computing time) with a 1‐month resolution (as opposed to 2 months for the Alpine and Jura populations). Data were selected following the same procedure as for the Alpine population. Data of individuals that were removed from the Northeastern Switzerland population in the frame of translocation projects were censored at the date of translocation. The final data set contained 3335 pictures of 133 individuals, of which 14 individuals were observed by means of telemetry (Table 1) yielding a total of 7103 fixes.

Age, sex, and birth year were treated as for the Alpine population. We considered individuals that had been detected outside Northeastern Switzerland only once and then moved back into one of these areas as if they had always been inside (see description of multi‐state model). To do so, we deleted the single record outside from the data (12 such cases).

Thirty‐seven lynx with known ages were found dead in the Northeastern Switzerland population during the study period. Of these, 15 were also included in the picture/telemetry datasets. No lynx died within the same 1‐month interval in which they were first detected.

#### 2.3.3 Jura Population

For the Jura lynx population, we included data from all identified individuals who had at least one record in the two subareas (Southern Jura, Northern Jura; Figure 1). We included all data between 1 May 1997 and 30 April 2022 (Table 1). To account for substantial trapping effort differences between months, data were grouped into capture occasions of 2‐month intervals within each “lynx year” (according to lynx reproductive cycle from 1st May to 30th of April). For data selection, we again followed the same procedure as for the Alpine population. Resident transboundary individuals with France who were regularly detected on both sides of the border were not considered as dispersers. These individuals were identified by hand using data available from a scientific collaboration on transboundary monitoring with the French authorities (KORA/OFB, unpublished data). We considered only records on the Swiss side for the analysis, and we corrected for the resulting lower detection probability for transboundary individuals by including individual as a random factor in the model for detection probability (see model description below). Data of individuals that were removed from the Jura population in the frame of translocation projects were censored at the date of translocation. The final data set contained 8598 pictures of 364 individuals, of which 20 individuals were observed by means of telemetry (Table 1) yielding a total of 10,942 fixes.

Age, sex, and birth year were treated as for the Alpine population. We considered individuals that had been detected outside the two subareas only once and then moved back as if they had always been inside. To do so, we deleted the single record outside from the data (2 such cases). Including individuals as a random factor in the model for detection probability accounts for temporary migration (see model description below).

One hundred and fifty‐one lynx were found dead in the Jura population during the study period. Of these, 45 were also included in the picture/telemetry datasets.

### 2.4 Description of survival models

We combined a multi‐state model fitted to picture, telemetry, and dead recovery data with an age‐at‐death model fitted to life‐span data obtained from lynx found dead. To do so, we assume that the lynx in all data sets are coming from the same lynx population; thus, they have the same average survival rates. Age at death models would usually require that the probability of detecting a dead animal does not depend on age. To account for this bias, we used the age‐specific probabilities of finding a dead lynx, estimated in the multi‐state model, for correcting unequal detection probabilities among age classes in the age‐at‐death model.

#### 2.4.1 Multi‐State Model

**FIGURE 2:**
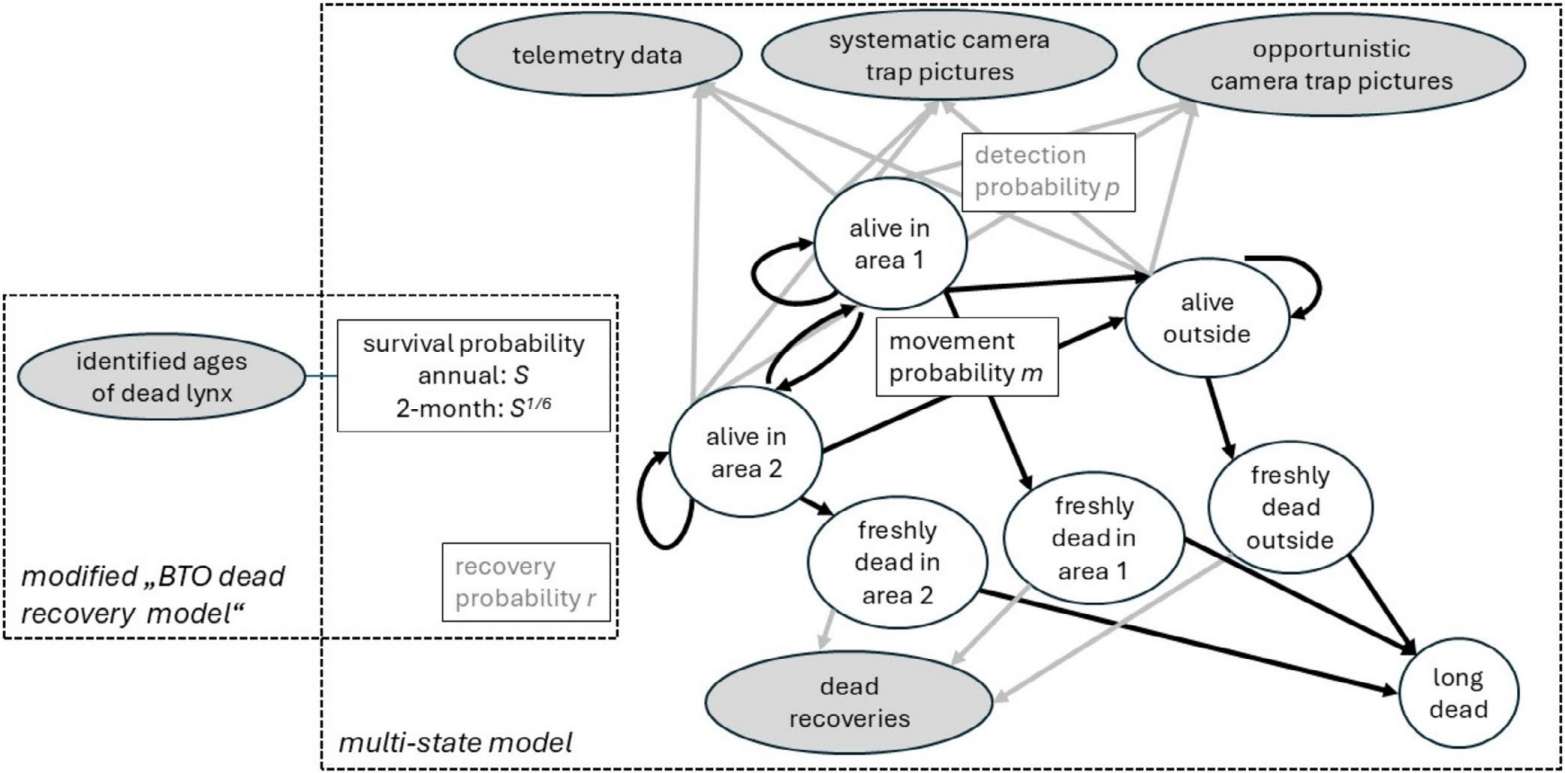
Graphical representation of the integration of the two models (modified “BTO dead recovery model” and the multi‐state model). Data is depicted as shaded round boxes, model parameters are given in squared boxes, partially observed or latent state variables or given in round boxes. Black arrows are transitions between states that are defined by survival and movement probabilities (ecological process), gray arrows indicate the observation process defined by detection and recovery probabilities. The model parameters shared among the two models are survival and recovery probability.

We used a multi‐state model (Choquet et al. 2009) from the mark‐recapture modeling framework that allows for disentangling detection probability and survival (Lebreton et al. 1992; Thomson et al. 2009). Separate models were fitted to each of the three lynx populations. Because we aimed at estimating survival at the level of subareas, we allowed movements between the two subareas of the Alpine and Jura populations, respectively. We further combined dead recoveries with live re‐sightings (Lebreton et al. 1999) and allowed for permanent emigration from all three study populations. As a consequence, the estimated survival probabilities can be considered to be true survival (corrected for emigration) rather than apparent survival. Immigration (e.g., movement between the three study populations) was not considered in our models because (as opposed to emigration) this process is less relevant for estimating survival within one given area but complicates the model and increases the uncertainty of parameter estimates. The workflow diagram in Figure 2 shows how the different data sources contributed to the estimation of model parameters.

The model used for the Northeastern Switzerland population allowed for movements to outside the study population with no back‐migration allowed and assumed that lynx can be in 5 states:

1. Alive inside Northeastern Switzerland
2. Alive outside Northeastern Switzerland
3. Died within that month inside Northeastern Switzerland
4. Died within that month outside Northeastern Switzerland
5. Dead before that month (no longer detectable)

The model used for the Alpine and Jura populations allowed for bidirectional movement between two connected subareas (Alps: Northwestern Swiss Alps and Central Switzerland; Jura: Northern and Southern Jura; see Figure 1) and for movements to outside the study population (= anywhere outside the two subareas) with no back‐migration allowed. The model assumed 7 states:

1. Alive inside subarea 1
2. Died within that 2‐month period inside subarea 1
3. Alive inside subarea 2
4. Died within that 2‐month period inside subarea 2
5. Alive outside the two subareas
6. Died within that 2‐month period outside the two subareas
7. Dead before that 2‐month period (no longer detectable)

We hereafter describe the multi‐state model for the more complex case of two subareas (subarea 1 and 2) within the study population and an area defined as “outside” (= subarea 3, i.e., all records that do not lie within subarea 1 or 2) as was used for the Alpine and Jura lynx populations (see Supporting Information for the exact structure of the model used for the Northeastern Switzerland population). Subarea numbers are given as subscripts to model parameters. We assumed that states changed from one capture occasion *t* to the next *t* + 1 according to the two‐months (or 1‐month) survival probability s and movement probabilities *m* as defined for individual *i* in the transition matrix *T*, where *m*12 and *m*21 are the probabilities to move between subarea 1 and subarea 2, and *m*13 and *m*23 are the probabilities to permanently emigrate from subareas 1 and 2 within a capture occasion. Two‐months (and 1‐month) survival probabilities *s* were assumed to be annual survival *S* probability powered by 1/6 (or 1/12).

$T=\left[\begin{array}{ccccccc} s_{i,t}\left(1-m12_{i,t}-m13_{i,t}\right) & 1-s_{i,t} & s_{i,t}m12_{i,t} & 0 & m13_{i,t}s_{i,t} & 0 & 0\\ 0 & 0 & 0 & 0 & 0 & 0 & 1\\ s_{i,t}m21_{i,t} & 0 & s_{i,t}\left(1-m21_{i,t}-m23_{i,t}\right) & 1-s_{i,t} & s_{i,t}m23_{i,t} & 0 & 0\\ 0 & 0 & 0 & 0 & 0 & 0 & 1\\ 0 & 0 & 0 & 0 & s_{i,t} & 1-s_{i,t} & 0\\ 0 & 0 & 0 & 0 & 0 & 0 & 1\\ 0 & 0 & 0 & 0 & 0 & 0 & 1 \end{array}\right]$

Bidirectional movements were possible between subareas, but once an individual emigrated (movement to “outside” = subarea 3) back‐migration was not possible (permanent emigration). Temporary emigration and transboundary animals were accounted for by including individuals as a random effect on detection probability.

We temporally discretised the survival and movement process in 2‐monthly (or monthly) intervals and assumed that mortality events take place before movement within these capture occasions. As a consequence, lynx can only be found dead in the location they have been at the beginning of the capture occasion. This assumption reduces the complexity of the transition probabilities and helps make the model identifiable. Based on whether an individual is alive or dead, it can either be detected by camera trapping or telemetry with the probability *p*, or its carcass can be recovered with probability *r* within each capture occasion. The observation matrix *O* defines for each state (rows) the probability to be detected or not (columns), and is defined by *p* and *r*.

$O=\left[\begin{array}{ccccccc} p_{i,t} & 0 & 0 & 0 & 0 & 0 & 1-p_{i,t}\\ 0 & r_{i,t} & 0 & 0 & 0 & 0 & 1-r_{i,t}\\ 0 & 0 & p_{i,t} & 0 & 0 & 0 & 1-p_{i,t}\\ 0 & 0 & 0 & r_{i,t} & 0 & 0 & 1-r_{i,t}\\ 0 & 0 & 0 & 0 & p_{i,t} & 0 & 1-p_{i,t}\\ 0 & 0 & 0 & 0 & 0 & r_{i,t} & 1-r_{i,t}\\ 0 & 0 & 0 & 0 & 0 & 0 & 1 \end{array}\right]$

We used the logit link function to relate the predictors to annual survival *S*, detection *p*, and recovery probability *r*. We estimated separate survival probabilities between the two subareas and outside to allow for spatial differences in survival. Within each area, we estimated separate annual survival probabilities for each age and sex class, and we included the year as a random factor to account for among‐year variance in survival (*σ*_*S*_ × *γ*_*v*_). The survival in year *v*, for individual *i*, is defined by:

$$\text{logit}\left(S_{1,i,v}\right)=b_{01}\left[{\mathrm{sex}}_{i},{\mathrm{age}}_{i,v}\right]+\sigma_{\text{S1}}\times \gamma_{1,v}$$

$\text{logit}\left(S_{2,i,v}\right)=b_{02}\left[{\mathrm{sex}}_{i},{\mathrm{age}}_{i,v}\right]+\sigma_{\text{S2}}\times \gamma_{2,v}$

$$\text{with}\;\gamma_{\text{area},v}∼\text{normal}\left(0,1\right)$$

For survival outside, we estimated only an overall mean, logit(s_3,i,v_) = *b*_03_, because the sample size was low in this area and survival after permanent emigration was not the focus of our study. Only for the Northeastern Switzerland population with no subareas, we allowed for age‐specific random year effects on survival:

$$\text{logit}(S_{\mathrm{age},i,v)}=b_{0}\left[{\mathrm{sex}}_{i},{\mathrm{age}}_{i,v}\right]+\sigma_{S}\left[{\mathrm{age}}_{i,v}\right]\times \gamma_{\mathrm{age},v}$$

Different detection probabilities were estimated between the two subareas and outside. We allowed detection probability to be different between sexes, age classes, and between lynx with and without active telemetry devices in all three areas. We further included two predictors that measured observation effort. First, the variable *systmon* indicated whether a systematic camera‐trap monitoring had taken place within a subarea during the time interval (0/1 per month and subarea, here averaged over the subarea and sampling occasion). Second, the variable *oppmon* measured the intensity of opportunistic monitoring measured on a scale from 1 to 3 for each canton and year. For a given year, we averaged this value over the cantons within each (sub)area. A random individual effect was added to account for individual differences in detection probabilities, for example, caused by different overlap of their home ranges with the two subareas (e.g., individuals transboundary with France) or differences in activity. We assumed that the effects of *systmon* and *oppmon* were similar in both subareas.

$$\begin{array}{cc} \text{logit}\left(p_{1,i,t}\right)\; & =a_{01}\left[{\mathrm{sex}}_{i},{\mathrm{age}}_{i,v},{\text{telemetry}}_{i,t}\right]+a_{1}\times \text{monitoring}1_{t}\\ & +a_{2}*{\text{oppeff1}}_{t}+\sigma_{p}\epsilon_{i} \end{array}$$

$\begin{array}{c} \text{logit}\left(p_{2,i,t}\right)=a_{02}\left[{\mathrm{sex}}_{i},{\mathrm{age}}_{i,v},{\text{telemetry}}_{i,t}\right]+a_{1}\times \text{monitoring}2_{t} \end{array}$ 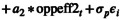


In the area outside the two subareas (subarea 3), we estimated detection probabilities per age and sex class for lynx with and without an active VHF/GPS collar within each time interval.

$$\text{logit}\left(p_{3,i,t}\right)=a_{03}\left[{\mathrm{sex}}_{i},{\mathrm{age}}_{i,v},{\text{telemetry}}_{i,t}\right]$$

For lynx with an active VHF/GPS collar, we estimated detection probability independent of age and sex by constraining the intercepts to be equal among age and sex classes:

*a*_0area_[2,2:3,2] = *a*_0area_[1,1,2] for the two subareas and outside (Jura and Alps), and for Northeastern Switzerland and outside, respectively. For the Northeastern Switzerland population, we fixed this parameter to 1 because all individuals with active VHF/GPS collars had been localized in each month.

The probability of getting recovered if dead is estimated for each sex and age class and was allowed to vary between the two subareas and outside, except for the Northeastern Switzerland population where only one individual was found dead outside. For that population, we assumed equal dead recovery probability inside and outside (*logit*(*r*_*i*,*t*_) = *d*_0_[sex_*i*_, age_i,*v*_]).

The dead recovery probabilities for the three subareas in the Alps and Jura mountains were estimated for each sex and age class separately (intercept *d*_*0*_ is sex and age‐specific) with an additive effect of subarea (*d*_*1,2*_ is the difference between subarea 2 and subarea 1 and *d*_1,3_ is the difference between subarea 3 and subarea 1).

$$\text{logit}\left(r_{1,i,t}\right)=d_{0}\left[{\mathrm{sex}}_{i},{\mathrm{age}}_{i,v}\right]$$

$\text{logit}\left(r_{2,i,t}\right)=d_{0}\left[{\mathrm{sex}}_{i},{\mathrm{age}}_{i,v}\right]+d_{1,2}$

$$\text{logit}\left(r_{3,i,t}\right)=d_{0}\left[{\mathrm{sex}}_{i},{\mathrm{age}}_{i,v}\right]+d_{1,3}$$

The probability to move among the two subareas and outside is estimated separately for each sex and age class. For individuals with unknown sex, we modeled sex as a categorical variable sex_*i*_ ∼ categorical (*ψ*), where *ψ* is a vector containing the proportion of females and males. Thereby, individuals with unknown sex were assigned a probability of belonging to one of the two sexes according to the sex ratio given by the data.

We modeled the observations *y*_*i*,*t*_ as a categorical variable dependent on the latent state variable *z*_*i*,*t*_ and the observation matrix *O*.

$$y_{i,t}∼\text{categorical}\left(O\left[z_{i,t},i,t,\right]\right)$$

The state variable is modeled as a Markovian categorical variable *z*_*i*,*t*_ ∼ *categorical*(*T*[*z*_*i*,*t*−1_,*i*,*t* − 1]) with *z*_*i*,first*[i]*_ = *y*_*i*,first*[i]*_ and *first*_*i*_ is the state the lynx was captured or pictured for the first time, i.e., alive in one of the two subareas within the study population for all individuals.

As prior distributions, we used uniform distributions for movement probability so that the sum of all movements from one subarea did not exceed 1, *normal* (0.1.5^2^) for intercepts in the linear predictors for survival, detection, and recovery, and *normal* (0.5^2^) for coefficients of covariates. For variance parameters, we use folded t‐distributions, *t* ^+^(0.1,2). The flat Dirichlet distribution is used as a prior for the proportion of females and males.

#### 2.4.2 Model for Age‐at‐Death Data

For the age‐at‐death data we used the “BTO‐dead recovery model” (Cooch and White 2016; Lerche‐Jørgensen et al. 2018) to estimate annual survival for the three age classes and the two sexes. This model is designed for estimating survival between discrete age classes based on dead recoveries when the number of marked individuals is not known and it is not possible to estimate recovery probability. The model assumes that the probability that a dead individual is found does not depend on age class. This bias was addressed by combining the dead recovery model with the multi‐state model (see next section). We modeled the sex for individuals with unknown sex as a latent variable using the same categorical model as described in the multi‐state model.

We modeled the age at death *y* ^lifetime^ as a categorical variable, where each year after birth is a category *j* out of 18, which was the maximal age of lynx found dead in our dataset.

*y* ^lifetime^ ∼ categorical (*θ*), where *θ* is a vector containing, for every year after birth, the probability that an individual lynx will be found dead during that year of its life. The probability of dying and being found at age *j*, given that the lynx dies within 18 years, is expressed as *θ*_i,j_ = ∏(s_i,1:(j−1)_)(1 − s_i,j_)/(1 − ∏(s_i,1:18_)). Thus, the model estimates age‐specific annual survival from the distribution of age at death. We estimated separate annual survival for each sex and age class and assumed uniform prior distributions for annual survival. The same software, jags, was used to fit the model as was used for the multi‐state model.

#### 2.4.3 Combination of Both Models

We combined the multi‐state model (picture, capture, telemetry, dead recovery data) and the age at death model in an integrated model, assuming that age‐and sex‐specific annual survival was the same for both models. The advantage of the model combination is that the probability of detecting a dead lynx for each age class can be estimated from the multi‐state model, and we can use that estimate to correct the survival estimate from the age at death model for age‐dependent detection of dead individuals. The probability to die and be found at age *j*, given that the lynx dies within 18 years, is then modified to:

$$\theta_{i,j}=\prod \left(s_{i,1:\left(j-1\right)}\right)\left(1-s_{i,j}\right)r_{i,j}/P_{i}$$

where *P*_*i*_ is the probability that a lynx dies and is found within *T* ^max^ = 18 years. Thus, the model assumes that no lynx gets older than 18 years (maximum life span documented in the study area). *P*_*i*_ is obtained from survival *s* and the probability to recover a dead lynx *r*, and it differs between the two sexes because of different survival and different detection probabilities.

$$P_{i}=\left(1-s_{i,1}\right)r_{i,1}+s_{i,1}\left(1-s_{i,2}\right)r_{i,2}+s_{i,1}s_{i,2}\left(1-s_{i,3}\right)r_{i,3}\;\frac{\left(s_{i,3}^{T^{\max-2}}-1\right)}{\left(s_{i,3}-1\right)}$$

We fitted the model using Markov Chain Monte Carlo simulations as implemented in jags (Plummer 2003). We used a marginalized likelihood for fitting the model to increase the speed of model fitting (Yackulic et al. 2020). We accessed jags from the software R 4.0.5 (R Core Team 2021) via the package R2jags (Su and Yajima 2020). We assessed the convergence of the Markov chains by visual checking of the traceplots, by the r‐hat values and the number of effective samples, which were above 2000 for most parameters. We present the means of the posterior distributions as point estimates of the model parameters and the 2.5% and 97.5% quantiles as lower and upper limits of the 95% uncertainty interval.

## 3 Results

### 3.1 Alpine population

**FIGURE 3:**
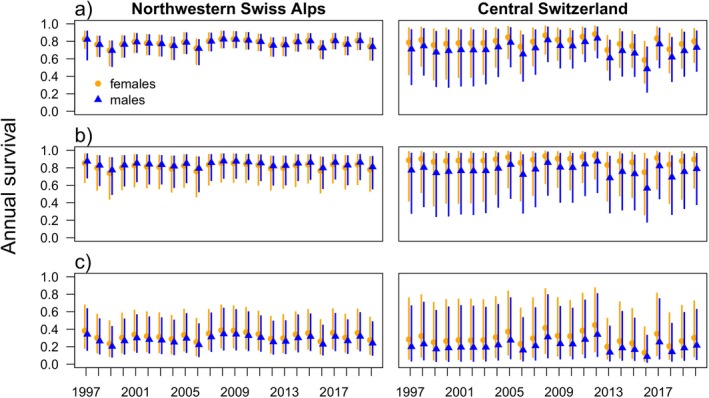
Estimated annual survival probability for (a) adults (> 2 years) (b) subadults (1–2 years) and (c) juveniles (< 1 year) for the Alpine lynx population. Orange = females, blue = males. Left side: Estimates for Northwestern Swiss Alps; right side: Estimates for Central Switzerland. Vertical bars show 95% Compatibility Intervals. Annual survival is calculated from 2‐monthly survival within a “lynx‐year” (e.g., from 1st May 2010 to 30th April 2011).

Estimated annual survival increased with age from a juvenile survival of around 0.3 to an adult survival of around 0.8 in both sexes in the Northwestern Swiss Alps and in females of Central Switzerland. In males of Central Switzerland, survival increased from 0.20 in juveniles to 0.71 in adults (Table 2). Females seemed to survive similarly well in both subareas (survival estimate 0.78 in both subareas), whereas males survived better in the Northwestern Swiss Alps compared to Central Switzerland (survival estimate: 0.78 vs. 0.71, Table 2). In the Northwestern Swiss Alps, there was an around 10% drop in survival of all age classes in the late 1990's followed by periods with increasing survival in the early 2000s and between 2007 and 2011. Between 2011 and 2013, there was another slight drop in survival for all age classes in the Northwestern Swiss Alps. In Central Switzerland, adult survival was rather stable between 2000 and 2013. In the last decade, the among‐year variance in survival increased in both subareas and was higher in Central Switzerland compared to the Northwestern Swiss Alps (Figure 3). Lowest estimated annual adult survival was 0.49 (95% CI: 0.21–0.74) in the year 2016 for males in Central Switzerland. Juvenile survival was below 0.4 on average (Table 2) and showed high variance and high levels of uncertainty (Figure 3).

**TABLE 2:** Estimated annual survival (*s*), detection probability from opportunistic monitoring (*p*) and dead recovery probability (*r*) in the two subareas of the Alpine lynx population (Northwestern Swiss Alps, Central Switzerland). CI = confidence intervals. Outside study population = anywhere outside Northwestern Swiss Alps or Central Switzerland.

Sex	Age class	Northwestern Swiss Alps			Central Switzerland			Outside study population
		*s* [mean (CI)]	*p* [mean (CI)]	*r* [mean (CI)]	*s* [mean (CI)]	*p* [mean (CI)]	*r* [mean (CI)]	*r* [mean (CI)]
Females	Juvenile	0.33 (0.17–0.57)	0.40 (0.27–0.54)	0.11 (0.05–0.25)	0.28 (0.11–0.57)	0.25 (0.09–0.52)	0.02 (0.01–0.09)	0.00 (0.00–0.07)
	Subadult	0.82 (0.61–0.94)	0.11 (0.07–0.16)	0.13 (0.04–0.45)	0.89 (0.58–0.98)	0.09 (0.03–0.19)	0.03 (0.00–0.18)	0.00 (0.00–0.10)
	Adult	0.78 (0.74–0.83)	0.12 (0.10–0.15)	0.13 (0.09–0.20)	0.78 (0.67–0.88)	0.11 (0.08–0.17)	0.03 (0.01–0.08)	0.00 (0.00–0.08)
Males	Juvenile	0.29 (0.15–0.51)	0.20 (0.11–0.32)	0.07 (0.03–0.17)	0.20 (0.06–0.48)	0.32 (0.12–0.62)	0.02 (0.00–0.06)	0.00 (0.00–0.05)
	Subadult	0.84 (0.66–0.82)	0.16 (0.11–0.22)	0.33 (0.11–0.77)	0.77 (0.44–0.95)	0.14 (0.06–0.28)	0.09 (0.02–0.44)	0.00 (0.00–0.27)
	Adult	0.78 (0.74–0.82)	0.19 (0.16–0.23)	0.15 (0.10–0.22)	0.71 (0.58–0.82)	0.14 (0.10–0.20)	0.04 (0.01–0.09)	0.00 (0.00–0.09)

The average time independent lynx (i.e., subadults and adults pooled) survived in the Northwestern Swiss Alps was 4.1 years for females and 4.4 years for males. In Central Switzerland, independent females survived for 4.7 years and males for 3.2 years on average. Whether a systematic monitoring took place within the 2‐month period clearly increased the probability that a lynx was detected (difference in the log‐odds: 0.45 [0.40–0.50]). The intensity of the opportunistic monitoring did not correlate with the detection probability (difference in the log‐odds: 0.00 [−0.11–0.12]).

Movement probabilities (i.e., probabilities for bidirectional movement between subareas and/or permanent emigration from the study population) were not the focus of this analysis but were calculated to obtain true survival estimates rather than apparent survival. We present here the values for the age class most likely to move, i.e., juveniles. Most movements of juveniles occurred during the dispersal phase right at the transition from their first to their second year of life (between March and June) and are considered first‐year movements by the model. Two‐monthly movement probabilities from the Northwestern Swiss Alps to Central Switzerland were 0.01 for juvenile females and 0.02 for juvenile males, whereas from Central Switzerland to the Northwestern Swiss Alps, they were 0.08 for juvenile females and 0.06 for juvenile males. Annual emigration probabilities from the study area to outside were also lower in the Northwestern Swiss Alps (0.01 for juveniles) than in Central Switzerland, where juvenile males permanently emigrated with a probability of 0.07 and juvenile females with a probability of 0.04. All other age classes had lower emigration probabilities. All parameters for monitoring effort and movement probabilities are presented in the Supporting Information.

### 3.2 Northeastern Switzerland Population

**FIGURE 4:**
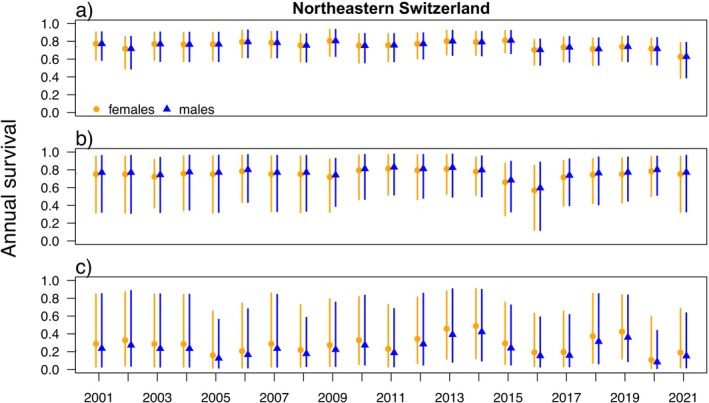
Estimated annual survival probability for (a) adults (> 2 years) (b) subadults (1–2 years) and (c) juveniles (< 1 year) based on the combined model for the Northeastern Switzerland lynx population. Orange = females, blue = males. Circles = model estimates, vertical bars = 95% compatibility intervals. Annual survival is calculated from monthly survival within a “lynx‐year” (e.g., from 1st May 2010 to 30th April 2011).

Annual survival increased from around 0.25 (95% CI: 0.07–0.67) in juveniles to 0.76 (0.66–0.85) in later age classes and was similar between males and females, with high levels of uncertainty (Table 3). Among‐year variation in annual survival decreased with age (Figure 4). There was a 20% drop in annual survival around 2016, visible for all age classes. Juvenile survival was below 0.4 on average (Table 3) and was especially low (< 0.2) in the last 2 years (2020/2021) (Figure 4). Also, in adults, survival decreased to 0.6 towards the end of the study period. However, due to the smaller size of the Northeastern Switzerland lynx population, compatibility intervals were wider than for the Alpine population, especially for the younger age classes. The average time independent lynx survived in the Northeastern Switzerland lynx population was 3.7 years for both females and males.

**TABLE 3:** Estimated annual survival (*S*), detection probability from opportunistic monitoring (*p*) and dead recovery probability (*r*) for the Northeastern Switzerland lynx population. CI = compatibility intervals.

Sex	Age class	*S* [mean (CI)]	*p* [mean (CI)]	*r* [mean (CI)]
Females	Juvenile	0.29 (0.08–0.67)	0.43 (0.23–0.64)	0.3 (0.03–0.47)
	Subadult	0.75 (0.67–0.84)	0.53 (0.4–0.72)	0.52 (0.121–0.86)
	Adult	0.76 (0.67–0.84)	0.55 (0.6–0.73)	0.08 (0.02–0.19)
Males	Juvenile	0.4 (0.07–0.3)	0.69 (0.47–0.84)	0.07 (0.02–0.0)
	Subadult	0.7 (0.53–0.92)	0.57 (0.37–0.75)	0.08 (0.01–0.38)
	Adult	0.76 (0.66–0.85)	0.63 (0.44–0.79)	0.21 (0.10–0.37)

Whether a systematic monitoring took place within the 1‐month period increased the probability that a lynx was detected (increase in the log‐odds: 1.02 [0.70–1.34]) The correlation of the intensity of the opportunistic monitoring with the detection probability was negative (−0.67 [−1.03 – −0.29]). Lynx outside of the study area had a much smaller detection probability than those inside (coefficient: −2.48 (−3.47 – −1.55)). The annual emigration probabilities from the study area to outside were 0.14 for juvenile males and 0.006 for juvenile females. All other age classes had lower emigration probabilities. For all results on monitoring effort and movement probabilities, see Supporting Information.

### 3.3 Jura population

**FIGURE 5:**
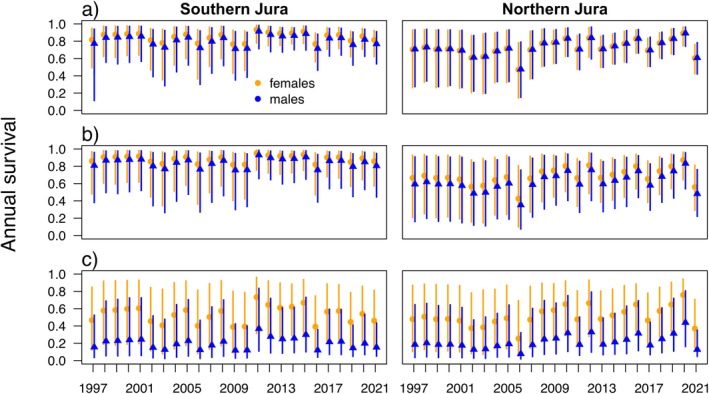
Estimated annual survival probability for (a) adults (> 2 years) (b) subadults (1–2 years) and (c) juveniles (< 1 year) based on the combined model for the Jura lynx population. Orange = females, blue = males. Left side: Estimates for the Southern Jura; right side: Estimates for the Northern Jura. Vertical bars show 95% compatibility intervals. Annual survival is calculated from 2‐monthly survival within a “lynx‐year” (e.g., from 1st May 2010 to 30th April 2011).

The among‐year variance in survival was similar in both subareas (Figure 5). Estimated annual survival increased with age class (from around 0.5 to 0.85 [Southern Jura] and 0.70 [Northern Jura] in females, and from 0.19 to 0.81 [Southern Jura] and 0.71 [Northern Jura] in males) and was higher in the Southern Jura compared to the Northern Jura and higher in females compared to males (Table 4). In both subareas, there was a 20%–30% drop in the survival of all age classes around 2006 and in 2021. Survival of juvenile females was much higher than that of juvenile males and higher than in the Alpine or NE‐CH populations (above 0.4 on average (Table 4)) but also showed high variance and high levels of uncertainty (Figure 5). The average time independent lynx survived in the Southern Jura was 6.6 years for females and 5.1 years for males. In the Northern Jura, independent females remained in the population for 2.8 years and males for 2.6 years on average.

**TABLE 4:** Estimated annual survival (S), detection probability from opportunistic monitoring (*p*) and dead recovery probability (*r*) in the two subareas of the Jura lynx population (Southern Jura, Northern Jura). CI = compatibility intervals. Outside study population = anywhere outside Southern and Northern Jura.

Sex	Age class	Southern Jura			Northern Jura			Outside study population
		*S* [mean (CI)]	*p* [mean (CI)]	*r* [mean (CI)]	*S* [mean (CI)]	*p* [mean (CI)]	*r* [mean (CI)]	*r* [mean (CI)]
Females	Juvenile	0.53 (0.26–0.80)	0.44 (0.23–0.69)	0.25 (0.08–0.67)	0.48 (0.23–0.76)	0.21 (0.11–0.37)	0.21 (0.07–0.62)	0.03 (0.00–0.20)
	Subadult	0.89 (0.78–0.91)	0.13 (0.05–0.25)	0.33 (0.12–0.67)	0.66 (0.42–0.86)	0.14 (0.08–0.24)	0.28 (0.10–0.68)	0.04 (0.00–0.26)
	Adult	0.85 (0.78–0.91)	0.11 (0.07–0.16)	0.16 (0.09–0.27)	0.70 (0.58–0.81)	0.19 (0.12–0.29)	0.13 (0.07–0.23)	0.02 (0.00–0.08)
Males	Juvenile	0.19 (0.08–0.41)	0.39 (0.19–0.62)	0.06 (0.02–0.16)	0.19 (0.08–0.43)	0.42 (0.22–0.67)	0.05 (0.02–0.14)	0.01 (0.00–0.03)
	Subadult	0.84 (0.60–0.95)	0.09 (0.04–0.21)	0.45 (0.15–0.87)	0.59 (0.33–0.81)	0.50 (0.27–0.75)	0.40 (0.13–0.85)	0.06 (0.01–0.38)
	Adult	0.81 (0.71–0.90)	0.16 (0.11–0.24)	0.22 (0.12–0.37)	0.71 (0.58–0.82)	0.40 (0.30–0.51)	0.18 (0.10–0.31)	0.02 (0.00–0.09)

Whether a systematic monitoring took place within the 2‐month period clearly increased the probability that a lynx was detected (difference in the log‐odds: 0.17 [0.11–0.24]). Also, the intensity of the opportunistic monitoring was positively correlated with detection probability (difference in the log‐odds: 0.58 [0.30–0.85]). Two‐monthly movement probabilities from Southern to Northern Jura were 0.10 for juvenile females and 0.06 for juvenile males, whereas from Northern to Southern Jura they were 0.02 for juvenile females and 0.08 for juvenile males. Annual emigration probabilities from the study area to outside were higher for juvenile females than for males in the Southern Jura (juvenile females: 0.38, juvenile males: 0.23) but lower for juvenile females than for males in the Northern Jura (juvenile females: 0.20, juvenile males: 0.33). All other age classes had lower emigration probabilities. All results on monitoring effort and movement probabilities are presented in the Supporting Information.

## 4 Discussion

Understanding the factors influencing the survival of predators living in anthropogenic landscapes is crucial for their conservation management (Goodrich et al. 2008; López‐Bao et al. 2019). However, the necessary data is often hard to acquire for long‐lived and elusive wildlife species such as large carnivores (Brongo et al. 2005). Our study provides information on the long‐term development of survival rates in three reintroduced Swiss lynx populations. Our results reveal differences in survival between sex and age classes, (sub)populations and temporal patterns.

### 4.1 Differences between sexes, age classes and populations

#### 4.1.1 Juvenile Survival

Juvenile survival was lower on average than in the other age classes (below 0.4 in most subpopulations) but showed high variability among years and high levels of uncertainty of model predictions. Juvenile survival is known to be lower (e.g., bobcat *Lynx rufus* ; Jones et al. 2020; Lehman et al. 2024) and more variable than adult survival in lynx and other wild felid species (e.g., Andrén et al. 2006; cougar *Puma concolor* , Clark et al. 2015) and can be dependent on prey availability (e.g., Eurasian lynx, Walton et al. 2017; Canada *lynx Lynx canadensis*, Brand and Keith 1979). Further factors known to influence kitten survival are litter size (e.g., Eurasian lynx, Gaillard et al. 2014), predation (e.g., cheetah *Acinonyx jubatus* , Laurenson et al. 1995), infanticide (e.g., cougar, Clark et al. 2015) or high inbreeding levels (e.g., Florida panther *Puma concolor coryi* , Hostetler et al. 2010; captive cheetah, Wielebnowski 1996). For the Eurasian lynx, there are few studies based on telemetry that report juvenile survival (but see Andrén et al. 2006). Previous radio‐telemetry studies from the Southern Jura and Northwestern Swiss Alps found juvenile survival rates of 0.45–0.54 Northwestern Swiss Alps; Breitenmoser‐Würsten et al. 2001) and 0.43–0.49 (Southern Jura; Breitenmoser‐Würsten et al. 2007), compared to 0.17–0.59 in this study. In most subpopulations of our study, we found rather low average values for juvenile survival that were similar to values found in Scandinavian lynx populations subject to hunting and illegal killing (0.22–0.59, Andrén et al. 2006) or in a wild population of Florida panthers suffering from inbreeding depression (0.32, Hostetler et al. 2010). The lynx is protected in Switzerland, but illegal killing is regularly reported (Foundation KORA 2021). Moreover, all reintroduced Swiss lynx populations have lost genetic variability due to few founder individuals and isolation (Mueller et al. 2022) which might impact juvenile survival (Bean et al. 2004). Detailed analyses of causes of death and possible inbreeding effects on juvenile survival are in progress (KORA/FIWI, unpublished data).

Juvenile females in our study tended to have a slightly higher survival than juvenile males, with highest difference in survival rates in the Jura. In many mammals, early survival is lower in males (“fragile male hypothesis”), especially in species where males are the larger sex (Clutton‐Brock et al. 1985; Kraemer 2000). Males being the heterogametic sex, they cannot compensate deleterious alleles on their sex chromosomes (Smith 1989; Smith and Warner 1989). In this context, the emergence of potentially congenital heart anomalies in Swiss lynx populations (Ryser‐Degiorgis et al. 2020), which seems more frequent in male lynx (I. Marti, unpublished data), warrants further investigation. In our model, the first part of the dispersal phase at the end of the first year of life is included in juvenile survival. Therefore, dispersal‐related mortality such as traffic accidents also contribute to low juvenile survival rates, especially in males (as in other felids such as bobcat, Blankenship et al. 2006). This seems to be most pronounced in the Jura. While adult female survival is the demographic parameter with the strongest influence on population growth for wild felids (e.g., *Puma concolor* , Lambert et al. 2010; Benson et al. 2011), kitten survival can also be influential (Hostetler et al. 2013; Clark et al. 2015). In order to assure long‐term conservation of the lynx in Switzerland, ongoing studies on mortality causes in juvenile lynx will provide important information (e.g., Borel et al. unpublished data; Niehaus 2025).

#### 4.1.2 Subadult Survival

The average survival of subadults was between 0.60 (subadult males, Northern Jura) and 0.92 (subadult females, Northwestern Swiss Alps) and slightly higher for females than for males in all subpopulations except Central Switzerland. Survival between subadults and adults was similar but, as for juvenile survival, the uncertainty of the model predictions was high.

In solitary wild felid species, subadults often show similar survival rates to adults (e.g., leopards *Panthera pardus* , Swanepoel et al. 2015; Florida panthers, Benson et al. 2011) or survival rates in between juveniles and adults (e.g., bobcat, Jones et al. 2020; Lehman et al. 2024; Benson et al. 2020). Transient animals (i.e., mostly dispersing subadults) have been shown to have lower survival rates than residents and were more likely to die from vehicle collisions in some felid species (e.g., bobcat, Blankenship et al. 2006; ocelot *Leopardus pardalis* , Haines et al. 2005). In Eurasian lynx, subadult survival is variable among years, and the number of transient “floaters” can account for high between‐year variation in population counts (Breitenmoser and Breitenmoser‐Würsten 2008; Palmero et al. 2021). Moreover, a study based on telemetry reported highly variable subadult survival among different study areas (e.g., ranging from 0.43 to 0.9 including natural mortality and suspected illegal killing; Andrén et al. 2006). Female subadults seemed to have higher survival than males, although this was not the case in all Scandinavian study areas (Andrén et al. 2006). In a pan‐European study on lynx survival based on radio‐telemetry, subadult survival was 0.77 for females and 0.8 for males in protected populations and nearly 1 for females and 0.73 for males in hunted populations (Premier et al. 2024). Previous telemetry studies from Switzerland found subadult survival rates around 0.53 (Southern Jura; Breitenmoser‐Würsten et al. 2007) and 0.44 (Northwestern Swiss Alps; Breitenmoser‐Würsten et al. 2001).

Subadult males in Northern Jura had the lowest survival compared to other subareas. The Northern Jura is located at the edge of the Jura Mountain chain at the transition to strongly populated lowland areas to the North and West. Low subadult survival could be linked to higher human‐caused mortality for young lynx dispersing into these areas, for example, through vehicle collisions. However, emigration probabilities to outside the study population were not higher for subadults in Northern compared to Southern Jura (see Supporting Information). This suggests that either mortality of subadults is higher inside Northern Jura (warranting further investigation as in Borel et al. unpublished data) or that information on lynx dispersing and/or dying outside Northern Jura was incomplete.

#### 4.1.3 Adult Survival

Average annual adult survival ranged between 0.71 (adult males and females, Northern Jura) and 0.85 (adult females, Southern Jura). Survival of males and females was similar for most subpopulations, with slightly higher survival of adult females in Southern Jura and Central Switzerland. Adult lynx had the lowest survival in Northern Jura compared to the other subpopulations. There was less uncertainty in model predictions for adult lynx compared to the other age classes due to the larger sample size.

Adult female survival is the most influential parameter for population growth in wild felid species (Lambert et al. 2010; Benson et al. 2011) and natural mortality of adults is usually low in Eurasian lynx, especially for adult females (e.g., survival rates including only natural mortality: 0.95–1, Andrén et al. 2006; survival rates of independent lynx: 0.82 (males, protected areas), 0.9 (females, protected areas), Palmero et al. 2021; adult survival rates: 0.85 (males, protected areas), 0.89 (females, protected areas), Premier et al. 2024). In hunted felid populations, adult mortality increases since resident animals (e.g., adults) are most likely to get shot (e.g., Andrén et al. 2006; Blankenship et al. 2006). This can have an impact on population growth: a study on lynx harvest in Scandinavia suggests that hunting quotas above 20% of the counted population were associated with a population decline (Linnell et al. 2010). Simulations from a simple population model (no immigration/emigration processes included) for a lynx population in Central Europe suggested that adult mortality below 30% would still permit population growth (assuming juvenile mortality up to 50%, Červený et al. 2019). In our study, adult survival was similar to lynx populations subject to illegal killing and hunting (0.77–0.86, Andrén et al. 2006; 0.79 (males), 0.85 (females), Premier et al. 2024) and also similar to previous Swiss studies based on radio‐telemetry (Southern Jura: 0.76, Breitenmoser‐Würsten et al. 2007; Northwestern Swiss Alps: 0.72, Breitenmoser‐Würsten et al. 2001). The potential longevity of the species in the wild is 15–18 years (KORA/FIWI, unpublished data) but most lynx never reach old age. If we look at the average number of years a lynx survived after reaching its second year of life, we see that in most Swiss subpopulations, independent lynx (subadults and adults pooled) persisted for 3–5 years on average. In the Southern Jura, females survived especially long (> 6 years) and in the Northern Jura, the average survival time was lowest (< 3 years). This means that on average, adult females in Northern Jura only reproduce once or twice in their lives, suggesting that the Northern Jura population may be a potential sink sustained by the more vital Southern Jura population, which has recently expanded on the French side and recolonized new areas (Arpin et al. 2024; Molinari‐Jobin et al. 2024).

The lynx is a protected species in Switzerland and no legal hunting occurs, although individual lynx causing damage to livestock may be removed by the authorities (BAFU 2016). Our results suggest that additional sources of adult mortality (e.g., human‐caused mortality or increased natural mortality due to infectious diseases) exist in the reintroduced Swiss lynx populations, especially in the Northern Jura, Northeastern Switzerland, and the Northwestern Swiss Alps. A detailed analysis of mortality causes in Swiss lynx populations is in preparation (Borel et al. unpublished data) and will shed more light on the role of anthropogenic factors (e.g., road collisions or high levels of illegal killing as reported from some areas of Switzerland; Arlettaz et al. 2021). To ensure the long‐term survival of Switzerland's reintroduced lynx populations, further studies are needed to disentangle natural and anthropogenic causes of mortality. Studies evaluating the potential relationship between inbreeding and survival are underway (KORA/FIWI, unpublished data). Potential natural mortality could be caused by observed health issues such as potentially congenital heart anomalies (Ryser‐Degiorgis et al. 2020) or emerging infectious diseases such as Feline Immunodeficiency Virus (FIV) and Feline Leukemia Virus (FeLV) (Marti et al. 2021; Ryser‐Degiorgis et al. 2021).

### 4.2 Temporal patterns

Subadult survival followed similar patterns as adult survival, while juvenile survival was highly variable over the years. For the discussion of temporal patterns, we focus on adult survival. In the Alps, fluctuations in adult survival were different in the Northwestern Swiss Alps compared to Central Switzerland, which corresponds to differences in population development between the two subpopulations (Kunz et al. 2021a, 2021b). There was a drop in survival in the Northwestern Swiss Alps in the late 1990s, which corresponds to a period of increased lynx –human conflict with high levels of damage to livestock leading to legal killings of damage‐causing lynx and to the occurrence of demonstrative acts of illegal killing (Foundation KORA 2021). An increase in survival between 2007 and 2010 in both Northwestern Swiss Alps and Central Switzerland corresponds to increasing population numbers observed in this period (Kunz et al. 2021a, 2021b; Sterrer et al. 2023b). In both subpopulations, but especially in Central Switzerland, we observe an increased between‐year variance in survival during the last 10 years. In the Northwestern Swiss Alps, this increased variance could be caused by the population reaching carrying capacity, as lynx densities are currently high (Kunz et al. 2021a). However, this explanation does not hold for Central Switzerland (Kunz et al. 2021b; Sterrer et al. 2023b), where there must be another explanation for high between‐year variance in mortality that could potentially be related to illegal killing or individual factors (e.g., health or genetics).

In Northeastern Switzerland, adult survival was rather stable during the phase of population establishment until it dropped in 2016. Since then, survival rates have remained slightly lower and dropped again in 2021. The timespan between 2008 and 2014 marks a period of population growth and range expansion of the Northeastern Switzerland lynx population (Sterrer et al. 2022; Foundation KORA 2024a). Between 2016 and 2021, however, population growth slowed, and there was an increase in reported mortalities (Foundation KORA 2024a). Moreover, four female lynx were translocated from Northeastern Switzerland to the LIFElynx project Pfälzerwald/Vosges du Nord between 2017 and 2020 (Signer et al. 2021). The question remains whether natural mortality has increased due to the population reaching carrying capacity or whether inbreeding, emerging health problems, or human‐caused mortality have decreased survival. A detailed analysis of the causes of death between 2016 and 2021 is underway (i.e., genetic investigation of fatal soft tissue mineralization in juvenile and subadult lynx; Niehaus 2025) and will help to assess the status of the lynx population in Northeastern Switzerland.

In the Jura population, adult survival fluctuated in the early 2000s with a marked drop, especially in the Northern Jura, in 2006. During this phase, the Jura lynx population was still growing (Foundation KORA 2024b). The potential reasons for low survival in 2006 remain unclear. Adult survival stayed stable at high levels between 2011 and 2015 in the Southern Jura, which corresponds to a phase of stable population numbers (Le Grand et al. 2021), but was fluctuating periodically at lower levels in the Northern Jura. After 2016, adult survival increased in the Northern Jura, which corresponds to a population increase (Le Le Grand et al. 2021), but dropped again in 2021. The lynx habitat of the Swiss part of the Jura mountains is continuous between the Southern and the Northern Jura, and the lynx population is considered to be saturated (Drouet‐Hoguet et al. 2021). However, the Northern Jura subpopulation showed a slower population development than the Southern Jura, and lynx densities are still lower (Le Grand et al. 2021; Le Grand et al. 2022). Our results suggest that additional causes of mortality exist in the Northern Jura that have decreased survival and slowed population growth in this subpopulation, potentially turning it into a sink. The hunting system differs between the Southern and Northern Jura. While all Cantons in the Southern Jura practice a license hunting system, in three out of five Cantons in the Northern Jura, a territory hunting system prevails (BAFU 2021). Contrary to the territory hunting system, where hunting territories are rented to hunting groups for fees related to realized quotas, the hunting season is restricted to a few weeks in the licence hunting system, and hunters are free to hunt on the entire territory of the Canton. This leads to an intense but very short period of hunting pressure in the licence hunting system, as opposed to a more prolonged hunting period in the territory system. In the licence hunting system, state‐employed game wardens are responsible for the control of hunting, while this function is fulfilled by the hunting associations themselves in some territory hunting systems. These differences may potentially have an influence on local game densities, on hunters attitudes towards lynx, or on the risk of illegal killing. A recent study from the Bavarian‐Bohemian lynx population showed that female apparent survival was lowest in hunting grounds rented to local hunting societies and higher in state‐managed hunting grounds or protected areas (Mináriková et al. 2024). Illegal killing is thought to be a threat for the Bavarian‐Bohemian lynx population (Heurich et al. 2018) and opportunities for illegal killing are likely linked to the type of hunting system.

### 4.3 Differences in detection and dead recovery probabilities among populations

Inter‐individual variance in detection probability was high, indicating that this parameter captured variance in movement (i.e., temporary migration) between individuals. Systematic monitoring sessions clearly increased detection probability in all three study populations but most strongly in Northeastern Switzerland. This result shows that systematic camera trapping (widely applied for felid species; Rovero and Zimmermann 2016) is an effective tool for monitoring lynx. Opportunistic monitoring effort was not correlated with detection probability in the Alps, negatively correlated in Northeastern Switzerland, and positively correlated in the Jura, where the correlation was even stronger than for systematic monitoring. Opportunistic monitoring effort was measured at a rather crude spatial scale (at the level of Cantons) which may not capture true variation in some areas. However, our findings could also highlight that opportunistic monitoring effort should be increased in the Alps and that its efficiency in Northeastern Switzerland should be evaluated in more detail.

Dead recovery probabilities were lower in Central Switzerland than in the other populations. This may be related to this subarea holding a large proportion of inaccessible Alpine terrain where carcasses are not found. The highest dead recovery probabilities were estimated for subadults in all three populations. One potential explanation could be that this age class is more vulnerable to anthropogenic mortality associated with a higher detection probability (such as vehicle collisions) but further studies on causes of mortality are needed.

## 5 Conclusions

In this study, we use a novel approach allowing us to combine picture data from camera trapping and chance observations, telemetry data, and dead recoveries over a monitoring period of 25 years. Our study provides important baseline data for future studies such as population viability analyses and highlights that survival varies strongly between subpopulations. Future studies on mortality causes (e.g., human‐caused mortality) and potential effects of inbreeding on survival are needed to ensure long‐term conservation of the reintroduced Swiss lynx populations.

Our survival estimates correspond to observed population trends and demonstrate the complexity and high variation of survival between different age and sex classes in space and time, potentially leading to source sink dynamics. Switzerland holds the largest part of the Alpine and an important part of the Jura lynx population and plays an important role in the colonization of yet unoccupied lynx habitats in neighboring countries. Our results highlight that understanding source‐sink dynamics and connectivity between subpopulations is crucial to ensure viable lynx metapopulations in Europe.

## Author Contributions

**K. Vogt:** conceptualization (lead), data curation (equal), formal analysis (supporting), project administration (equal), validation (equal), writing – original draft (lead). **F. Korner‐Nievergelt:** data curation (equal), formal analysis (lead), methodology (lead), visualization (lead), writing – original draft (equal). **S. Signer:** data curation (equal), formal analysis (supporting), visualization (equal), writing – original draft (supporting). **F. Zimmermann:** data curation (equal), project administration (equal), validation (equal), writing – original draft (equal). **I. Marti:** data curation (equal), validation (supporting), writing – original draft (supporting). **A. Ryser:** data curation (equal), validation (supporting), writing – original draft (supporting). **A. Molinari‐Jobin:** data curation (equal), validation (equal), writing – original draft (equal). **U. Breitenmoser:** conceptualization (equal), funding acquisition (equal), project administration (equal), writing – original draft (supporting). **Ch. Breitenmoser‐Würsten:** conceptualization (equal), data curation (equal), funding acquisition (lead), project administration (equal), validation (equal), writing – original draft (equal).

## Conflicts of Interest

The authors declare no conflicts of interest.

## Supporting information


Appendix S1.

## Data Availability

Data and Supporting Information are available from the Dryad Digital Repository: Vogt, Kristina (Forthcoming 2025). Dataset: Long‐term changes in survival of Eurasian lynx in three reintroduced populations in Switzerland [Dataset]. Dryad. https://doi.org/10.5061/dryad.573n5tbhw

## References

[ece371095-bib-0001] Andrén, H. , N. T. Hobbs , M. Aronsson , et al. 2020. “Harvest Models of Small Populations of a Large Carnivore Using Bayesian Forecasting.” Ecological Applications 30, no. 3: e02063. 10.1002/eap.2063. 31868951 PMC7187313

[ece371095-bib-0002] Andrén, H. , J. D. C. Linnell , O. Liberg , et al. 2006. “Survival Rates and Causes of Mortality in Eurasian lynx ( *Lynx lynx* ) in Multi‐Use Landscapes.” Biological Conservation 131: 23–32.

[ece371095-bib-0003] Arlettaz, R. , G. Chapron , M. Kéry , et al. 2021. “Poaching Threatens the Establishment of a Lynx Population, Highlighting the Need for a Centralized Judiciary Approach.” Frontiers in Conservation Science 2: 665000. 10.3389/fcosc.2021.665000.

[ece371095-bib-0004] Arpin, I. , F. Sarrazin , G. Bal , et al. 2024. “Expertise scientifique collective sur la viabilité des populations de lynx boréal en France. Rapport Final.” OFB; MNHN. 2024, pp. 242 hal‐04811130v2.

[ece371095-bib-0005] Von Arx, M. , C. Breitenmoser‐Würsten , F. Zimmermann , et al. 2017. “Der Luchs im Jura–Unter besonderer Berücksichtigung des Solothurner Juras.” Mitteilungen der Naturforschenden Gesellschaft Des Kantons Solothurn 43: 177–234.

[ece371095-bib-0006] BAFU (Bundesamt für Umwelt) . 2016. Konzept Luchs Schweiz. Vollzugshilfe des BAFU zum Luchsmanagement in der Schweiz, 23. BAFU.

[ece371095-bib-0007] BAFU (Bundesamt für Umwelt) . 2021. “Eidgenössische Jagdstatistik–Jagdsysteme.” https://www.jagdstatistik.ch/de/huntsystems.

[ece371095-bib-0008] Basille, M. , I. Herfindal , H. Santin‐Janin , et al. 2009. “What Shapes Eurasian lynx Distribution in Human Dominated Landscapes: Selecting Prey or Avoiding People?” Ecography 32: 683–691. 10.1111/j.1600-0587.2009.05712.x.

[ece371095-bib-0009] Bean, K. , W. Amos , P. P. Pomeroy , S. D. Twiss , T. N. Coulson , and I. L. Boyd . 2004. “Patterns of Parental Relatedness and Pup Survival in the Grey Seal ( *Halichoerus grypus* ).” Molecular Ecology 13: 2365–2370. 10.1111/j.1365-294X.2004.02199.x. 15245408

[ece371095-bib-0010] Benson, J. F. , J. A. Hostetler , D. P. Onorato , et al. 2011. “Intentional Genetic Introgression Influences Survival of Adults and Subadults in a Small, Inbred Felid Population.” Journal of Animal Ecology 80: 958–967. 21338353 10.1111/j.1365-2656.2011.01809.x PMC6993175

[ece371095-bib-0011] Benson, J. F. , J. A. Sikich , and S. P. D. Riley . 2020. “Survival and Competing Mortality Risks of Mountain Lions in a Major Metropolitan Area.” Biological Conservation 241: 108294.

[ece371095-bib-0012] Blankenship, T. L. , A. M. Haines , M. E. Tewes , and N. J. Silvy . 2006. “Comparing Survival and Cause‐Specific Mortality Between Resident and Transient Bobcats *Lynx rufus* .” Wildlife Biology 12: 297–303.

[ece371095-bib-0013] Boitani, L. , and J. D. C. Linnell . 2015. “Bringing Large Mammals Back: Large Carnivores in Europe.” In Rewilding European Landscapes, edited by H. M. Pereira and L. M. Navarro , 67–84. Springer.

[ece371095-bib-1003] Borel, S. , C. Breitenmoser , F. Origgi , et al. Unpublished data. “Causes of Mortality and Morbidity in Free‐Ranging Eurasian Lynx (*Lynx lynx*) in Switzerland, 2000–2022.” 10.1371/journal.pone.0344107 PMC13012485 41875172

[ece371095-bib-0015] Brand, C. J. , and L. B. Keith . 1979. “Lynx Demography During a Snowshoe Hare Decline in Alberta.” Journal of Wildlife Management 43: 827–849.

[ece371095-bib-0016] Breitenmoser, U. 1998. “Large Predators in the Alps: The Fall and Rise of Man's Competitors.” Biological Conservation 83, no. 3: 279–289. 10.1016/S0006-3207(97)00084-0.

[ece371095-bib-0017] Breitenmoser, U. , and C. Breitenmoser‐Würsten . 2008. Der Luchs. Ein Grossraubtier in der Kulturlandschaft. Salm Verlag.

[ece371095-bib-0018] Breitenmoser, U. , and H. Haller . 1993. “Patterns of Predation by Reintroduced European lynx in the Swiss Alps.” Journal of Wildlife Management 57: 135–144. 10.2307/3809010.

[ece371095-bib-0020] Breitenmoser‐Würsten, C. , and G. Obexer‐Ruff . 2003. “Population and Conservation Genetics of Two Re‐Introduced lynx ( *Lynx lynx* ) Populations in Switzerland—A Molecular Evaluation 30 Years After Translocation.” Environmental Encounters 58: 51–55.

[ece371095-bib-0021] Breitenmoser‐Würsten, C. , J.‐M. Vandel , F. Zimmermann , and U. Breitenmoser . 2007. “Demography of *lynx Lynx lynx* in the Jura Mountains.” Wildlife Biology 13: 381–392.

[ece371095-bib-0022] Breitenmoser‐Würsten, C. , F. Zimmermann , A. Ryser , et al. 2001. “Untersuchungen zur Luchspopulation in den Nordwestalpen der Schweiz 1997–2000.” KORA Report No. 9, 1–89.

[ece371095-bib-0023] Brongo, L. L. , M. S. Mitchell , and J. B. Grand . 2005. “Long‐Term Analysis of Survival, Fertility, and Population Growth Rate of Black Bears in North Carolina.” Journal of Mammalogy 86: 1029–1035.

[ece371095-bib-0024] Červený, J. , J. Krojerová‐Prokešová , T. Kušta , and P. Koubek . 2019. “The Change in the Attitudes of Czech Hunters Towards Eurasian lynx: Is Poaching Restricting lynx Population Growth?” Journal for Nature Conservation 47: 28–37. 10.1016/j.jnc.2018.11.002.

[ece371095-bib-0025] Chapron, G. , P. Kaczensky , J. D. C. Linnell , et al. 2014. “Recovery of Large Carnivores in Europe's Modern Human‐Dominated Landscapes.” Science 346, no. 6216: 1517–1519. 10.1126/science.1257553. 25525247

[ece371095-bib-0026] Choquet, R. , L. Rouan , and R. Pradel . 2009. “Program E‐Surge: A Software Application for Fitting Multievent Models.” In Modeling Demographic Processes in Marked Populations. Environmental and Ecological Statistics, edited by D. L. Thomson , E. G. Cooch , and M. J. Conroy , 845–865. Springer. 10.1007/978-0-387-78151-8.

[ece371095-bib-0027] Clark, D. A. , B. K. Johnson , and D. H. Jackson . 2015. “Monthly and Annual Survival Rates of Cougar Kittens in Oregon.” Northwest Science 89: 393–400.

[ece371095-bib-0028] Clutton‐Brock, T. H. , S. D. Albon , and F. E. Guinness . 1985. “Parental Investment and Sex Differences in Juvenile Mortality in Birds and Mammals.” Nature 313: 131–133.

[ece371095-bib-0029] Cooch, E. G. , and G. C. White . 2016. “Program MARK: A Gentle Introduction.” http://www.phidot.org/software/mark/docs/book/.

[ece371095-bib-0030] Drouet‐Hoguet, N. , D. Chenesseau , F. Kunz , and F. Zimmermann . 2021. “Situation of the lynx in the Jura Mountains.” Cat News Special Issue 14: 29–34.

[ece371095-bib-0031] Foundation KORA . 2020. “Jahresbericht 2019.” pp. 24.

[ece371095-bib-0032] Foundation KORA . 2021. “50 Years of Lynx Presence in Switzerland.” KORA Report No. 99e, pp. 90.

[ece371095-bib-0033] Foundation KORA . 2024a. “Das Projekt LUNO–Abschlussbericht. KORA Report No. 121 (in German).”

[ece371095-bib-0034] Foundation KORA . 2024b. “Lynx records from 2000 to 2006.” www.koracenter.ch.

[ece371095-bib-0035] Foundation KORA . 2024c. “Lynx: Abundance Switzerland.” https://www.kora.ch/en/species/lynx/abundance.

[ece371095-bib-0036] Foundation KORA . 2024d. “Lynx: Distribution Switzerland.” https://www.kora.ch/en/species/lynx/distribution.

[ece371095-bib-0037] Gaillard, J.‐M. , E. B. Nilsen , J. Odden , H. Andrén , and J. D. C. Linnell . 2014. “One Size Fits all: Eurasian lynx Females Share a Common Optimal Litter Size.” Journal of Animal Ecology 83: 107–115. 23859302 10.1111/1365-2656.12110

[ece371095-bib-0038] Gajdárová, B. , E. Belotti , L. Bufka , et al. 2021. “Long‐Distance Eurasian lynx Dispersal–A Prospect for Connecting Native and Reintroduced Populations in Central Europe.” Conservation Genetics 22: 799–809. 10.1007/s10592-021-01363-0.

[ece371095-bib-0039] Goodrich, J. M. , L. L. Kerley , E. N. Smirnov , et al. 2008. “Survival Rates and Causes of Mortality of Amur Tigers on and Near the Sikhote‐Alin Biosphere Zapovednik.” Journal of Zoology 276: 323–329. 10.1111/j.1469-7998.2008.00458.x.

[ece371095-bib-0040] Haines, A. M. , M. E. Tewes , L. L. Laack , W. E. Grant , and J. Young . 2005. “Evaluating Recovery Strategies for an Ocelot ( *Leopardus pardalis* ) Population in the United States.” Biological Conservation 126: 512–522.

[ece371095-bib-0041] Hansen, A. J. 2011. “Contribution of Source‐Sink Theory to Protected Area Science.” In Sources, Sinks, and Sustainability Across Landscapes, edited by J. Liu , V. Hull , A. Morzillo , and J. Wiens , 339–360. Cambridge University Press.

[ece371095-bib-0042] Herdtfelder, M. , U. Schraml , and R. Suchant . 2021. “Steps Towards a lynx Population in the Black Forest?” Cat News Special Issue 14: 45–46.

[ece371095-bib-0043] Heurich, M. , J. Schultze‐Naumburg , N. Piacenza , et al. 2018. “Illegal Hunting as a Major Driver of the Source‐Sink Dynamics of a Reintroduced lynx Population in Central Europe.” Biological Conservation 224: 355–365. 10.1016/j.biocon.2018.05.011.

[ece371095-bib-0044] Hostetler, J. A. , D. P. Onorato , D. Jansen , and M. K. Oli . 2013. “A Cat's Tale: The Impact of Genetic Restoration on Florida Panther Population Dynamics and Persistence.” Journal of Animal Ecology 82: 608–620. 23252671 10.1111/1365-2656.12033

[ece371095-bib-0045] Hostetler, J. A. , D. P. Onorato , J. D. Nichols , et al. 2010. “Genetic Introgression and the Survival of Florida Panther Kittens.” Biological Conservation 143: 2789–2796. 21113436 10.1016/j.biocon.2010.07.028 PMC2989677

[ece371095-bib-0046] Jobin, A. , P. Molinari , and U. Breitenmoser . 2000. “Prey Spectrum, Prey Preference and Consumption Rates of Eurasian lynx in the Swiss Jura Mountains.” Acta Theriologica 45: 243–252. 10.4098/AT.arch.00-26.

[ece371095-bib-1001] Jones, L. R. , P. A. Zollner , R. K. Swihart , E. Godollei , C. M. Hudson , and S. A. Johnsson . 2020. “Survival and Mortality Sources in a Recovering Population of Bobcats (*Lynx rufus*) in South‐Central Indiana.” American Midland Naturalist 184, no. 2: 222–232.

[ece371095-bib-0047] KAWA (Amt für Wald des Kantons Bern) . 2015. “Waldinventur des Kantons Bern (WNI): Ergebnisbericht 1994 bis 2012.” Bern, pp. 1–28. (in German).

[ece371095-bib-0048] Kraemer, S. 2000. “The fragile male.” BMJ 321: 1609–1612. 11124200 10.1136/bmj.321.7276.1609 PMC1119278

[ece371095-bib-0050] Kunz, F. , L. Le Grand , E. Ziegler , R. Bürki , and F. Zimmermann . 2021a. “Fang‐Wiederfang‐Schätzung der Abundanz und Dichte des Luchses im Referenzgebiet Simme‐Saane IVa im Winter 2020/21.” KORA Report No. 103, pp. 16.

[ece371095-bib-0049] Kunz, F. , L. Le Grand , G. Beuchat , and F. Zimmermann . 2021b. “Fang‐Wiederfang‐Schätzung der Abundanz und Dichte des Luchses in der Zentralschweiz West IIIa im Winter 2020/21.” KORA Report No. 102, pp. 14.

[ece371095-bib-0051] Lambert, C. S. , R. B. Wielgus , H. S. Robinson , et al. 2010. “Cougar Population Dynamics and Viability in the Pacific Northwest.” Journal of Wildlife Management 70: 246–254.

[ece371095-bib-0052] Laurenson, M. K. , N. Wielebnowski , and T. M. Caro . 1995. “Extrinsic Factors and Juvenile Mortality in Cheetahs.” Conservation Biology 9: 1329–1331. 34261268 10.1046/j.1523-1739.1995.9051327.x-i1

[ece371095-bib-0053] Le Grand, L. , F. Kunz , N. Fischer , G. Beuchat , and F. Zimmermann . 2021. “Estimation par capture‐recapture photographique de l'abondance et densité du lynx dans le sud du Jura durant l'hiver 2020/21.” KORA Report No. 101, pp. 14.

[ece371095-bib-0054] Le Grand, L. , U. Sterrer , F. Kunz , et al. 2022. “Fang‐Wiederfang‐Schätzung der Abundanz und Dichte des Luchses im Jura Nord Ib im Winter 2021/22.” KORA Report No. 110 (de), pp. 11.

[ece371095-bib-0055] Lebreton, J.‐D. , T. Almeras , and R. Pradel . 1999. “Competing Events, Mixtures of Information and Multistrata Recapture Models.” Bird Study 46: 39–46.

[ece371095-bib-0056] Lebreton, J.‐D. , K. P. Burnham , J. Clobert , and D. R. Anderson . 1992. “Modelling Survival and Testing Biological Hypotheses Using Marked Animals: A Unified Approach With Case Studies.” Ecological Monographs 62: 67–118.

[ece371095-bib-1002] Lehman, C. P. , E. E. Morrison , B. Y. Neiles , and C. T. Rota . 2024. “Factors Influencing Population Growth in a Bobcat Population.” Journal of Wildlife Management 2024, no. 88: e22561. 10.1002/jwmg.22561.

[ece371095-bib-0057] Lerche‐Jørgensen, M. , F. Korner‐Nievergelt , A. P. Tøttrup , M. Willemoes‐Kristensen , and K. Thorup . 2018. “Early Returning Long‐Distance Migrant Males Do Pay a Survival Cost.” Ecology and Evolution 8: 34–49. 10.1002/ece3.4569. PMC6303765 30598747

[ece371095-bib-0058] Linnell, J. D. C. , U. Breitenmoser , C. Breitenmoser‐Wuersten , and J. Odden . 2009. “Recovery of Eurasian lynx in Europe: What Part Has Reintroduction Played?” In Reintroduction of Top‐Order Predators, edited by M. W. Hayward and M. J. Somers , 72–91. Blackwell Publishing Ltd.

[ece371095-bib-0059] Linnell, J. D. C. , H. Broseth , J. Odden , and E. Birkeland Nilsen . 2010. “Sustainably Harvesting a Large Carnivore? Development of Eurasian Lynx Populations in Norway During 160 Years of Shifting Policy.” Environmental Management 45, no. 5: 1142–1154. 10.1007/s00267-010-9455-9. 20213233

[ece371095-bib-0060] Linnell, J. D. C. , B. Cretois , E. B. Nilsen , et al. 2020. “The Challenges and Opportunities of Coexisting With Wild Ungulates in the Human‐Dominated Landscapes of Europe's Anthropocene.” Biological Conservation 244: 108500.

[ece371095-bib-0061] Litvaitis, J. A. , G. C. Reed , R. P. Carroll , et al. 2015. “Bobcats ( *Lynx rufus* ) as a Model Organism to Investigate the Effects of Roads on Wide‐Ranging Carnivores.” Environmental Management 55: 1366–1376. 10.1007/s00267-015-0468-2. 25832342

[ece371095-bib-0062] López‐Bao, J. V. , M. Aronsson , J. D. C. Linnell , J. Odden , J. Persson , and H. Andrén . 2019. “Eurasian lynx Fitness Shows Little Variation Across Scandinavian Human‐Dominated Landscapes.” Scientific Reports 9, no. 1: 8903. 10.1038/s41598-019-45569-2. 31222101 PMC6586631

[ece371095-bib-0063] Marti, I. A. , S. R. R. Pisano , S. Signer , et al. 2021. Feline Leukemia Virus in Free‐Ranging Eurasian lynx ( *Lynx lynx* )–A pathogen to Keep an Eye on. Wildlife Disease Association (WDA)–European Wildlife Disease Association (EWDA) Virtual Conference, August 31 to September 2, 2021, Cuenca.

[ece371095-bib-0064] Marti, I. A. , and M.‐P. Ryser‐Degiorgis . 2018. “A Tooth Wear Scoring Scheme for Age Estimation of the Eurasian lynx ( *Lynx lynx* ) Under Field Conditions.” European Journal of Wildlife Research 64: 37. 10.1007/s10344-018-1198-6.

[ece371095-bib-0065] Mináriková, T. , E. Belotti , J. Krausová , et al. 2024. “Factors Influencing the Survival of Adult Eurasian lynx ( *Lynx lynx* ) Females at the Core and at the Edge of the BBA Population Distribution.” EUROLYNX Conference, 7–9 October 2024, Neuwiller‐Lès‐Saverne, France.

[ece371095-bib-0066] Molinari‐Jobin, A. 2003. “Pan‐Alpine Conservation Strategy for the Lynx.” Environmental Encounters 58: 65–66.

[ece371095-bib-0067] Molinari‐Jobin, A. , O. Anders , M. Back , et al. 2024. SCALP Monitoring Report Lynx Year 2020/2021 (1. May 2020–30. April 2021). pp. 4.

[ece371095-bib-0068] Molinari‐Jobin, A. , M. Back , S. Bauduin , et al. 2020. “SCALP Monitoring Report Lynx Year 2019/2020 (1. May 2019–30. April 2020).” pp. 5.

[ece371095-bib-0070] Molinari‐Jobin, A. , E. Marboutin , S. Wölfl , et al. 2010. “Recovery of the Alpine *lynx Lynx lynx* Metapopulation.” Oryx 44: 267–275.

[ece371095-bib-0071] Mueller, S. A. , S. Prost , O. Anders , et al. 2022. “Genome‐Wide Diversity Loss in Reintroduced Eurasian lynx Populations Urges Immediate Conservation Management.” Biological Conservation 266: 109442. 10.1016/j.biocon.2021.109442.

[ece371095-bib-0072] Nagl, D. , U. Breitenmoser , K. Hackländer , et al. 2022. “Long‐Term Changes in Habitat Selection and Prey Spectrum in a Reintroduced Eurasian lynx ( *Lynx lynx* ) Population in Switzerland.” Ecology and Evolution 12: e8614. 35228862 10.1002/ece3.8614 PMC8861841

[ece371095-bib-0073] Niehaus, J. 2025. “Pathological and Genetic Investigations of Severe Organ Mineralizations in the Eurasian Lynx.” Master Thesis. University of Greifswald, Germany.

[ece371095-bib-0074] Palmero, S. , E. Belotti , L. Bufka , et al. 2021. “Demography of a Eurasian lynx ( *Lynx lynx* ) Population Within a Strictly Protected Area in Central Europe.” Scientific Reports 11: 19868. 10.1038/s41598-021-99337-2. 34615965 PMC8494906

[ece371095-bib-0075] Plummer, M. 2003. “JAGS: A Program for Analysis of Bayesian Graphical Models Using Gibbs Sampling JAGS: Just Another Gibbs Sampler.” In Proceedings of the 3rd International Workshop on Distributed Statistical Computing, edited by K. Hornik , F. Leisch , and A. Zeileis , 1–8. Dsc. Vienna.

[ece371095-bib-0076] Port, M. , A. Henkelmann , F. Schroder , et al. 2020. “Rise and Fall of a Eurasian lynx ( *Lynx lynx* ) Stepping‐Stone Population in Central Germany.” Mammal Research 66: 45–55. 10.1007/s13364-020-00527-6.

[ece371095-bib-0077] Premier, J. , M. Bastianelli , J. Oeser , et al. 2024. “Survival of Eurasian lynx in the Human‐Dominated Landscape of Europe.” Conservation Biology 14: e14439. 10.1111/cobi.14439. PMC12124177 39807884

[ece371095-bib-0078] R Core Team . 2021. “R: A Language and Environment for Statistical Computing.” R Foundation for Statistical Computing, Vienna, Austria. https://www.R‐project.org/.

[ece371095-bib-0079] Ripple, W. J. , J. A. Estes , R. L. Beschta , et al. 2014. “Status and Ecological Effects of the World's Largest Carnivores.” Science 343: 1241484. 10.1126/science.1241484. 24408439

[ece371095-bib-0080] Rovero, F. , and F. Zimmermann . 2016. Camera Trapping for Wildlife Research. Pelagic Publishing.

[ece371095-bib-0081] Ryser, A. , M. Scholl , M. Zwahlen , M. Oetliker , M.‐P. Ryser‐Degiorgis , and U. Breitenmoser . 2005. “A Remote‐Controlled Teleinjection System for the Low‐Stress Capture of Large Mammals.” Wildlife Society Bulletin 33: 721–730.

[ece371095-bib-0082] Ryser, A. , K. von Wattenwyl , F. Zimmermann , and U. Breitenmoser . 2006. “2. Monitoringbericht LUNO2–Status Luchs Nordostschweiz Winter 2005/2006.” KORA Report No. 34, pp. 24.

[ece371095-bib-0083] Ryser‐Degiorgis, M.‐P. , I. Marti , S. R. R. Pisano , et al. 2021. “Management of Suspected Cases of Feline Immunodeficiency Virus Infection in Eurasian Lynx ( *Lynx lynx* ) During an International Translocation Program.” Frontiers in Veterinary Science 8: 730874. 10.3389/fvets.2021.730874. 34760956 PMC8573149

[ece371095-bib-0084] Ryser‐Degiorgis, M.‐P. , N. Robert , R. K. Meier , et al. 2020. “Cardiomyopathy Associated With Coronary Arteriosclerosis in Free‐Ranging Eurasian Lynx ( *Lynx lynx* Carpathicus).” Frontiers in Veterinary Science 7: 1103. 10.3389/fvets.2020.594952 PMC7779598 33409296

[ece371095-bib-0085] Signer, S. , A. Ryser , M.‐P. Ryser‐Degiorgis , et al. 2021. “Translocation de Lynx depuis la Suisse vers la forêt du Palatinat et les Kalkalpen (2016–2020).” KORA Report No. 100, pp. 26.

[ece371095-bib-0086] Smith, D. W. E. 1989. “Is Greater Female Longevity a General Finding Among Animals.” Biological Reviews, Cambridge Philosophical Society 64: 1–12. 10.1111/j.1469-185x.1989.tb00635.x 2655725

[ece371095-bib-0087] Smith, D. W. E. , and H. R. Warner . 1989. “Does Genotypic Sex Have a Direct Effect on Longevity?” Experimental Gerontology 24: 277–288. 2684675 10.1016/0531-5565(89)90001-6

[ece371095-bib-0088] Sterrer, U. , L. Le Grand , F. Kunz , R. Fitze , B. Lengwiler , and F. Zimmermann . 2023a. Estimation par capture‐recapture photographique de l'abondance et densité du lynx dans l'aire de référence Sud du Jura (la) durant l'hiver 2022/23. KORA Report No. 118 (fr), pp. 13.

[ece371095-bib-0089] Sterrer, U. , L. Le Grand , F. Kunz , M. Rüegg , and F. Zimmermann . 2022. “Fang‐Wiederfang‐Schätzung der Abundanz und Dichte des Luchses in der Nordostschweiz II im Winter 2021/22.” KORA Report No. 109, pp. 11.

[ece371095-bib-0090] Sterrer, U. , L. Le Grand , B. Lengwiler , R. Fitze , J. Amrhein , and F. Zimmermann . 2023b. “Fang‐Wiederfang‐Schätzung der Abundanz und Dichte des Luchses im Referenzgebiet Zentralschweiz Mitte IIIb im Winter 2022/23.” KORA‐Bericht 120, pp. 12.

[ece371095-bib-0091] Su, Y.‐S. , and M. Yajima . 2020. R2jags: Using r to Run ‘JAGS’. https://CRAN.R‐project.org/package=R2jags.

[ece371095-bib-0092] Swanepoel, L. H. , M. J. Somers , M. Schiess‐Meier , et al. 2015. “Survival Rates and Causes of Mortality of Leopards *Panthera pardus* in Southern Africa.” Oryx 49: 595–603.

[ece371095-bib-0093] Swiss Federal Authorities . 2021a. “The Jura.” https://www.eda.admin.ch/aboutswitzerland/en/home/umwelt/geografie/jura.html.

[ece371095-bib-0094] Swiss Federal Authorities . 2021b. “Swiss Plateau.” https://www.eda.admin.ch/aboutswitzerland/en/home/umwelt/geografie/mittelland.html.

[ece371095-bib-0095] Swiss Federal Authorities . 2023. “The Alps.” https://www.eda.admin.ch/aboutswitzerland/en/home/umwelt/geografie/alpen.html.

[ece371095-bib-0096] Thomson, D. L. , E. G. Cooch , and M. J. Conroy . 2009. “Environmental and Ecological Statistics Series: Volume 3.” In Modelling Demographic Processes in Marked Populations. Springer.

[ece371095-bib-0097] Vogt, K. , E. Hofer , A. Ryser , M. Kölliker , and U. Breitenmoser . 2016. “Is There a Trade‐Off Between Scent Marking and Hunting Behaviour in a Stalking Predator, the Eurasian lynx, *Lynx lynx* ?” Animal Behaviour 117: 59–68. 10.1016/j.anbehav.2016.04.004.

[ece371095-bib-0098] von Arx, M. 2020. “ *Lynx lynx* (Amended Version of 2018 Assessment). The IUCN Red List of Threatened Species 2020: e.T12519A177350310.” 10.2305/IUCN.UK.2020-3.RLTS.T12519A177350310.en.

[ece371095-bib-0099] von Arx, M. , P. Kaczensky , J. D. C. Linnell , T. Lanz , C. Breitenmoser‐Würsten , and U. Breitenmoser . 2021. “Conservation Status of the Eurasian lynx in West and Central Europe.” Cat News Special Issue 14: 5–8.

[ece371095-bib-0100] Walton, Z. , J. Mattisson , J. D. C. Linnell , A. Stien , and J. Odden . 2017. “The Cost of Migratory Prey: Seasonal Changes in Semi‐Domestic Reindeer Distribution Influences Breeding Success of Eurasian lynx in Northern Norway.” Oikos 126: 642–650. 10.1111/oik.03374.

[ece371095-bib-0101] Wielebnowski, N. 1996. “Reassessing the Relationship Between Juvenile Mortality and Genetic Monomorphism in Captive Cheetahs.” Zoo Biology 15: 353–369.

[ece371095-bib-0102] Yackulic, C. B. , M. Dodrill , M. Dzul , J. S. Sanderlin , and J. A. Reid . 2020. “A Need for Speed in Bayesian Population Models: A Practical Guide to Marginalizing and Recovering Discrete Latent States.” Ecological Applications 30, no. 5: e02112. 10.1002/eap.2112. 32112492

[ece371095-bib-0103] Zapata, S. C. , R. G. Pereira , J. F. Beltran , P. Ferreras , and M. Delibes . 1997. “Age Determination of Iberian lynx ( *Lynx pardinus* ) Using Canine Radiograph and Cementum Annuli Enumeration.” Zeitschrift für Säugetierkunde 62: 119–123.

[ece371095-bib-0105] Zimmermann, F. 2019. “Monitoring von grossen Beutegreifern.” In Wolf, Luchs und Bär in der Kulturlandschaft. Konflikte, Chancen, Lösungen im Umgang mit grossen Beutegreifern, edited by M. Heurich , 165–200. Eugen Ulmer KG.

[ece371095-bib-0106] Zimmermann, F. , C. Breitenmoser‐Würsten , and U. Breitenmoser . 2005. “Natal Dispersal of Eurasian lynx ( *Lynx lynx* ) in Switzerland.” Journal of Zoology 267: 381–395.

[ece371095-bib-0107] Zimmermann, F. , C. Breitenmoser‐Würsten , and U. Breitenmoser . 2007. “Importance of Dispersal for the Expansion of a Eurasian *lynx Lynx lynx* Population in a Fragmented Landscape.” Oryx 41: 358–368.

[ece371095-bib-0108] Zimmermann, F. , and D. Foresti . 2016. “Capture‐Recapture Methods for Density Estimations.” In Camera Trapping for Wildlife Research, edited by F. Rovero and F. Zimmermann , 95–141. Pelagic Publishing.

[ece371095-bib-0109] Zimmermann, F. , R. Manz , J. Räber , and F. Kunz . 2020. “Fang‐Wiederfang‐Schätzung der Abundanz und Dichte des Luchses im Oberwallis IVe im Winter 2019/20.” KORA Report No. 95, pp. 23.

[ece371095-bib-0110] Zimmermann, F. , and M. von Arx . 2021. “Eurasischer Luchs.” In Säugetieratlas der Schweiz und Liechtensteins, edited by R. F. Graf and C. Fischer , 246–249. Schweizerische Gesellschaft für Wildtierbiologie SGW‐SSBF, Haupt Verlag.

