# Supplementary material for: Long‐Term Changes in Survival of Eurasian Lynx in Three Reintroduced Populations in Switzerland
Source: Ecol Evol. 2025 Mar 30;15(4):e71095. doi: 10.1002/ece3.71095 (PMC11955280; doi:10.1002/ece3.71095)
Supplement: Supplementary file 1 — Appendix S1. [file ECE3-15-e71095-s001.zip › Supplementary_material__README_ALPS.html]

Documentation and code of the survival model for the Alpine population


Code 

- Show All Code
- Hide All Code

# Documentation and code of the survival model for the Alpine population

#### Vogt et al.

#### 2025-01-10

```
library(knitr)
library(R2jags)
```

```
## Lade nötiges Paket: rjags
```

```
## Lade nötiges Paket: coda
```

```
## Linked to JAGS 4.3.1
```

```
## Loaded modules: basemod,bugs
```

```
## 
## Attache Paket: 'R2jags'
```

```
## Das folgende Objekt ist maskiert 'package:coda':
## 
##     traceplot
```

# 1 Simulation to assess bias in the survival estimation due to our treatment of L- and R-lynxes

## 1.1 Background and aim

Camera trapping of lynx is used to monitor populations. Individuals can unambiguously be identified based on pictures only after they could be pictured from both sides and a left (L) and right (R) side could be assigned to the same individual. Unmatched left and right sides were kept as “L-“ and “R-individuals” in the data. Once both sides could be assigned to the same individual, the L- and R-entries in the data base are replaced by an unambiguous B-entry (both).  
Before the assignment, a single individual could appear twice in the data, and if such an individual dies undetected, two apparent individuals disappear from the data. As a consequence, we would underestimate survival, if we kept all L- and R-individuals in the dataset. However, deleting all unmatched L- and R-individuals from the dataset would also introduce a bias, since lynx dying in their first year have a lower chance to have both sides matched than lynx who survive for several years. There are two possibilities to overcome that bias: 1) Explicitly modelling the marking process could be used to get unbiased estimates from such mark-recapture data, e.g. Maronde et al. (2020) and Nichols et al. (2004). 2) Exclude all observations before an individual could unambiguously be assigned, i.e. pretend that the first detection of an individual was at the time when both flanks could be matched Weidinger (2007). The second method has the disadvantage that sample size for estimating juvenile and subadult survival may become low.  
For the survival analyses used in Vogt et al. (in press), we could not use any of these two methods, because the information when the matching of L- and R-lynxes happened is not available, and because juvenile and subadult survival was of interest. We, therefore, used a different approach.

We deleted those single sided individuals which belonged to the less abundant group and kept the other single-sided entries as individual ids.

Here, we assess by a simulation the bias of the survival estimates with our approach.

## 1.2 Simulation

```
library(R2jags)
```

### 1.2.1 Data simulation

We assume that the probability to detect an individual on one side is \(p\). And, as a simplification, we assume that detecting an individual on the left or on the right side is independent of each other. We further assume that individuals survive from one time point to the next with survival probability \(\phi\).

We generate a state matrix \(z\) that indicates for each individual \(i\) whether it is alive or dead at time \(t\). \(z\_{i1}=1\) for all individuals, and \(z\_{it} \sim Bernoulli(z\_{it-1}\phi)\).

We then simulate capture histories for the left and right side:  
\(chL\_{it} \sim Bernoulli(z\_{it}p)\) and \(chL\_{it} \sim Bernoulli(z\_{it}p)\).

We then combine the two capture history matrices \(chL\) and \(chR\) into one capture history matrix similar to how it has been done for the lynx data set:  
1. we identified those individuals that have been detected in both capture history matrix at least once, i.e. those where \(\sum\_{t=1}^{T}{((chL\_{it}+chL\_{it}) = 2}) \geq 1\) and we call them “B-individuals”.

2. we constructed a new capture history matrix that contained all detections of the B-individuals and we added those L- and R- individuals that have only been detected on one side.

### 1.2.2 Analysis of simulated data

We then analysed the simulated data in two ways:
1) We deleted the L-individuals and analysed the rest taking the first detection of the individual as its release occasion, as we have done in our study.

2. We only used the B-individuals and assumed that the release occasion is the time when they were first detected on both sides, i.e. the time \(t\) where \(chL\_{it}+chL\_{it})=2\) for the first time.

The second method corresponds to the analyses method that produce unbiased survival estimates as has been proven before Weidinger (2007). The first method, is the one we used in Vogt et al. (in press).

### 1.2.3 Simulation settings

```
initfun <- function(){
  initz <- matrix(1, ncol=datax$nocc, nrow=datax$nind)
  for(i in 1:nrow(initz)) initz[i,1:datax$first[i]] <- NA
  list(phi=runif(1, 0,1),
       p=runif(1, 0,1),
       z=initz)
}

# repeat the simulation to measure bias
n <- 400      # initial true number of individuals 
T <- 10       # time span of the study
p <- c(0.2, 0.3)  # true detection probability
phi <- c(0.4, 0.7, 0.9) # true survival probability

dscen <- expand.grid(p=p, phi=phi) # scenarios

R <- 10 # number of simulations per scenario
estphi1 <- matrix(nrow=nrow(dscen), ncol=R)
estphi1lwr <- matrix(nrow=nrow(dscen), ncol=R)
estphi1upr <- matrix(nrow=nrow(dscen), ncol=R)

estphi2 <- matrix(nrow=nrow(dscen), ncol=R)
estphi2lwr <- matrix(nrow=nrow(dscen), ncol=R)
estphi2upr <- matrix(nrow=nrow(dscen), ncol=R)

for(k in 1:nrow(dscen)){
  for(r in 1:R){

chL <- matrix(0,ncol=T, nrow=n)
chR <- matrix(0,ncol=T, nrow=n)
z <- matrix(0,ncol=T, nrow=n)

for(i in 1:n){
  z[i,1] <- 1
  chL[i,1] <- rbinom(1,size=z[i,1],prob=dscen$p[k])
  chR[i,1] <- rbinom(1,size=z[i,1],prob=dscen$p[k])
  for(t in 2:T){
    z[i,t] <- rbinom(1, size=z[i,t-1], prob=dscen$phi[k])
    chL[i,t] <- rbinom(1,size=z[i,t],prob=dscen$p[k])
    chR[i,t] <- rbinom(1,size=z[i,t],prob=dscen$p[k])
  }
}

# A lynx is identified when it has been detected on both sides in the same time
chsum <- chL+chR
Blynxes <- apply(chsum, 1, function(x) sum(x==2)>0)
firstB <- apply(chsum[Blynxes,], 1, function(x) min(c(1:T)[x==2]))

chsum[chsum>1] <- 1
ch <- chsum[Blynxes,]
ch <- rbind(ch, chR[!Blynxes,])
todelete <- apply(ch, 1, sum)==0
ch <- ch[!todelete,]
first <- apply(ch, 1, function(x) min(c(1:T)[x==1]))
todelete <- first==T
ch <- ch[!todelete,]
firt <- first[!todelete]

datax <- list(y=ch,
              nind=nrow(ch),
              nocc=ncol(ch),
              first=first)

mod <- jags(datax, inits=initfun, 
            parameters.to.save=c("p", "phi"),
            model.file="cjs_bugs.txt", 
            n.chains=3, n.iter=5000, n.thin=1)

mod <- mod$BUGSoutput

  estphi1[k,r] <- mod$mean$phi
  estphi1lwr[k,r] <- quantile(mod$sims.list$phi, probs=0.025)
  estphi1upr[k,r] <- quantile(mod$sims.list$phi, probs=0.975)
  
  
  
ch <- chsum[Blynxes,]
todelete <- firstB==T
ch <- ch[!todelete,]
firstB <- firstB[!todelete]

datax <- list(y=ch,
              nind=nrow(ch),
              nocc=ncol(ch),
              first=firstB)
  
 mod <- jags(datax, inits=initfun, 
            parameters.to.save=c("p", "phi"),
            model.file="cjs_bugs.txt", 
            n.chains=3, n.iter=5000, n.thin=1)

mod <- mod$BUGSoutput

  estphi2[k,r] <- mod$mean$phi
  estphi2lwr[k,r] <- quantile(mod$sims.list$phi, probs=0.025)
  estphi2upr[k,r] <- quantile(mod$sims.list$phi, probs=0.975)
  }# close r
}# close K
save(estphi1, file="estphi1.rda")
save(estphi1lwr, file="estphi1lwr.rda")
save(estphi1upr, file="estphi1upr.rda")
save(estphi2, file="estphi2.rda")
save(estphi2lwr, file="estphi2lwr.rda")
save(estphi2upr, file="estphi2upr.rda")
save(dscen, file="dscen.rda")
```

## 1.3 Results and conclusions

Both approaches showed the same deviation from the true \(\phi\) value and the estimated one, averaged across all 6 scenario (combination of true \(p\) and \(\phi\) values, Figure 1.1). The maximal difference of the estimate from the true phi value was 0.03 for both approaches. The second approach can be considered to be unbiased and differences between the true values and its estimates therefore are caused by random variance in the data. Because the differences between the true values and the estimates in our approach do not exceed the differences for the unbiased second approach, we conclude that bias in our approach is negligible if at all existant. Whether the method really is unbiased may be shown mathematically. However, our mathematical expertise is too weak to create such a proof. Nevertheless, the results of this simulation make us confident that our approach does not introduce a non-negligible bias.

```
load("resultssimulation/estphi1.rda")
load("resultssimulation/estphi1lwr.rda")
load("resultssimulation/estphi1upr.rda")
load("resultssimulation/estphi2.rda")
load("resultssimulation/estphi2lwr.rda")
load("resultssimulation/estphi2upr.rda")
load("resultssimulation/dscen.rda")
R <- ncol(estphi1)

par(mfrow=c(3,2), oma=c(3,3,2,0.1), mar=c(0.1,2,2,0))
for(i in 1:nrow(dscen)){
 plot(1:(2*R), c(estphi1[i,], estphi2[i,]), ylim=c(0,1),
         las=1, ylab=NA, xlab=NA, xaxt="n", main=NA)
  if(i<=2) mtext(paste("p=", dscen$p[i]), side=3)
 abline(h=dscen$phi[i], col="grey")
 segments(1:(2*R), c(estphi1lwr[i,], estphi2lwr[i,]), 1:(2*R), c(estphi1upr[i,], estphi2upr[i,]), lwd=2)
 points(1:(2*R), c(estphi1[i,], estphi2[i,]), pch=c(rep(16, R), rep(21,R)), bg="white")
 text(5, 0.05, labels=paste("mean = ", round(mean(estphi1[i,]), 2)))
 text(15, 0.05, labels=paste("mean = ", round(mean(estphi2[i,]), 2)))
}#i

mtext("Phi", outer=TRUE, side=2, line=1)
```

Figure 1.1: Estimated phi for 10 simulated data sets where L individuals were deleted (as in our study, solid dots) and were only B-individuals and only time points after the individuals could be identified as B-individuals (open circles). Vertical bars are 95% compatibility intervals. Horizontal grey line indicate the true value that was used for simulating the data, i.e. 0.4, 0.7 and 0.9. For panels on the left side, a detection probability of 0.2, and for the panels of the right side 0.3 was assumed. The means of the estimates across the 10 simulations are given at the bottom of each panel.

# 2 Data

## 2.1 Picture and telemetry data

```
load("data/datax_ALPS.rda")
str(datax)
```

```
## List of 16
##  $ y            : num [1:540, 1:150] 7 7 7 7 7 7 7 7 7 7 ...
##  $ first        : num [1:540] 86 17 11 19 36 5 35 43 20 44 ...
##  $ sex          : num [1:540] 2 2 2 1 NA NA NA 1 NA NA ...
##  $ nind         : int 540
##  $ last         : int [1:540] 150 150 150 23 150 150 150 150 150 150 ...
##  $ age          : num [1:540, 1:25] NA NA NA NA NA 3 NA NA NA NA ...
##  $ monitoringIV : num [1:150] -0.418 -0.418 -0.418 -0.418 -0.418 ...
##  $ monitoringIII: num [1:150] -0.366 -0.366 -0.366 -0.366 -0.366 ...
##  $ oppeffIII    : num [1:150] -1.19 -1.19 -1.19 -1.19 -1.19 ...
##  $ oppeffIV     : num [1:150] 1.94 1.94 1.94 1.94 1.94 ...
##  $ telemetry    : num [1:540, 1:150] 1 1 1 1 1 1 1 1 1 1 ...
##  $ alpha        : num [1:2] 1 1
##  $ nyears       : num 25
##  $ year         : num [1:150] 1 1 1 1 1 1 2 2 2 2 ...
##  $ fr           : num [1:540] 1 1 1 1 1 1 1 1 1 1 ...
##  $ fr1          : num [1:540] 1 1 1 1 1 1 1 1 1 1 ...
```

The data object `datax` contains the following elements:

- `y`: observation matrix with one row per individual and one column per 2-month period. The entries specify the categorised observation (1 = recorded in Northwestern Swiss Alps (NSA) alive, 2 = recovered in NSA dead, 3 = recorded in Central Swiss Alps (CS) alive, 4 = recovered in CS dead, 5 = recorded outside the study area alive, 6 = recovered outside the study area dead, 7 = no seen during the 2-month period)
- `first`: 2-month period of first release or first detection of the individual
- `sex`: sex per individual (1 = female, 2 = male, NA = unknown)
- `nind`: number of individuals in the data
- `last`: last 2-month period per individual that should be included in the data. For most individuals this is the last 2-month period of the study period. For individuals that were translocated to a different study area, it is the 2-month period of translocation (censoring of data at transolcation).
- `age`: matrix with rows corresponding to individuals and columns to lynx years. Values 1 = lynx in its first year, 2 = lynx in its second year, and 3 = older than second year.
- `monitoringIV`: category of monitoring per 2-month (1 = opportunistic, 2 = deterministic) in NSA.
- `monitoringIII`: category of monitoring per 2-month (1 = opportunistic, 2 = deterministic) in CS
- `oppeffIV`: observation effort per 2-month period in NSA. The larger the value the more cameras were deployed in the study area. This variable referst to the opportunistic monitoring, thus it does not include the number of cameras of the deterministic monitoring for which a separate detection probability is estimated.
- `oppeffIII`: observation effort per 2-month period in CS. The larger the value the more cameras were deployed in the study area. This variable referst to the opportunistic monitoring, thus it does not include the number of cameras of the deterministic monitoring for which a separate detection probability is estimated.
- `telemetry`: specificator when lynxes were tagged by a telemetry device (1 = no telemetry device, 2 = with telemetry device)  
  `
- `alpha`: parameter values of prior dirichlet distribution for the proportion of females and males.
- `nyears`: number of years in the study period
- `year`: assignment of monthes to lynx years
- `fr`: frequency of the observation history
- `fr1`: auxiliary variable enabling the use of marginalised model formulation

Graphical display of age categories per individual and year:

```
image(t(datax$age))
```

Figure 2.1: Age categories (darker colour from 1 to 3) for each individual and year.

```
colkey <- c(rainbow(3), "white")
location <- c(1,1,2,2,3,3, NA)

ch <- datax$y[order(datax$first),]


par(mar=c(2,4,0.1,0.1))
plot(seq(1, ncol(datax$y), length=nrow(datax$y)),
    1:nrow(datax$y), type="n", xlab="2-month period", yaxt="n", ylab=NA)
axis(2, at=1:nrow(datax$y), 1:nrow(datax$y), las=1, cex.axis=0.6)
 nocc <- ncol(datax$y)
for(i in 1:nrow(datax$y)){
  points(1:nocc, rep(i, nocc), pch=15, col=colkey[location[ch[i,]]], cex=0.2)
  inddead <- is.element(ch[i,], c(2,4,6,8)); inddead[is.na(inddead)] <- FALSE
  if(sum(inddead, na.rm=TRUE)>0) points(c(1:nocc)[inddead], i, pch=1, cex=0.2)
  }

legend(0, nrow(ch), pch=c(rep(15, 4), 1), col=c(colkey[-4], 1), 
       legend=c("NSA",  "CS", "outside", "found dead"), bty="n", cex=0.8)
```

Figure 2.2: Histories of the individuals.

## 2.2 Age at death data

```
load("data/data_ageatdeath.rda")
str(djags)
```

```
## List of 7
##  $ y       : num [1:229] 7 2 1 7 1 1 1 1 1 6 ...
##  $ ageclass: num [1:18] 1 2 3 3 3 3 3 3 3 3 ...
##  $ sex     : num [1:229] 2 1 2 NA 1 2 2 NA 2 NA ...
##  $ area    : num [1:229] 1 1 1 1 1 1 1 1 1 1 ...
##  $ nyears  : num 18
##  $ nind    : int 229
##  $ alpha   : num [1:2] 1 1
```

```
hist(djags$y, main=NA, xlab="Age at death (y)")
```

Figure 2.3: Number of individuals found dead per age in years.

The data contain the following elements:

- `y`: age (in years) at which the lynx has been recovered dead
- `ageclass`: classification of the years in age classes (1 = first year, 2 = second year, 3 = older)
- `sex`: sex per individual (1 = female, 2 = male)
- `area`: area (1 = NSA, 2 = CS)
- `nyears`: maximum age
- `nind`: number of individuals
- `alpha`: parameter values for dirichlet prior distribution for the proportion of females and males.

# 3 Integrated model for picture, telemetry and age at death data

## 3.1 Data

```
datax <- list(y=datax$y, fr=rep(1,datax$nind), fr1=rep(1, datax$nind), 
              first=datax$first,
              sex=datax$sex,
              nind=datax$nind,
              last=datax$last,
              age=datax$age,
              monitoringIV=datax$monitoringIV,
              monitoringIII=datax$monitoringIII, 
              oppeffIII=datax$oppeffIII,
              oppeffIV=datax$oppeffIV,  
              telemetry=datax$telemetry,
              alpha=c(1,1),
              nyears=datax$nyears,
              year=datax$year,
              # age at death data
              yad=djags$y,
              ageclass=djags$ageclass,
              adsex=djags$sex,
              adnyears=djags$nyears,
              adnind=djags$nind,
              area=djags$area)
```

## 3.2 Model code

```
cat(readLines('jags/Smod_alps_btocombined_ageyearint_ragedep_marginalisation.txt'), sep = '\n')
```

```
## # integrates the multi-state model with the BTO-dead recovery model
## 
## # for the multi-state model for combining camera trap data and lynx found dead
## # data:
## ## y:   (nind x nocc)
## ##  1 = VD-W-Alps alive, 2 = VDW-Alps dead,  
## ##  3 = C-Alps alive, 4 = C-Alps dead, 5 = outside alive, 
## ##  6 = outside dead, 7 = not seen
##  
## 
## ## first: month of first capture (marking)
## ## nocc: number of capture occasions (month)
## 
## # predictors:
## ## sex: vector of length nind with values 1, 2 and NA
## ## age: matrix with 3 age classes
## 
## 
## ## latent variables
## #states z:
## ##  1 = VD-W-Alps alive, 2 = VD-W-Alps freshly dead, 
## ##  3 = C-Alps alive, 4 = C-Alps freshly dead, 5 = outside alive, 
## ##  6 = outside freshly dead, 7 = dead 
## 
## 
## # for the BTO dead recovery model
## 
## # data
## # y= vector with ages at death (1 = death in first year, 2 = death in second year, 3 = death later)
## # ad.sex = vector with sexes (1=female, 2 = male, NA=not identified)
## # ageclass = vector with ageclasses 1:3
## # ad.nyears: maximum age in years
## # ad.nind: number of individuals in data set with age at death
## 
## model{
## 
##   ## transition probabilities
## for(i in 1:nind){
##   for(t in first[i]:(last[i]-1)){
##   ps[1,i,t,1] <- (1-m12[i,t]-m13[i,t])*pow(s1[i,year[t]],1/6)
##   ps[1,i,t,2] <- (1-pow(s1[i,year[t]],1/6))*r1[i,t]
##   ps[1,i,t,3] <- m12[i,t]*pow(s1[i,year[t]],1/6)
##   ps[1,i,t,4] <- 0
##   ps[1,i,t,5] <- m13[i,t]*pow(s1[i,year[t]],1/6)
##   ps[1,i,t,6] <- 0
##   ps[1,i,t,7] <- (1-pow(s1[i,year[t]],1/6))*(1-r1[i,t])
## 
##   ps[2,i,t,1] <- 0
##   ps[2,i,t,2] <- 0
##   ps[2,i,t,3] <- 0
##   ps[2,i,t,4] <- 0
##   ps[2,i,t,5] <- 0
##   ps[2,i,t,6] <- 0
##   ps[2,i,t,7] <- 1
## 
##   ps[3,i,t,1] <- m21[i,t]*pow(s2[i,year[t]],1/6)
##   ps[3,i,t,2] <- 0
##   ps[3,i,t,3] <- (1-m21[i,t]-m23[i,t])*pow(s2[i,year[t]],1/6)
##   ps[3,i,t,4] <- (1-pow(s2[i,year[t]],1/6))*r2[i,t]
##   ps[3,i,t,5] <- m23[i,t]*pow(s2[i,year[t]],1/6)
##   ps[3,i,t,6] <- 0
##   ps[3,i,t,7] <- (1-pow(s2[i,year[t]],1/6))*(1-r2[i,t])
## 
##   ps[4,i,t,1] <- 0
##   ps[4,i,t,2] <- 0
##   ps[4,i,t,3] <- 0
##   ps[4,i,t,4] <- 0
##   ps[4,i,t,5] <- 0
##   ps[4,i,t,6] <- 0
##   ps[4,i,t,7] <- 1
## 
##   ps[5,i,t,1] <- 0
##   ps[5,i,t,2] <- 0
##   ps[5,i,t,3] <- 0
##   ps[5,i,t,4] <- 0
##   ps[5,i,t,5] <- pow(s3[i,year[t]],1/6)
##   ps[5,i,t,6] <- (1-pow(s3[i,year[t]],1/6))*r3[i,t]
##   ps[5,i,t,7] <- (1-pow(s3[i,year[t]],1/6))*(1-r3[i,t])
## 
##   ps[6,i,t,1] <- 0
##   ps[6,i,t,2] <- 0
##   ps[6,i,t,3] <- 0
##   ps[6,i,t,4] <- 0
##   ps[6,i,t,5] <- 0
##   ps[6,i,t,6] <- 0
##   ps[6,i,t,7] <- 1
## 
##   ps[7,i,t,1] <- 0
##   ps[7,i,t,2] <- 0
##   ps[7,i,t,3] <- 0
##   ps[7,i,t,4] <- 0
##   ps[7,i,t,5] <- 0
##   ps[7,i,t,6] <- 0
##   ps[7,i,t,7] <- 1
##   }
##   for(t in first[i]:last[i]){
##   po[1,i,t,1] <- p1[i,t]
##   po[1,i,t,2] <- 0
##   po[1,i,t,3] <- 0
##   po[1,i,t,4] <- 0
##   po[1,i,t,5] <- 0
##   po[1,i,t,6] <- 0
##   po[1,i,t,7] <- 1-p1[i,t]
## 
##   po[2,i,t,1] <- 0
##   po[2,i,t,2] <- 1
##   po[2,i,t,3] <- 0
##   po[2,i,t,4] <- 0
##   po[2,i,t,5] <- 0
##   po[2,i,t,6] <- 0
##   po[2,i,t,7] <- 0
## 
##   po[3,i,t,1] <- 0
##   po[3,i,t,2] <- 0
##   po[3,i,t,3] <- p2[i,t]
##   po[3,i,t,4] <- 0
##   po[3,i,t,5] <- 0
##   po[3,i,t,6] <- 0
##   po[3,i,t,7] <- 1-p2[i,t]
## 
##   po[4,i,t,1] <- 0
##   po[4,i,t,2] <- 0
##   po[4,i,t,3] <- 0
##   po[4,i,t,4] <- 1
##   po[4,i,t,5] <- 0
##   po[4,i,t,6] <- 0
##   po[4,i,t,7] <- 0
## 
##   po[5,i,t,1] <- 0
##   po[5,i,t,2] <- 0
##   po[5,i,t,3] <- 0
##   po[5,i,t,4] <- 0
##   po[5,i,t,5] <- p3[i,t]
##   po[5,i,t,6] <- 0
##   po[5,i,t,7] <- 1-p3[i,t]
## 
##   po[6,i,t,1] <- 0
##   po[6,i,t,2] <- 0
##   po[6,i,t,3] <- 0
##   po[6,i,t,4] <- 0
##   po[6,i,t,5] <- 0
##   po[6,i,t,6] <- 1
##   po[6,i,t,7] <- 0
## 
##   po[7,i,t,1] <- 0
##   po[7,i,t,2] <- 0
##   po[7,i,t,3] <- 0
##   po[7,i,t,4] <- 0
##   po[7,i,t,5] <- 0
##   po[7,i,t,6] <- 0
##   po[7,i,t,7] <- 1
##   } # t
## } # i
## 
##   ## likelihood 
##   for(i in 1:nind){
##     zeta[i,first[i],1]  <- equals(y[i,first[i]],1)
##     zeta[i,first[i],2]  <- 0
##     zeta[i,first[i],3]  <- equals(y[i,first[i]],3)
##     zeta[i,first[i],4]  <- 0
##     zeta[i,first[i],5]  <- equals(y[i,first[i]],5)
##     zeta[i,first[i],6]  <- 0
##     zeta[i,first[i],7]  <- 0
##     for(t in (first[i]+1):last[i]) { 
##       for(j in 1:7){  
##       zeta[i,t,j] <- inprod(zeta[i, t-1,], ps[,i,t-1,j])*po[j,i,t, y[i,t]] 
##       }
##     }
##    lik[i]<- sum(zeta[i,last[i],]) # sum the likelihood over all states (should have one number only)
##    fr[i] ~ dbin(lik[i],fr1[i])  # fr and fr1 are the same but with different names, number of observed capture histories (can be ones only)
##   }
## 
##   ## linear predictors
##   for(i in 1:nind){
##     ## impute unknown sexes
##     sex[i] ~ dcat(propsex)
##     for(v in year[first[i]]:nyears){
##       logit(s1[i,v]) <-  b10[sex[i], age[i,v]] + sigmaS1year*yearS1eff[v] 
##       logit(s2[i,v]) <-  b20[sex[i], age[i,v]] + sigmaS2year*yearS2eff[v] 
##       logit(s3[i,v]) <-  b30
##     }
##     for(t in first[i]:last[i]){
##       # hier multi-variate logit-link funktion, um Veränderung über die Jahre einzufügen
##       m12[i,t] <- m012[sex[i], age[i,year[t]]]
##       m21[i,t] <- m021[sex[i], age[i,year[t]]]
##       m23[i,t] <- m023[sex[i], age[i,year[t]]]
##       m13[i,t] <- m013[sex[i], age[i,year[t]]]
##       logit(p1[i,t]) <- a10[sex[i], age[i,year[t]],  telemetry[i,t]] + a1*monitoringIV[t] + a2*oppeffIV[t] + sigmapind*indeff[i]  #  
##       logit(p2[i,t]) <- a20[sex[i], age[i,year[t]],  telemetry[i,t]] + a1*monitoringIII[t]  + a2*oppeffIII[t] + sigmapind*indeff[i]  # 
##       logit(p3[i,t]) <- a30[sex[i], age[i,year[t]],  telemetry[i,t]]   # 
##      
##       logit(r1[i,t]) <-  d0[sex[i], age[i,year[t]]]
##       logit(r2[i,t]) <-  d0[sex[i], age[i,year[t]]] + d11
##       logit(r3[i,t]) <-  d0[sex[i], age[i,year[t]]] + d12
##     }  
##   }
## 
##   ## priors
##   for(i in 1:nind){
##     indeff[i]~dnorm(0,1)
##   }
##   sigmapind ~ dt(0,1,2)I(0,)
## 
##   for(i in 1:nyears){
##     yearS1eff[i]~dnorm(0,1)
##     yearS2eff[i]~dnorm(0,1)
##   }
##   sigmaS1year ~ dt(0,1,2)I(0,)
##   sigmaS2year ~ dt(0,1,2)I(0,)
## 
##   propsex[1:2] ~ ddirch(alpha[1:2])
## 
## # prior Normal(0,1.5) for intercept in the logit-scale
## # mean corresponds to mean logit(s) for the age and sex class 
##   b10[1,1] ~ dnorm(0, 0.44) # dnorm(-0.75, 0.44) # juvenile females  0.28 in NW, 0.36 in C -> 0.32 = inv_logit(-0.75)
##   b10[2,1] ~ dnorm(0, 0.44) # dnorm(-0.94, 0.44)  # juvenile males  0.29 in NW, 0.27 in C -> 0.28 = inv_logit(-0.94)
##   b10[1,2] ~ dnorm(0, 0.44) # dnorm(1.5, 0.44) # subadult females  0.92 in NW, 0.75 in C -> 0.28 = inv_logit(1.5)
##   b10[2,2] ~ dnorm(0, 0.44) # dnorm(1.18, 0.44)# subadult males  0.75 in NW, 0.78 in C -> 0.765 = inv_logit(1.18)
##   b10[1,3] ~ dnorm(0, 0.44) # dnorm(1.4, 0.44) # adult females  0.76 in NW, 0.85 in C -> 0.805 = inv_logit(1.4)
##   b10[2,3] ~ dnorm(0, 0.44) # dnorm(1.2, 0.44)# adult males  0.76 in NW, 0.78 in C -> 0.77 = inv_logit(1.2)
## 
##   b20[1,1] ~ dnorm(0, 0.44)  # = Normal(0,1.5) für Intercept #dnorm(-0,75, 0.44)
##   b20[2,1] ~ dnorm(0, 0.44)
##   b20[1,2] ~ dnorm(0, 0.44)
##   b20[2,2] ~ dnorm(0, 0.44)
##   b20[1,3] ~ dnorm(0, 0.44)
##   b20[2,3] ~ dnorm(0, 0.44)
## 
##   b30 ~ dnorm(0, 0.044)
##   
##   a10[1,1,1] ~ dnorm(0, 0.44)  # = Normal(0,1.5) für Intercept 
##   a10[2,1,1] ~ dnorm(0, 0.44)
##   a10[1,2,1] ~ dnorm(0, 0.44)
##   a10[2,2,1] ~ dnorm(0, 0.44)
##   a10[1,3,1] ~ dnorm(0, 0.44)
##   a10[2,3,1] ~ dnorm(0, 0.44)
## 
##   a10[1,1,2] ~ dnorm(2.5, 1) # = Normal(0,1.5) für Intercept 
##   a10[2,1,2] <- a10[1,1,2]     # telemetrierte Individuen haben alle die gleichen Entdeckungswahrscheinlichkeit
##   a10[1,2,2] <- a10[1,1,2]
##   a10[2,2,2] <- a10[1,1,2]
##   a10[1,3,2] <- a10[1,1,2]
##   a10[2,3,2] <- a10[1,1,2]
## 
##   a20[1,1,1] ~ dnorm(0, 0.44)  # = Normal(0,1.5) für Intercept 
##   a20[2,1,1] ~ dnorm(0, 0.44)
##   a20[1,2,1] ~ dnorm(0, 0.44)
##   a20[2,2,1] ~ dnorm(0, 0.44)
##   a20[1,3,1] ~ dnorm(0, 0.44)
##   a20[2,3,1] ~ dnorm(0, 0.44)
## 
##   a20[1,1,2] ~ dnorm(2.5, 1)  # = Normal(0,1.5) für Intercept 
##   a20[2,1,2] <- a20[1,1,2]     # telemetrierte Individuen haben alle die gleichen Entdeckungswahrscheinlichkeit
##   a20[1,2,2] <- a20[1,1,2]
##   a20[2,2,2] <- a20[1,1,2]
##   a20[1,3,2] <- a20[1,1,2]
##   a20[2,3,2] <- a20[1,1,2]
## 
##   a30[1,1,1] ~ dnorm(0, 0.44)  # = Normal(0,1.5) für Intercept 
##   a30[2,1,1] ~ dnorm(0, 0.44)
##   a30[1,2,1] ~ dnorm(0, 0.44)
##   a30[2,2,1] ~ dnorm(0, 0.44)
##   a30[1,3,1] ~ dnorm(0, 0.44)
##   a30[2,3,1] ~ dnorm(0, 0.44)
## 
##   a30[1,1,2] ~ dnorm(2.5, 1) # = Normal(0,1.5) für Intercept 
##   a30[2,1,2] <- a30[1,1,2]     # telemetrierte Individuen haben alle die gleichen Entdeckungswahrscheinlichkeit
##   a30[1,2,2] <- a30[1,1,2]
##   a30[2,2,2] <- a30[1,1,2]
##   a30[1,3,2] <- a30[1,1,2]
##   a30[2,3,2] <- a30[1,1,2]
## 
##   a1 ~ dnorm(0, 0.04) 
##   a2 ~ dnorm(0, 0.04) 
##  
##   d0[1,1] ~ dnorm(0, 0.44)
##   d0[2,1] ~ dnorm(0, 0.44)
##   d0[1,2] ~ dnorm(0, 0.44)
##   d0[2,2] ~ dnorm(0, 0.44)
##   d0[1,3] ~ dnorm(0, 0.44)
##   d0[2,3] ~ dnorm(0, 0.44)
## 
##   d11 ~ dnorm(0, 0.04)
##   d12 ~ dnorm(0, 0.04)
## 
##   m012[1,1] ~ dbeta(1,1)
##   m012[2,1] ~ dbeta(1,1)
##   m012[1,2] ~ dbeta(1,1)
##   m012[2,2] ~ dbeta(1,1)
##   m012[1,3] <-   m012[1,2] #~ dbeta(1,1)
##   m012[2,3] <-   m012[2,2] #~ dbeta(1,1)
##  
##   u0[1,1] <- 1- m012[1,1]
##   u0[2,1] <- 1- m012[2,1]
##   u0[1,2] <- 1- m012[1,2]
##   u0[2,2] <- 1- m012[2,2]
##   u0[1,3] <- 1- m012[1,3]
##   u0[2,3] <- 1- m012[2,3]
##   
##   m013[1,1] ~ dunif(0,u0[1,1])
##   m013[2,1] ~ dunif(0,u0[2,1])
##   m013[1,2] ~ dunif(0,u0[1,2])
##   m013[2,2] ~ dunif(0,u0[2,2])
##   m013[1,3] <- m013[1,2] # ~ dunif(0,u0[1,3])
##   m013[2,3] <- m013[2,2] #~ dunif(0,u0[2,3])
## 
##   m021[1,1] ~ dbeta(1,1)
##   m021[2,1] ~ dbeta(1,1)
##   m021[1,2] ~ dbeta(1,1)
##   m021[2,2] ~ dbeta(1,1)
##   m021[1,3] <- m021[1,2] # ~ dbeta(1,1)
##   m021[2,3] <- m021[2,2] # ~ dbeta(1,1)
##  
##   u20[1,1] <- 1- m021[1,1]
##   u20[2,1] <- 1- m021[2,1]
##   u20[1,2] <- 1- m021[1,2]
##   u20[2,2] <- 1- m021[2,2]
##   u20[1,3] <- 1- m021[1,3]
##   u20[2,3] <- 1- m021[2,3]
##   
##   m023[1,1] ~ dunif(0,u20[1,1])
##   m023[2,1] ~ dunif(0,u20[2,1])
##   m023[1,2] ~ dunif(0,u20[1,2])
##   m023[2,2] ~ dunif(0,u20[2,2])
##   m023[1,3] <- m023[1,2] # ~ dunif(0,u20[1,3])
##   m023[2,3] <- m023[2,2] # ~ dunif(0,u20[2,3])
## 
## 
## # model for age at death
##   for(s in 1:2){
##     for(a in 1:3){
##       logit(rsa[s,a,1]) <- d0[s,a] # get recovery probabilty per sex and ageclass 
##       logit(rsa[s,a,2]) <- d0[s,a] + d11 # get recovery probabilty per sex and ageclass 
##       logit(Ssa[s,a, 1]) <- b10[s,a] # get survival per sex, ageclass and area
##       logit(Ssa[s,a, 2]) <- b20[s,a] # get survival per sex, ageclass and area  
##     }
##   # propabiliy of beeing dying within 18 years and beeing found
##  for(aa in 1:2){
##   pfounddead[s, aa]  <- (1-Ssa[s,1,aa])*rsa[s,1,aa] + Ssa[s,1,aa]*(1-Ssa[s,2,aa])*rsa[s,2,aa] + 
##                         Ssa[s,1,aa]*Ssa[s,2,aa]*(1-Ssa[s,3,aa])*rsa[s,3,aa]*(pow(Ssa[s,3, aa],18-2)-1)/(Ssa[s,3, aa]-1)
##    }
##   }
## 
## # likelihood
##   for(i in 1:adnind){
##     yad[i] ~ dcat(adp[i,1:adnyears]) # y[i]: year of dead recovery of individual i
##     
##     adp[i,1] <- (1-adS[i,1])*adr[i, ageclass[1]]/pfounddead[adsex[i], area[i]]
##     adsex[i] ~ dcat(propsex)
##     for(j in 2:adnyears){ 
##       adp[i,j] <- prod(adS[i,1:(j-1)])*(1-adS[i,j])*adr[i, ageclass[j]]/pfounddead[adsex[i], area[i]]
##     }
##     for(j in 1:adnyears){
##       adS[i,j] <- Ssa[adsex[i],ageclass[j], area[i]] # same intercept as in multi-state model
##       adr[i,j] <- rsa[adsex[i],ageclass[j], area[i]]  
##     }
##   }
## }
```

```
# with interaction age x year for survival
mod <- jags(datax, inits=initfun, parameters.to.save=c("a10", "a20",  "a30", "a1", "b10", "b20","b30",  "d0", "m012", "m021", "m023", "m013", "sigmapind", "indeff", "sigmaSyear", "yearSeff", "propsex", "sex"),
            model.file="jags/Smod_alps_btocombined_ageyearint_ragedep_marginalisation.txt", n.chains=3, n.iter=30000, n.thin=5)


mod <- mod$BUGSoutput
save(mod, file="modelfits/modelfit_alps_combined_ageyear241223.rda") # save the model
```

## 3.3 Results

```
load("modelfits/modelfit_alps_combined_ageyear241205.rda") # m per sex and age

# assess convergence
tab <- mod$summary[c(1:60, 601:626),]
kable(tab, dig=2, caption="Statistics of the model estimates including convergence assessment statistics. For interpretation of the parameters, see legend to table 1.")
```

Table 3.1: Statistics of the model estimates including convergence assessment statistics. For interpretation of the parameters, see legend to table 1.


|  | mean | sd | 2.5% | 25% | 50% | 75% | 97.5% | Rhat | n.eff |
| --- | --- | --- | --- | --- | --- | --- | --- | --- | --- |
| a1 | 0.45 | 0.03 | 0.40 | 0.43 | 0.45 | 0.46 | 0.50 | 1.00 | 6900 |
| a10[1,1,1] | -0.41 | 0.29 | -0.98 | -0.60 | -0.41 | -0.22 | 0.14 | 1.00 | 8800 |
| a10[2,1,1] | -1.39 | 0.33 | -2.07 | -1.61 | -1.39 | -1.17 | -0.75 | 1.00 | 5400 |
| a10[1,2,1] | -2.12 | 0.24 | -2.60 | -2.29 | -2.12 | -1.96 | -1.67 | 1.00 | 10000 |
| a10[2,2,1] | -1.67 | 0.23 | -2.13 | -1.82 | -1.67 | -1.51 | -1.24 | 1.00 | 7300 |
| a10[1,3,1] | -1.96 | 0.13 | -2.20 | -2.04 | -1.96 | -1.87 | -1.71 | 1.00 | 1600 |
| a10[2,3,1] | -1.43 | 0.12 | -1.66 | -1.51 | -1.43 | -1.35 | -1.20 | 1.00 | 1100 |
| a10[1,1,2] | 2.92 | 0.20 | 2.53 | 2.78 | 2.91 | 3.05 | 3.34 | 1.00 | 1800 |
| a10[2,1,2] | 2.92 | 0.20 | 2.53 | 2.78 | 2.91 | 3.05 | 3.34 | 1.00 | 1800 |
| a10[1,2,2] | 2.92 | 0.20 | 2.53 | 2.78 | 2.91 | 3.05 | 3.34 | 1.00 | 1800 |
| a10[2,2,2] | 2.92 | 0.20 | 2.53 | 2.78 | 2.91 | 3.05 | 3.34 | 1.00 | 1800 |
| a10[1,3,2] | 2.92 | 0.20 | 2.53 | 2.78 | 2.91 | 3.05 | 3.34 | 1.00 | 1800 |
| a10[2,3,2] | 2.92 | 0.20 | 2.53 | 2.78 | 2.91 | 3.05 | 3.34 | 1.00 | 1800 |
| a2 | 0.00 | 0.06 | -0.11 | -0.04 | 0.00 | 0.04 | 0.12 | 1.00 | 1500 |
| a20[1,1,1] | -1.10 | 0.59 | -2.28 | -1.48 | -1.11 | -0.71 | 0.07 | 1.00 | 10000 |
| a20[2,1,1] | -0.76 | 0.63 | -2.01 | -1.17 | -0.77 | -0.35 | 0.47 | 1.00 | 5400 |
| a20[1,2,1] | -2.37 | 0.50 | -3.42 | -2.69 | -2.35 | -2.03 | -1.45 | 1.00 | 5100 |
| a20[2,2,1] | -1.85 | 0.47 | -2.80 | -2.14 | -1.83 | -1.53 | -0.95 | 1.00 | 9700 |
| a20[1,3,1] | -2.04 | 0.22 | -2.49 | -2.19 | -2.04 | -1.90 | -1.61 | 1.00 | 1400 |
| a20[2,3,1] | -1.79 | 0.22 | -2.23 | -1.94 | -1.79 | -1.64 | -1.36 | 1.00 | 1200 |
| a20[1,1,2] | 3.26 | 0.78 | 1.82 | 2.72 | 3.23 | 3.77 | 4.91 | 1.00 | 10000 |
| a20[2,1,2] | 3.26 | 0.78 | 1.82 | 2.72 | 3.23 | 3.77 | 4.91 | 1.00 | 10000 |
| a20[1,2,2] | 3.26 | 0.78 | 1.82 | 2.72 | 3.23 | 3.77 | 4.91 | 1.00 | 10000 |
| a20[2,2,2] | 3.26 | 0.78 | 1.82 | 2.72 | 3.23 | 3.77 | 4.91 | 1.00 | 10000 |
| a20[1,3,2] | 3.26 | 0.78 | 1.82 | 2.72 | 3.23 | 3.77 | 4.91 | 1.00 | 10000 |
| a20[2,3,2] | 3.26 | 0.78 | 1.82 | 2.72 | 3.23 | 3.77 | 4.91 | 1.00 | 10000 |
| a30[1,1,1] | 0.22 | 1.70 | -3.20 | -0.98 | 0.35 | 1.42 | 3.34 | 1.00 | 3000 |
| a30[2,1,1] | 0.11 | 1.67 | -3.22 | -1.06 | 0.20 | 1.29 | 3.21 | 1.00 | 1400 |
| a30[1,2,1] | -0.47 | 1.56 | -3.26 | -1.57 | -0.57 | 0.57 | 2.72 | 1.00 | 10000 |
| a30[2,2,1] | -1.26 | 1.02 | -2.99 | -1.90 | -1.36 | -0.77 | 1.29 | 1.00 | 7600 |
| a30[1,3,1] | -2.48 | 1.10 | -4.99 | -3.10 | -2.28 | -1.75 | -0.80 | 1.00 | 1500 |
| a30[2,3,1] | -0.82 | 0.40 | -1.56 | -1.11 | -0.84 | -0.55 | -0.02 | 1.00 | 10000 |
| a30[1,1,2] | 2.50 | 0.99 | 0.57 | 1.81 | 2.49 | 3.17 | 4.44 | 1.00 | 10000 |
| a30[2,1,2] | 2.50 | 0.99 | 0.57 | 1.81 | 2.49 | 3.17 | 4.44 | 1.00 | 10000 |
| a30[1,2,2] | 2.50 | 0.99 | 0.57 | 1.81 | 2.49 | 3.17 | 4.44 | 1.00 | 10000 |
| a30[2,2,2] | 2.50 | 0.99 | 0.57 | 1.81 | 2.49 | 3.17 | 4.44 | 1.00 | 10000 |
| a30[1,3,2] | 2.50 | 0.99 | 0.57 | 1.81 | 2.49 | 3.17 | 4.44 | 1.00 | 10000 |
| a30[2,3,2] | 2.50 | 0.99 | 0.57 | 1.81 | 2.49 | 3.17 | 4.44 | 1.00 | 10000 |
| b10[1,1] | -0.73 | 0.47 | -1.60 | -1.05 | -0.75 | -0.43 | 0.26 | 1.01 | 680 |
| b10[2,1] | -0.90 | 0.44 | -1.75 | -1.20 | -0.92 | -0.63 | 0.03 | 1.00 | 1300 |
| b10[1,2] | 1.50 | 0.61 | 0.45 | 1.08 | 1.45 | 1.88 | 2.83 | 1.00 | 1000 |
| b10[2,2] | 1.69 | 0.54 | 0.66 | 1.32 | 1.69 | 2.06 | 2.76 | 1.01 | 670 |
| b10[1,3] | 1.29 | 0.15 | 1.02 | 1.19 | 1.28 | 1.38 | 1.60 | 1.00 | 2200 |
| b10[2,3] | 1.28 | 0.13 | 1.04 | 1.20 | 1.28 | 1.37 | 1.55 | 1.00 | 2700 |
| b20[1,1] | -0.94 | 0.60 | -2.07 | -1.35 | -0.96 | -0.56 | 0.28 | 1.00 | 1300 |
| b20[2,1] | -1.40 | 0.66 | -2.70 | -1.82 | -1.40 | -0.96 | -0.09 | 1.00 | 1400 |
| b20[1,2] | 2.05 | 0.97 | 0.34 | 1.37 | 1.99 | 2.64 | 4.13 | 1.00 | 2500 |
| b20[2,2] | 1.22 | 0.79 | -0.23 | 0.66 | 1.17 | 1.72 | 2.89 | 1.00 | 1500 |
| b20[1,3] | 1.29 | 0.32 | 0.73 | 1.08 | 1.27 | 1.49 | 1.99 | 1.00 | 3800 |
| b20[2,3] | 0.89 | 0.30 | 0.32 | 0.69 | 0.88 | 1.09 | 1.50 | 1.00 | 4000 |
| b30 | 0.18 | 0.53 | -0.84 | -0.17 | 0.17 | 0.52 | 1.24 | 1.00 | 7300 |
| d0[1,1] | -2.12 | 0.50 | -3.04 | -2.46 | -2.14 | -1.82 | -1.11 | 1.00 | 720 |
| d0[2,1] | -2.53 | 0.47 | -3.44 | -2.83 | -2.53 | -2.22 | -1.58 | 1.00 | 3200 |
| d0[1,2] | -1.93 | 0.78 | -3.30 | -2.46 | -1.97 | -1.47 | -0.18 | 1.00 | 1200 |
| d0[2,2] | -0.72 | 0.84 | -2.12 | -1.28 | -0.81 | -0.24 | 1.20 | 1.01 | 490 |
| d0[1,3] | -1.87 | 0.25 | -2.35 | -2.03 | -1.86 | -1.70 | -1.39 | 1.00 | 10000 |
| d0[2,3] | -1.74 | 0.23 | -2.20 | -1.90 | -1.73 | -1.58 | -1.29 | 1.00 | 4000 |
| d11 | -1.56 | 0.56 | -2.78 | -1.91 | -1.53 | -1.17 | -0.57 | 1.00 | 10000 |
| d12 | -4.94 | 2.97 | -11.89 | -6.66 | -4.48 | -2.71 | -0.57 | 1.00 | 3500 |
| deviance | 9984.90 | 30.01 | 9927.56 | 9964.43 | 9984.71 | 10005.15 | 10045.04 | 1.00 | 1900 |
| m012[1,1] | 0.01 | 0.01 | 0.00 | 0.00 | 0.01 | 0.01 | 0.04 | 1.00 | 8600 |
| m012[2,1] | 0.02 | 0.02 | 0.00 | 0.01 | 0.02 | 0.03 | 0.07 | 1.00 | 4200 |
| m012[1,2] | 0.00 | 0.00 | 0.00 | 0.00 | 0.00 | 0.00 | 0.00 | 1.00 | 2500 |
| m012[2,2] | 0.00 | 0.00 | 0.00 | 0.00 | 0.00 | 0.01 | 0.01 | 1.00 | 5500 |
| m012[1,3] | 0.00 | 0.00 | 0.00 | 0.00 | 0.00 | 0.00 | 0.00 | 1.00 | 2500 |
| m012[2,3] | 0.00 | 0.00 | 0.00 | 0.00 | 0.00 | 0.01 | 0.01 | 1.00 | 5500 |
| m013[1,1] | 0.01 | 0.01 | 0.00 | 0.00 | 0.01 | 0.02 | 0.04 | 1.00 | 4200 |
| m013[2,1] | 0.01 | 0.01 | 0.00 | 0.00 | 0.01 | 0.02 | 0.05 | 1.00 | 10000 |
| m013[1,2] | 0.00 | 0.00 | 0.00 | 0.00 | 0.00 | 0.00 | 0.01 | 1.00 | 1100 |
| m013[2,2] | 0.00 | 0.00 | 0.00 | 0.00 | 0.00 | 0.00 | 0.00 | 1.00 | 8500 |
| m013[1,3] | 0.00 | 0.00 | 0.00 | 0.00 | 0.00 | 0.00 | 0.01 | 1.00 | 1100 |
| m013[2,3] | 0.00 | 0.00 | 0.00 | 0.00 | 0.00 | 0.00 | 0.00 | 1.00 | 8500 |
| m021[1,1] | 0.08 | 0.06 | 0.01 | 0.04 | 0.07 | 0.12 | 0.22 | 1.00 | 8300 |
| m021[2,1] | 0.06 | 0.05 | 0.00 | 0.02 | 0.05 | 0.09 | 0.19 | 1.00 | 10000 |
| m021[1,2] | 0.01 | 0.00 | 0.00 | 0.00 | 0.01 | 0.01 | 0.02 | 1.00 | 4800 |
| m021[2,2] | 0.01 | 0.00 | 0.00 | 0.01 | 0.01 | 0.01 | 0.02 | 1.00 | 10000 |
| m021[1,3] | 0.01 | 0.00 | 0.00 | 0.00 | 0.01 | 0.01 | 0.02 | 1.00 | 4800 |
| m021[2,3] | 0.01 | 0.00 | 0.00 | 0.01 | 0.01 | 0.01 | 0.02 | 1.00 | 10000 |
| m023[1,1] | 0.04 | 0.04 | 0.00 | 0.02 | 0.03 | 0.06 | 0.15 | 1.00 | 1900 |
| m023[2,1] | 0.07 | 0.05 | 0.01 | 0.03 | 0.06 | 0.10 | 0.21 | 1.00 | 2600 |
| m023[1,2] | 0.01 | 0.01 | 0.00 | 0.00 | 0.00 | 0.01 | 0.02 | 1.00 | 3600 |
| m023[2,2] | 0.00 | 0.00 | 0.00 | 0.00 | 0.00 | 0.00 | 0.01 | 1.00 | 10000 |
| m023[1,3] | 0.01 | 0.01 | 0.00 | 0.00 | 0.00 | 0.01 | 0.02 | 1.00 | 3600 |
| m023[2,3] | 0.00 | 0.00 | 0.00 | 0.00 | 0.00 | 0.00 | 0.01 | 1.00 | 10000 |
| propsex[1] | 0.49 | 0.02 | 0.45 | 0.48 | 0.49 | 0.51 | 0.54 | 1.00 | 10000 |
| propsex[2] | 0.51 | 0.02 | 0.46 | 0.49 | 0.51 | 0.52 | 0.55 | 1.00 | 10000 |

```
# check convergence
 # plot(mod$sims.array[,1,1], type="l")
 # lines(mod$sims.array[,2,1], col=2)
 # lines(mod$sims.array[,3,1], col=3)

  # plot(mod$sims.array[,1,10], type="l")
  # lines(mod$sims.array[,2,10], col=2)
  # lines(mod$sims.array[,3,10], col=3)
```

```
S1 <- plogis(apply(mod$sims.list$b10, c(2,3), mean))
S1lwr <- plogis(apply(mod$sims.list$b10, c(2,3), quantile, probs=0.025))
S1upr <- plogis(apply(mod$sims.list$b10, c(2,3), quantile, probs=0.975))
 
S2 <- plogis(apply(mod$sims.list$b20, c(2,3), mean))
S2lwr <- plogis(apply(mod$sims.list$b20, c(2,3), quantile, probs=0.025))
S2upr <- plogis(apply(mod$sims.list$b20, c(2,3), quantile, probs=0.975))

  
plot(1:3, seq(0,1, length=3), type="n", las=1, xaxt="n", xlab="", ylim=c(0,1), ylab="Annual survival", xlim=c(0.5, 3.5))
#abline(h=0.5, col=grey(0.8), lwd=2) # prior mean
# females
segments(c(1:3)+0.1, S1lwr[1,], c(1:3)+0.1, S1upr[1,],lwd=2, lend="butt", col="orange")
points(c(1:3)+0.1, S1[1,], pch=21, col="orange", bg="white")
# males
segments(c(1:3)-0.1, S1lwr[2,], c(1:3)-0.1, S1upr[2,],lwd=2, lend="butt", col="blue")
points(c(1:3)-0.1, S1[2,], pch=21, col="blue", bg="white")


segments(c(1:3)+0.19, S2lwr[1,], c(1:3)+0.19, S2upr[1,],lwd=2, lend="butt", col="orange")
points(c(1:3)+0.19, S2[1,], pch=16, col="orange")
# males
segments(c(1:3)-0.01, S2lwr[2,], c(1:3)-0.01, S2upr[2,],lwd=2, lend="butt", col="blue")
points(c(1:3)-0.01, S2[2,], pch=16, col="blue")


axis(1, at=1:3, labels=c("juvenile", "subadult", "adult"))
legend(0.5, 1.25, xpd=NA, lwd=2, col=c("orange", "blue", "orange", "blue"),
       pch=c(21,21,16,16), pt.bg="white", legend=c("females NSA", "males NSA", "females CS", "males CS"), ncol=2, bty="n")
```

Figure 3.1: Estimated average annual apparent survival probability per age class based on combined data. Vertical bars are 95% compatibility intervals.

```
tab <- expand.grid(age=c("First year", "Second year", "Adult"), 
                   sex=c("females", "males"))
tab$VDWAlps_S <- as.numeric(t(S1))
tab$VDWAlps_S.lwr <- as.numeric(t(S1lwr))
tab$VDWAlps_S.upr <- as.numeric(t(S1upr))
tab$CAlps_S <- as.numeric(t(S2))
tab$CAlps_S.lwr <- as.numeric(t(S2lwr))
tab$CAlps_S.upr <- as.numeric(t(S2upr))
kable(tab, dig=2, caption="Average annual survival estimates from the combined model with 95% uncertainty interval.")
```

Table 3.2: Average annual survival estimates from the combined model with 95% uncertainty interval.


| age | sex | VDWAlps\_S | VDWAlps\_S.lwr | VDWAlps\_S.upr | CAlps\_S | CAlps\_S.lwr | CAlps\_S.upr |
| --- | --- | --- | --- | --- | --- | --- | --- |
| First year | females | 0.33 | 0.17 | 0.57 | 0.28 | 0.11 | 0.57 |
| Second year | females | 0.82 | 0.61 | 0.94 | 0.89 | 0.58 | 0.98 |
| Adult | females | 0.78 | 0.74 | 0.83 | 0.78 | 0.67 | 0.88 |
| First year | males | 0.29 | 0.15 | 0.51 | 0.20 | 0.06 | 0.48 |
| Second year | males | 0.84 | 0.66 | 0.94 | 0.77 | 0.44 | 0.95 |
| Adult | males | 0.78 | 0.74 | 0.82 | 0.71 | 0.58 | 0.82 |

```
nsim <- mod$n.sims

S1pyear <- array(NA, dim=c(2,3, datax$nyears, mod$n.sims))
for(i in 1:2){
  for(j in 1:3){
    S1pyear[i,j,,] <- matrix(mod$sims.list$b10[,i,j], ncol=mod$n.sims, nrow=datax$nyears, byrow=TRUE)+ matrix(mod$sims.list$sigmaS1year, ncol=mod$n.sims, nrow=datax$nyears, byrow=TRUE)*t(mod$sims.list$yearS1eff[,1:datax$nyears])
  }
}


S2pyear <- array(NA, dim=c(2,3, datax$nyears, mod$n.sims))
for(i in 1:2){
  for(j in 1:3){
    S2pyear[i,j,,] <- matrix(mod$sims.list$b20[,i,j], ncol=mod$n.sims, nrow=datax$nyears, byrow=TRUE)+ matrix(mod$sims.list$sigmaS2year, ncol=mod$n.sims, nrow=datax$nyears, byrow=TRUE)*t(mod$sims.list$yearS2eff[,1:datax$nyears])
  }
}

S1pyearm <- plogis(apply(S1pyear, c(1,2,3), mean))
S1pyearlwr <- plogis(apply(S1pyear, c(1,2,3), quantile, probs=0.025))
S1pyearupr <- plogis(apply(S1pyear, c(1,2,3), quantile, probs=0.975))

S2pyearm <- plogis(apply(S2pyear, c(1,2,3), mean))
S2pyearlwr <- plogis(apply(S2pyear, c(1,2,3), quantile, probs=0.025))
S2pyearupr <- plogis(apply(S2pyear, c(1,2,3), quantile, probs=0.975))


par(mfrow=c(3,1), mar=c(0.5, 3,1.5, 0.5), oma=c(3,3,0.6,0))
plot(1:datax$nyears, seq(0,1, length=datax$nyears), type="n", las=1, xaxt="n", xlab="", ylim=c(0,1), ylab="Annual survival", xlim=c(1,datax$nyears))
mtext("a)", adj=0, side=3) # adults


legend(14, 1.26, xpd=NA, col=c("orange", "blue", "brown", "lightblue"), pch=c(21,21,16,16), bg="white", legend=c("females NSA", "males NSA", "females CS", "males CS"), ncol=2, bty="n")


segments(c(1:datax$nyears), S1pyearlwr[1,3,], c(1:datax$nyears), S1pyearupr[1,3,],lwd=2, lend="butt", col="orange")
points(c(1:datax$nyears), S1pyearm[1,3,], pch=21, col="orange", bg="white")

segments(c(1:datax$nyears)-0.15, S1pyearlwr[2,3,], c(1:datax$nyears)-0.15, S1pyearupr[2,3,],lwd=2, lend="butt", col="blue")
points(c(1:datax$nyears)-0.15, S1pyearm[2,3,], pch=21, col="blue", bg="white")


segments(c(1:datax$nyears)+0.2, S2pyearlwr[1,3,], c(1:datax$nyears)+0.2, S2pyearupr[1,3,],lwd=2, lend="butt", col="brown")
points(c(1:datax$nyears)+0.2, S2pyearm[1,3,], pch=16, col="brown")

segments(c(1:datax$nyears)+0.3, S2pyearlwr[2,3,], c(1:datax$nyears)+0.3, S2pyearupr[2,3,],lwd=2, lend="butt", col="lightblue")
points(c(1:datax$nyears)+0.3, S2pyearm[2,3,], pch=16, col="lightblue")

plot(1:datax$nyears, seq(0,1, length=datax$nyears), type="n", las=1, xaxt="n", xlab="", ylim=c(0,1), ylab="Annual survival", xlim=c(1,datax$nyears))

segments(c(1:datax$nyears), S1pyearlwr[1,2,], c(1:datax$nyears), S1pyearupr[1,2,],lwd=2, lend="butt", col="orange")
points(c(1:datax$nyears), S1pyearm[1,2,], pch=21, col="orange", bg="white")

segments(c(1:datax$nyears)-0.15, S1pyearlwr[2,2,], c(1:datax$nyears)-0.15, S1pyearupr[2,2,],lwd=2, lend="butt", col="blue")
points(c(1:datax$nyears)-0.15, S1pyearm[2,2,], pch=21, col="blue", bg="white")


segments(c(1:datax$nyears)+0.2, S2pyearlwr[1,2,], c(1:datax$nyears)+0.2, S2pyearupr[1,2,],lwd=2, lend="butt", col="brown")
points(c(1:datax$nyears)+0.2, S2pyearm[1,2,], pch=16, col="brown")

segments(c(1:datax$nyears)+0.35, S2pyearlwr[2,2,], c(1:datax$nyears)+0.35, S2pyearupr[2,2,],lwd=2, lend="butt", col="lightblue")
points(c(1:datax$nyears)+0.35, S2pyearm[2,2,], pch=16, col="lightblue")
mtext("b)", adj=0, side=3) # second year


plot(1:datax$nyears, seq(0,1, length=datax$nyears), type="n", las=1, xaxt="n", xlab="", ylim=c(0,1), ylab="Annual survival", xlim=c(1,datax$nyears))

segments(c(1:datax$nyears), S1pyearlwr[1,1,], c(1:datax$nyears), S1pyearupr[1,1,],lwd=2, lend="butt", col="orange")
points(c(1:datax$nyears), S1pyearm[1,1,], pch=21, col="orange", bg="white")

segments(c(1:datax$nyears)-0.15, S1pyearlwr[2,1,], c(1:datax$nyears)-0.15, S1pyearupr[2,1,],lwd=2, lend="butt", col="blue")
points(c(1:datax$nyears)-0.15, S1pyearm[2,1,], pch=21, col="blue", bg="white")


segments(c(1:datax$nyears)+0.2, S2pyearlwr[1,1,], c(1:datax$nyears)+0.2, S2pyearupr[1,1,],lwd=2, lend="butt", col="brown")
points(c(1:datax$nyears)+0.2, S2pyearm[1,1,], pch=16, col="brown")

segments(c(1:datax$nyears)+0.35, S2pyearlwr[2,1,], c(1:datax$nyears)+0.35, S2pyearupr[2,1,],lwd=2, lend="butt", col="lightblue")
points(c(1:datax$nyears)+0.35, S2pyearm[2,1,], pch=16, col="lightblue")

mtext("c)", adj=0, side=3) # first year
mtext("Annual survival", side=2, outer=TRUE, line=0.5)

axis(1, at=1:datax$nyears, labels=1997:c(1997+(datax$nyears-1)))
```

Figure 3.2: Estimated annual apparent survival probability for adults based on combined data. Vertical bars are 95% compatibility intervals. Orange=females, blue=males.

```
# Among-year SD in VD-W-Alps
quantile(mod$sims.list$sigmaS1year, probs=c(0.025, 0.5, 0.975))
```

```
##       2.5%        50%      97.5% 
## 0.03224955 0.35850455 0.74534497
```

```
# Among-year SD in C-Alps
quantile(mod$sims.list$sigmaS2year, probs=c(0.025, 0.5, 0.975))
```

```
##       2.5%        50%      97.5% 
## 0.09712499 0.71078827 1.54780234
```

```
# mean age equals -1/ln(S) if S is constant with age (exponential distribution)
# that is not the case -> Monte Carlo simulation to estimate mean age

nsim <- mod$n.sims
virtpop <- array(dim=c(nsim, 100, 2,2)) # max 100 years
virtpop[,1,,] <- 1 # all individuals are once born
for(i in 2:100){
  if(i==2){ 
    virtpop[,i,1,1] <- rbinom(nsim, size=virtpop[,i-1,1,1], prob=plogis(mod$sims.list$b10[,1,1]))
    virtpop[,i,2,1] <- rbinom(nsim, size=virtpop[,i-1,2,1], prob=plogis(mod$sims.list$b10[,2,1]))
        virtpop[,i,1,2] <- rbinom(nsim, size=virtpop[,i-1,1,2], prob=plogis(mod$sims.list$b20[,1,1]))
    virtpop[,i,2,2] <- rbinom(nsim, size=virtpop[,i-1,2,2], prob=plogis(mod$sims.list$b20[,2,1]))
  }
  if(i==3){ 
    virtpop[,i,1,1] <- rbinom(nsim, size=virtpop[,i-1,1,1], prob=plogis(mod$sims.list$b10[,1,2]))
    virtpop[,i,2,1] <- rbinom(nsim, size=virtpop[,i-1,2,1], prob=plogis(mod$sims.list$b10[,2,2]))
        virtpop[,i,1,2] <- rbinom(nsim, size=virtpop[,i-1,1,2], prob=plogis(mod$sims.list$b20[,1,2]))
    virtpop[,i,2,2] <- rbinom(nsim, size=virtpop[,i-1,2,2], prob=plogis(mod$sims.list$b20[,2,2]))
  }
  if(i>3){ 
   virtpop[,i,1,1] <- rbinom(nsim, size=virtpop[,i-1,1,1], prob=plogis(mod$sims.list$b10[,1,3]))
    virtpop[,i,2,1] <- rbinom(nsim, size=virtpop[,i-1,2,1], prob=plogis(mod$sims.list$b10[,2,3]))
        virtpop[,i,1,2] <- rbinom(nsim, size=virtpop[,i-1,1,2], prob=plogis(mod$sims.list$b20[,1,3]))
    virtpop[,i,2,2] <- rbinom(nsim, size=virtpop[,i-1,2,2], prob=plogis(mod$sims.list$b20[,2,3]))
  }
}

SurvF1 <- apply(virtpop[,,1,1], 2, sum)/nsim
SurvM1 <- apply(virtpop[,,2,1], 2, sum)/nsim
SurvF2 <- apply(virtpop[,,1,2], 2, sum)/nsim
SurvM2 <- apply(virtpop[,,2,2], 2, sum)/nsim

plot(0:99, SurvF1, type="l", lwd=2, col="orange", xlim=c(0,12), las=1, ylab="Proportion survived", xlab="Age [years]")
lines(0:99, SurvM1, lwd=2, col="blue")
lines(0:99, SurvF2, lwd=2, col="orange", lty=3)
lines(0:99, SurvM2, lwd=2, col="blue", lty=3)
legend(5,1, lwd=2, lty=c(1,1,3,3), col=c("orange", "blue", "orange", "blue"),
       legend=c("female NSA", "males NSA", "female CS", "male CS"))
```

Figure 3.3: Survivor curve for the two areas per sex

```
# average age
agefun <- function(x) max(c(1:100)[x==1])-0.5
mean(apply(virtpop[,,1,1], 1, agefun)) # females VD-W
```

```
## [1] 2.0584
```

```
mean(apply(virtpop[,,2,1], 1, agefun)) # males VD-W
```

```
## [1] 1.8991
```

```
mean(apply(virtpop[,,1,2], 1, agefun)) # females C
```

```
## [1] 2.014
```

```
mean(apply(virtpop[,,2,2], 1, agefun)) # males C
```

```
## [1] 1.2841
```

```
# dimensions:
# 1: females, males
# 2: age classes
# 3: no telemtry vs. telemetry
tab <- data.frame(sex=c("females", "females", "females", "males", "males", "males", "telemetry"),
                  age=c(1,2,3,1,2,3,1))

tab$p_VDWAlps <- plogis(c(apply(mod$sims.list$a10[,1,,1], 2, mean),
                      apply(mod$sims.list$a10[,2,,1], 2, mean), mean(mod$sims.list$a10[,1,1,2])))

tab$p_VDWAlps.lwr <- plogis(c(apply(mod$sims.list$a10[,1,,1], 2, quantile, probs=0.025),
                      apply(mod$sims.list$a10[,2,,1], 2, quantile, probs=0.025), quantile(mod$sims.list$a10[,1,1,2], probs=0.025)))
tab$p_VDWAlps.upr <- plogis(c(apply(mod$sims.list$a10[,1,,1], 2, quantile, probs=0.975),
                      apply(mod$sims.list$a10[,2,,1], 2, quantile, probs=0.975), quantile(mod$sims.list$a10[,1,1,2], probs=0.975)))


tab$p_CAlps <- plogis(c(apply(mod$sims.list$a20[,1,,1], 2, mean),
                      apply(mod$sims.list$a20[,2,,1], 2, mean), mean(mod$sims.list$a20[,1,1,2])))

tab$p_CAlps.lwr <- plogis(c(apply(mod$sims.list$a20[,1,,1], 2, quantile, probs=0.025),
                      apply(mod$sims.list$a20[,2,,1], 2, quantile, probs=0.025), quantile(mod$sims.list$a20[,1,1,2], probs=0.025)))
tab$p_CAlps.upr <- plogis(c(apply(mod$sims.list$a20[,1,,1], 2, quantile, probs=0.975),
                      apply(mod$sims.list$a20[,2,,1], 2, quantile, probs=0.975), quantile(mod$sims.list$a20[,1,1,2], probs=0.975)))

kable(tab, dig=2, caption="Probabilities to get pictured within one 2-month period for the different sexes and age classes and if tagged by telemetry in the two areas.")
```

Table 3.3: Probabilities to get pictured within one 2-month period for the different sexes and age classes and if tagged by telemetry in the two areas.


| sex | age | p\_VDWAlps | p\_VDWAlps.lwr | p\_VDWAlps.upr | p\_CAlps | p\_CAlps.lwr | p\_CAlps.upr |
| --- | --- | --- | --- | --- | --- | --- | --- |
| females | 1 | 0.40 | 0.27 | 0.54 | 0.25 | 0.09 | 0.52 |
| females | 2 | 0.11 | 0.07 | 0.16 | 0.09 | 0.03 | 0.19 |
| females | 3 | 0.12 | 0.10 | 0.15 | 0.11 | 0.08 | 0.17 |
| males | 1 | 0.20 | 0.11 | 0.32 | 0.32 | 0.12 | 0.62 |
| males | 2 | 0.16 | 0.11 | 0.22 | 0.14 | 0.06 | 0.28 |
| males | 3 | 0.19 | 0.16 | 0.23 | 0.14 | 0.10 | 0.20 |
| telemetry | 1 | 0.95 | 0.93 | 0.97 | 0.96 | 0.86 | 0.99 |

```
quantile(mod$sims.list$a1, probs=c(0.5, 0.025, 0.975))
```

```
##       50%      2.5%     97.5% 
## 0.4463446 0.3952201 0.4991249
```

```
quantile(mod$sims.list$a2, probs=c(0.5, 0.025, 0.975))
```

```
##          50%         2.5%        97.5% 
##  0.004544796 -0.108407377  0.115573827
```

```
tab <- data.frame(sex=c("females", "females", "females", "males", "males", "males"),
                  age=c(1,2,3,1,2,3))

tab$r <- plogis(c(apply(mod$sims.list$d0[,1,], 2, mean),
                      apply(mod$sims.list$d0[,2,], 2, mean)))
tab$r.lwr <- plogis(c(apply(mod$sims.list$d0[,1,], 2, quantile, prob=0.025),
                      apply(mod$sims.list$d0[,2,], 2, quantile, prob=0.025)))
tab$r.upr <- plogis(c(apply(mod$sims.list$d0[,1,], 2, quantile, prob=0.975),
                      apply(mod$sims.list$d0[,2,], 2, quantile, prob=0.975)))

kable(tab, dig=2, caption="Probability that a dead lynx is found from the model fitted to the combined data including age at death data.")
```

Table 3.4: Probability that a dead lynx is found from the model fitted to the combined data including age at death data.

| sex | age | r | r.lwr | r.upr |
| --- | --- | --- | --- | --- |
| females | 1 | 0.11 | 0.05 | 0.25 |
| females | 2 | 0.13 | 0.04 | 0.45 |
| females | 3 | 0.13 | 0.09 | 0.20 |
| males | 1 | 0.07 | 0.03 | 0.17 |
| males | 2 | 0.33 | 0.11 | 0.77 |
| males | 3 | 0.15 | 0.10 | 0.22 |

```
tab <- data.frame(sex=c("female", "female", "female", "male", "male","male"),
                  age=c(1,2,3,1,2,3))
tab$m12 <- c(apply(mod$sims.list$m012[,1,], 2, mean), apply(mod$sims.list$m012[,2,], 2, mean))
tab$m12.l <- c(apply(mod$sims.list$m012[,1,], 2, quantile, probs=0.025), apply(mod$sims.list$m012[,2,], 2, quantile, probs=0.025))
tab$m12.u <- c(apply(mod$sims.list$m012[,1,], 2, quantile, probs=0.975), apply(mod$sims.list$m012[,2,], 2, quantile, probs=0.975))

tab$m21 <- c(apply(mod$sims.list$m021[,1,], 2, mean), apply(mod$sims.list$m021[,2,], 2, mean))
tab$m21.l <- c(apply(mod$sims.list$m021[,1,], 2, quantile, probs=0.025), apply(mod$sims.list$m021[,2,], 2, quantile, probs=0.025))
tab$m21.u <- c(apply(mod$sims.list$m012[,1,], 2, quantile, probs=0.975), apply(mod$sims.list$m021[,2,], 2, quantile, probs=0.975))

tab$m13 <- c(apply(mod$sims.list$m013[,1,], 2, mean), apply(mod$sims.list$m013[,2,], 2, mean))
tab$m13.l <- c(apply(mod$sims.list$m013[,1,], 2, quantile, probs=0.025), apply(mod$sims.list$m013[,2,], 2, quantile, probs=0.025))
tab$m13.u <- c(apply(mod$sims.list$m013[,1,], 2, quantile, probs=0.975), apply(mod$sims.list$m013[,2,], 2, quantile, probs=0.975))

tab$m23 <- c(apply(mod$sims.list$m023[,1,], 2, mean), apply(mod$sims.list$m023[,2,], 2, mean))
tab$m23.l <- c(apply(mod$sims.list$m023[,1,], 2, quantile, probs=0.025), apply(mod$sims.list$m023[,2,], 2, quantile, probs=0.025))
tab$m13.u <- c(apply(mod$sims.list$m023[,1,], 2, quantile, probs=0.975), apply(mod$sims.list$m023[,2,], 2, quantile, probs=0.975))

kable(tab, dig=2, caption="Probabilities to move between the areas 1 (VD-W-Alps), 2 (C-Alps) and 3 (outside) for each age and sex class.")
```

Table 3.5: Probabilities to move between the areas 1 (VD-W-Alps), 2 (C-Alps) and 3 (outside) for each age and sex class.


| sex | age | m12 | m12.l | m12.u | m21 | m21.l | m21.u | m13 | m13.l | m13.u | m23 | m23.l |
| --- | --- | --- | --- | --- | --- | --- | --- | --- | --- | --- | --- | --- |
| female | 1 | 0.01 | 0 | 0.04 | 0.08 | 0.01 | 0.04 | 0.01 | 0 | 0.15 | 0.04 | 0.00 |
| female | 2 | 0.00 | 0 | 0.00 | 0.01 | 0.00 | 0.00 | 0.00 | 0 | 0.02 | 0.01 | 0.00 |
| female | 3 | 0.00 | 0 | 0.00 | 0.01 | 0.00 | 0.00 | 0.00 | 0 | 0.02 | 0.01 | 0.00 |
| male | 1 | 0.02 | 0 | 0.07 | 0.06 | 0.00 | 0.19 | 0.01 | 0 | 0.21 | 0.07 | 0.01 |
| male | 2 | 0.00 | 0 | 0.01 | 0.01 | 0.00 | 0.02 | 0.00 | 0 | 0.01 | 0.00 | 0.00 |
| male | 3 | 0.00 | 0 | 0.01 | 0.01 | 0.00 | 0.02 | 0.00 | 0 | 0.01 | 0.00 | 0.00 |

# 4 Sensitivity to including both L and R lynxes

When both L and R individuals are included, survival will be underestimated because if one individual dies there might two “individuals” in the data (an R and an L belonging to the same individual) disappear. However, if we reduce the data to one of L or R individuals, because individuals that die early may be overrepresented among those deleted from the data by such a selection. Therefore, we refitted the model to data that were not reduced. We found that survival estimates were slightly lower for juveniles whereas for the other age classes, survival estimates were similar when the model was fitted to the non-reduced data.

```
load("modelfits/modelfit_alps_combined_ageyearinklRL_231201.rda") # m per sex and age

S1 <- plogis(apply(mod$sims.list$b10, c(2,3), mean))
S1lwr <- plogis(apply(mod$sims.list$b10, c(2,3), quantile, probs=0.025))
S1upr <- plogis(apply(mod$sims.list$b10, c(2,3), quantile, probs=0.975))
 
S2 <- plogis(apply(mod$sims.list$b20, c(2,3), mean))
S2lwr <- plogis(apply(mod$sims.list$b20, c(2,3), quantile, probs=0.025))
S2upr <- plogis(apply(mod$sims.list$b20, c(2,3), quantile, probs=0.975))

tab <- expand.grid(age=c("juveniles", "subadult", "adult"), 
                   sex=c("females", "males"))
tab$Ia_S <- as.numeric(t(S1))
tab$Ia_S.lwr <- as.numeric(t(S1lwr))
tab$Ia_S.upr <- as.numeric(t(S1upr))
tab$Ib_S <- as.numeric(t(S2))
tab$Ib_S.lwr <- as.numeric(t(S2lwr))
tab$Ib_S.upr <- as.numeric(t(S2upr))
kable(tab, dig=2, caption="Average annual survival estimates from the combined model with 95% uncertainty interval fitted to data including both L and R lynxes.")
```

Table 4.1: Average annual survival estimates from the combined model with 95% uncertainty interval fitted to data including both L and R lynxes.


| age | sex | Ia\_S | Ia\_S.lwr | Ia\_S.upr | Ib\_S | Ib\_S.lwr | Ib\_S.upr |
| --- | --- | --- | --- | --- | --- | --- | --- |
| juveniles | females | 0.24 | 0.11 | 0.45 | 0.32 | 0.14 | 0.58 |
| subadult | females | 0.92 | 0.72 | 0.99 | 0.76 | 0.46 | 0.94 |
| adult | females | 0.75 | 0.69 | 0.80 | 0.84 | 0.76 | 0.91 |
| juveniles | males | 0.24 | 0.11 | 0.47 | 0.24 | 0.11 | 0.46 |
| subadult | males | 0.76 | 0.53 | 0.91 | 0.85 | 0.65 | 0.95 |
| adult | males | 0.75 | 0.69 | 0.80 | 0.78 | 0.71 | 0.84 |

Maronde, Lea, Brett T. McClintock, Urs Breitenmoser, and Fridolin Zimmermann. 2020. “Spatial Capture–Recapture with Multiple Noninvasive Marks: An Application to Camera-Trapping Data of the European Wildcat (Felis Silvestris) Using r Package Multimark.” *Ecology and Evolution* 10 (24): 13968–79. https://doi.org/10.1002/ece3.6990.

Mayfield, Harold F. 1975. “Suggestions for Calculating Nest Success.” *Wilson Bulletin* 87: 456–66.

Nichols, James D, William L Kendall, James E Hines, and Jeffrey A Spendelow. 2004. “Estimation of Sex-Specific Survival from Capture-Recapture Data When Sex Is Not Always Known.” *Ecology* 85: 3192–3201.

Weidinger, Karel. 2007. “Handling of Uncertain Nest Fates and Variation in Nest Survival Estimates.” *Journal of Ornithology* 148: 207–13.
