# Supplementary material for: Long‐Term Changes in Survival of Eurasian Lynx in Three Reintroduced Populations in Switzerland
Source: Ecol Evol. 2025 Mar 30;15(4):e71095. doi: 10.1002/ece3.71095 (PMC11955280; doi:10.1002/ece3.71095)
Supplement: Supplementary file 1 — Appendix S1. [file ECE3-15-e71095-s001.zip › Supplementary_material__README_NECH.html]

Documentation and code of the survival model for the NE-CH population


Code 

- Show All Code
- Hide All Code

# Documentation and code of the survival model for the NE-CH population

#### Vogt et al.

#### 2025-01-10

```
library(knitr)
library(R2jags)
```

# 1 Data

## 1.1 Picture and telemetry data

```
load("data/dataxNECH.rda")
str(datax)
```

```
## List of 13
##  $ obs       : num [1:133, 1:253] 5 5 5 5 5 5 5 5 5 5 ...
##  $ first     : num [1:133] 25 88 2 25 60 59 57 55 86 92 ...
##  $ sex       : num [1:133] 1 1 1 1 NA NA 1 NA NA 2 ...
##  $ nind      : int 133
##  $ last      : int [1:133] 253 253 253 253 253 253 253 253 253 253 ...
##  $ age       : num [1:133, 1:22] NA NA 2 NA NA NA NA NA NA NA ...
##  $ monitoring: num [1:253] 1 1 1 1 1 1 1 1 1 1 ...
##  $ oppeff    : num [1:253] 2.67 2.67 2.67 2.67 2.67 ...
##  $ telemetry : num [1:133, 1:253] 1 1 2 1 1 1 1 1 1 1 ...
##  $ alpha     : num [1:2] 1 1
##  $ nyears    : num 22
##  $ year      : num [1:253] 1 1 2 2 2 2 2 2 2 2 ...
##  $ lunoinout : num [1:5] 0 1 0 1 0
```

The data object `datax` contains the following elements:

- `obs`: observation matrix with one row per individual and one column per month. The entries specify the categorised observation (1= detected alive in the study area, 2 = detected alive outside the study area, 3 = recovered dead in the study area, 4 = recovered dead outside the study area, 5= not detected or recovered during that month.)
- `first`: month of first release or first detection of the individual
- `sex`: sex per individual (1 = female, 2 = male, NA = unknown)
- `nind`: number of individuals in the data
- `last`: last month per individual that should be included in the data. For most individuals this is the last month of the study period. For individuals that were translocated to a different study area, it is the month of translocation (censoring of data at transolcation).
- `age`: matrix with rows corresponding to individuals and columns to lynx years. Values 1 = lynx in its first year, 2 = lynx in its second year, and 3 = older than second year.
- `monitoring`: category of monitoring per month (1 = opportunistic, 2 = deterministic)
- `oppeff`: observation effort per month. The larger the value the more cameras were deployed in the study area. This variable referst to the opportunistic monitoring, thus it does not include the number of cameras of the deterministic monitoring for which a separate detection probability is estimated.
- `telemetry`: specificator when lynxes were tagged by a telemetry device (1 = no telemetry device, 2 = with telemetry device)  
  `
- `alpha`: parameter values of prior dirichlet distribution for the proportion of females and males.
- `nyears`: number of years in the study period
- `year`: assignment of monthes to lynx years
- `lunoinout`: indicator of emigration for each state (0 = inside study area (not emigrated), 1 = outside study area (emigrated))

Graphical display of age categories per individual and year:

```
image(t(datax$age))
```

Figure 1.1: Age categories (darker colour from 1 to 3) for each individual and year.

```
colkey <- c(rainbow(4), "white")

ch <- datax$obs[order(datax$first),]
par(mar=c(3,4,0.1,0.1))
plot(seq(1, ncol(datax$obs), length=nrow(datax$obs)),
    1:nrow(datax$obs), type="n", xlab="Month", yaxt="n", ylab=NA)
axis(2, at=1:nrow(datax$obs), 1:nrow(datax$obs), las=1, cex.axis=0.6)
 nocc <- ncol(datax$obs)
for(i in 1:nrow(datax$obs)){
  points(1:nocc, rep(i, nocc), pch=15, col=colkey[ch[i,]])
  }
legend(0, nrow(ch), pch=15, col=colkey, 
       legend=c("pictured/localised alive in NE-CH", "pictured/localised alive emigrated",  "found dead in NE-CH", "found dead emigrated", NA), bty="n", cex=0.8)
```

Figure 1.2: Histories of the individuals. Month 1 = März 2001.

## 1.2 Age at death data

```
load("data/ageatdeath_data.rda")
str(djags)
```

```
## List of 6
##  $ y       : num [1:37] 1 1 1 1 1 1 1 1 1 1 ...
##  $ ageclass: num [1:8] 1 2 3 3 3 3 3 3
##  $ sex     : num [1:37] 1 2 2 2 1 1 NA 1 1 1 ...
##  $ nyears  : num 8
##  $ nind    : int 37
##  $ alpha   : num [1:2] 1 1
```

```
hist(djags$y, main=NA, xlab="Age at death (y)")
```

Figure 1.3: Number of individuals found dead per age in years.

The data contain the following elements:

- `y`: age (in years) at which the lynx has been recovered dead
- `ageclass`: classification of the years in age classes (1 = first year, 2 = second year, 3 = older)
- `sex`: sex per individual (1 = female, 2 = male)
- `nyears`: maximum age
- `nind`: number of individuals
- `alpha`: parameter values for dirichlet prior distribution for the proportion of females and males.

# 2 Integrated model for picture, telemetry and age at death data

## 2.1 Data

```
datax <- list(obs=datax$obs, first=datax$first,
              sex=datax$sex,
              #nocc=datax$nocc,
              nind=datax$nind,
              last=datax$last,
              age=datax$age,
              monitoring=datax$monitoring, 
              oppeff=datax$oppeff, 
              telemetry=datax$telemetry,
              alpha=c(1,1),
              nyears=datax$nyears,
              year=datax$year,
              # age at death data
              y=djags$y,
              ageclass=djags$ageclass,
              adsex=djags$sex,
              adnyears=djags$nyears,
              adnind=djags$nind,
              lunoinout=c(0,1,0,1,0))
```

## 2.2 Model code

```
cat(readLines('jags/Smod_luno_btocombined_ageyearint_ragedep.txt'), sep = '\n')
```

```
## # integrates the multi-state model with the BTO-dead recovery model
## 
## # for the multi-state model for combining camera trap data and lynx found dead
## # data:
## ## obs: observation history matrix (nind x nocc) with the observations (see below)
## ## first: month of first capture (marking)
## ## nocc: number of capture occasions (month)
## 
## # predictors:
## ## sex: vector of length nind with values 1, 2 and NA
## ## age: matrix with 3 age classes
## 
## 
## #states:
## #1 = alive in LUNO 
## #2 = alive outside LUNO  
## #3 = died within that month in LUNO  
## #4 = died within that month outside LUNO 
## #5 = dead before that month  
## 
## #observations:
## #1 = pictured at least once during the month in LUNO
## #2 = pictured at least once during the month outside of LUNO
## #3 = found dead in LUNO  
## #4 = found dead outside LUNO  
## #5 = not seen or found
## 
## # for the BTO dead recovery model
## 
## # data
## # y= vector with ages at death (1 = death in first year, 2 = death in second year, 3 = death later)
## # ad.sex = vector with sexes (1=female, 2 = male, NA=not identified)
## # ageclass = vector with ageclasses 1:3
## # ad.nyears: maximum age in years
## # ad.nind: number of individuals in data set with age at death
## 
## model{
## 
##   ## transition probabilities
## for(i in 1:nind){
##   for(t in first[i]:(last[i]-1)){
##   ps[1,i,t,1] <- (1-m[i, year[t]])*pow(s[i,year[t]],1/12)
##   ps[1,i,t,2] <- m[i, year[t]]*pow(s[i,year[t]],1/12)
##   ps[1,i,t,3] <- 1-pow(s[i,year[t]], 1/12)
##   ps[1,i,t,4] <- 0
##   ps[1,i,t,5] <- 0
## 
##   ps[2,i,t,1] <- 0
##   ps[2,i,t,2] <- pow(s[i,year[t]],1/12)
##   ps[2,i,t,3] <- 0
##   ps[2,i,t,4] <- 1-pow(s[i,year[t]], 1/12)
##   ps[2,i,t,5] <- 0
## 
##   ps[3,i,t,1] <- 0
##   ps[3,i,t,2] <- 0
##   ps[3,i,t,3] <- 0
##   ps[3,i,t,4] <- 0
##   ps[3,i,t,5] <- 1
## 
##   ps[4,i,t,1] <- 0
##   ps[4,i,t,2] <- 0
##   ps[4,i,t,3] <- 0
##   ps[4,i,t,4] <- 0
##   ps[4,i,t,5] <- 1
## 
##   ps[5,i,t,1] <- 0
##   ps[5,i,t,2] <- 0
##   ps[5,i,t,3] <- 0
##   ps[5,i,t,4] <- 0
##   ps[5,i,t,5] <- 1 
## 
##   po[1,i,t,1] <- p[i,t]
##   po[1,i,t,2] <- 0
##   po[1,i,t,3] <- 0
##   po[1,i,t,4] <- 0
##   po[1,i,t,5] <- 1-p[i,t]
## 
##   po[2,i,t,1] <- 0
##   po[2,i,t,2] <- p[i,t]
##   po[2,i,t,3] <- 0
##   po[2,i,t,4] <- 0
##   po[2,i,t,5] <- 1-p[i,t]
## 
##   po[3,i,t,1] <- 0
##   po[3,i,t,2] <- 0
##   po[3,i,t,3] <- r[i,t]
##   po[3,i,t,4] <- 0
##   po[3,i,t,5] <- 1-r[i,t]
## 
##   po[4,i,t,1] <- 0
##   po[4,i,t,2] <- 0
##   po[4,i,t,3] <- 0
##   po[4,i,t,4] <- r[i,t]
##   po[4,i,t,5] <- 1-r[i,t]
## 
##   po[5,i,t,1] <- 0
##   po[5,i,t,2] <- 0
##   po[5,i,t,3] <- 0
##   po[5,i,t,4] <- 0
##   po[5,i,t,5] <- 1
##   } # t
## } # i
## 
##   ## likelihood
##   for(i in 1:nind){
##     z[i,first[i]]  <- obs[i,first[i]]
##     area[i,first[i]] <- lunoinout[z[i,first[i]]]
##     for(t in (first[i]+1):last[i]) {
##       z[i,t] ~ dcat(ps[z[i,t-1], i, t-1, ])
##       area[i,t] <- lunoinout[z[i,t]]
##       obs[i,t] ~ dcat(po[z[i,t], i, t-1,])
##     }
##   }
## 
##   ## linear predictors
##   for(i in 1:nind){
##     ## impute unknown sexes
##     sex[i] ~ dcat(propsex)
##     for(v in year[first[i]]:nyears){
##       m[i,v] <- m0[sex[i], age[i,v]]
##       logit(s[i,v]) <-  b0[sex[i], age[i,v]] + sigmaSyear[age[i,v]]*yearSeff[age[i,v],v] #b0[1, age[i,t]]# 
##     }
##     for(t in first[i]:(last[i]-1)){
##       logit(p[i,t]) <- a0[sex[i], age[i,year[t]], monitoring[t], telemetry[i,t]] + a1*area[i,t] + a2*oppeff[t]+ sigmapind*indeff[i]  
##       logit(r[i,t]) <-  d0[sex[i], age[i,year[t]]]
##     }
##   }
## 
##   ## priors
##   for(i in 1:nind){
##     indeff[i]~dnorm(0,1)
##   }
##   sigmapind ~ dt(0,1,2)I(0,)
## 
##   for(i in 1:nyears){
##     yearSeff[1,i]~dnorm(0,1)
##     yearSeff[2,i]~dnorm(0,1)
##     yearSeff[3,i]~dnorm(0,1)
##   }
##   sigmaSyear[1] ~ dt(0,1,2)I(0,)
##   sigmaSyear[2] ~ dt(0,1,2)I(0,)
##   sigmaSyear[3] ~ dt(0,1,2)I(0,)
## 
##   propsex[1:2] ~ ddirch(alpha[1:2])
## 
##   b0[1,1] ~ dnorm(0, 0.44)  # = Normal(0,1.5) für Intercept 
##   b0[2,1] ~ dnorm(0, 0.44)
##   b0[1,2] ~ dnorm(0, 0.44)
##   b0[2,2] ~ dnorm(0, 0.44)
##   b0[1,3] ~ dnorm(0, 0.44)
##   b0[2,3] ~ dnorm(0, 0.44)
## 
##   a0[1,1,1,1] ~ dnorm(0, 0.44)
##   a0[1,1,2,1] ~ dnorm(0, 0.44)
##   a0[2,1,1,1] ~ dnorm(0, 0.44)
##   a0[2,1,2,1] ~ dnorm(0, 0.44)
## 
##   a0[1,2,1,1] ~ dnorm(0, 0.44)
##   a0[1,2,2,1] ~ dnorm(0, 0.44)
##   a0[2,2,1,1] ~ dnorm(0, 0.44)
##   a0[2,2,2,1] ~ dnorm(0, 0.44)
## 
##   a0[1,3,1,1] ~ dnorm(0, 0.44)
##   a0[1,3,2,1] ~ dnorm(0, 0.44)
##   a0[2,3,1,1] ~ dnorm(0, 0.44)
##   a0[2,3,2,1] ~ dnorm(0, 0.44)
## 
## 
##   a0[1,1,1,2] ~ dnorm(0, 0.44) # lynxes with radio telemetry have all the same detection prob independent of sex and monitoring
##   a0[1,1,2,2] <- a0[1,1,1,2]
##   a0[2,1,1,2] <- a0[1,1,1,2]
##   a0[2,1,2,2] <- a0[1,1,1,2]
## 
##   a0[1,2,1,2] ~ dnorm(0, 0.44) # lynxes with radio telemetry have all the same detection prob independent of sex and monitoring
##   a0[1,2,2,2] <- a0[1,2,1,2]
##   a0[2,2,1,2] <- a0[1,2,1,2]
##   a0[2,2,2,2] <- a0[1,2,1,2]
##  
##   a0[1,3,1,2] ~ dnorm(0, 0.44) # lynxes with radio telemetry have all the same detection prob independent of sex and monitoring
##   a0[1,3,2,2] <- a0[1,3,1,2]
##   a0[2,3,1,2] <- a0[1,3,1,2]
##   a0[2,3,2,2] <- a0[1,3,1,2]
##    
## 
##   a1 ~ dnorm(0, 0.04) 
##   a2 ~ dnorm(0, 0.04) 
##   
##   d0[1,1] ~ dnorm(0, 0.44)
##   d0[2,1] ~ dnorm(0, 0.44)
##   d0[1,2] ~ dnorm(0, 0.44)
##   d0[2,2] ~ dnorm(0, 0.44)
##   d0[1,3] ~ dnorm(0, 0.44)
##   d0[2,3] ~ dnorm(0, 0.44)
##   m0[1,1] ~ dbeta(1,1)
##   m0[2,1] ~ dbeta(1,1)
##   m0[1,2] ~ dbeta(1,1)
##   m0[2,2] ~ dbeta(1,1)
##   m0[1,3] ~ dbeta(1,1)
##   m0[2,3] ~ dbeta(1,1)
## 
## 
## # model for age at death
##   for(s in 1:2){
##     for(a in 1:3){
##       logit(rsa[s,a]) <- d0[s,a] # get recovery probabilty per sex and ageclass 
##       logit(Ssa[s,a]) <- b0[s,a] # get survival per sex and ageclass
##     }
##   # propabiliy of dying within 8 years and beeing found
##   pfounddead[s]  <- (1-Ssa[s,1])*rsa[s,1] + Ssa[s,1]*(1-Ssa[s,2])*rsa[s,2] + 
##                         Ssa[s,1]*Ssa[s,2]*(1-Ssa[s,3])*rsa[s,3]*(pow(Ssa[s,3],8-2)-1)/(Ssa[s,3]-1)
## 
##   }
## 
## # likelihood
##   for(i in 1:adnind){
##     y[i] ~ dcat(adp[i,1:adnyears]) # y[i]: year of dead recovery of individual i
##     
##     adp[i,1] <- (1-adS[i,1])*rsa[adsex[i], ageclass[1]]/pfounddead[adsex[i]]
##     adsex[i] ~ dcat(propsex)
##     for(j in 2:adnyears){ 
##       adp[i,j] <- prod(adS[i,1:(j-1)])*(1-adS[i,j])*rsa[adsex[i], ageclass[j]]/pfounddead[adsex[i]]
##     }
##     for(j in 1:adnyears){
##       logit(adS[i,j]) <- b0[adsex[i],ageclass[j]] # same intercept as in multi-state model
##     }
##   }
## }
```

```
# prepare initial values
last <- apply(datax$obs, 1, function(x) max(c(1:nocc)[x<5], na.rm=TRUE))
initz <- matrix(5, ncol=ncol(datax$obs), nrow=nrow(datax$obs))
for(i in 1:nrow(datax$obs)) {
      initz[i, datax$first[i]:last[i]] <- 1
      if(last[i]<nocc) initz[i, (last[i]+1)] <- 3
      if(sum(datax$obs[i,]==2,na.rm=TRUE)>0){
        ii <- datax$obs[i,]==2; ii[is.na(ii)] <- FALSE
        tt <- min(c(1:nocc)[ii])
        initz[i, tt:nocc] <- 2
      }
      if(sum(datax$obs[i,]==3, na.rm=TRUE)>0){
        ii <- datax$obs[i,]==3; ii[is.na(ii)] <- FALSE
        tt <- min(c(1:nocc)[ii])
        initz[i, tt:nocc] <- 3
        if(tt<nocc) initz[i, (tt+1):nocc] <- 5
      }
      if(sum(datax$obs[i,]==4, na.rm=TRUE)>0){
        ii <- datax$obs[i,]==4; ii[is.na(ii)] <- FALSE
        tt <- min(c(1:nocc)[ii])
        initz[i, tt] <- 4
        initz[i, tt-1] <- 2
        if(tt<nocc) initz[i, (tt+1):nocc] <- 5
      }
      initz[i, 1:datax$first[i]] <- NA
      initz[is.na(datax$obs)] <- NA
}

    
initfun <- function(){
  a0=array(runif(2*3*2*2,-0.5,0.5), dim=c(2,3,2,2))
  a0[1,1,2,2] <- NA
  a0[2,,,2] <- NA
      list(b0=matrix(runif(2*3,-0.5,0.5), ncol=3, nrow=2),
           a0=a0,
           a1=runif(1, -0.5,0.5),
           a2=runif(1, -0.5,0.5),
           d0=matrix(runif(2*3,-0.5,0.5), ncol=3, nrow=2),
           m0=matrix(runif(2*3, 0,1), ncol=3, nrow=2),
           sigmapind=runif(1, 0.1, 0.4),
           sigmaSyear=runif(3, 0.1, 0.4),
           z=initz)
}
```

```
mod <- jags(datax, inits=initfun, parameters.to.save=c("a0", "a1", "a2", "b0", "d0", "m0", "sigmapind", "indeff", "sigmaSyear", "yearSeff", "propsex", "sex"),
            model.file="jags/Smod_luno_btocombined_ageyearint_ragedep.txt", n.chains=3, n.iter=40000, n.thin=5)

mod <- mod$BUGSoutput
save(mod, file="modelfits/modelfit_luno_combined_ageyear231022.rda")
```

## 2.3 Results

```
load("modelfits/modelfit_luno_combined_ageyear241205.rda") # m per sex and age


tab <- mod$summary[c(1:28,159:166, 297:300),]
kable(tab, dig=2, caption="Summary of the marginal posterior distributions of the model parameters in the integrated model. For interpretation of the model parameters see first table.")
```

Table 2.1: Summary of the marginal posterior distributions of the model parameters in the integrated model. For interpretation of the model parameters see first table.


|  | mean | sd | 2.5% | 25% | 50% | 75% | 97.5% | Rhat | n.eff |
| --- | --- | --- | --- | --- | --- | --- | --- | --- | --- |
| a0[1,1,1] | -0.29 | 0.46 | -1.20 | -0.60 | -0.29 | 0.02 | 0.60 | 1 | 2000 |
| a0[2,1,1] | 0.80 | 0.45 | -0.11 | 0.50 | 0.80 | 1.10 | 1.69 | 1 | 1900 |
| a0[1,2,1] | 0.12 | 0.41 | -0.68 | -0.16 | 0.12 | 0.39 | 0.94 | 1 | 1500 |
| a0[2,2,1] | 0.29 | 0.43 | -0.54 | 0.01 | 0.29 | 0.57 | 1.12 | 1 | 890 |
| a0[1,3,1] | 0.22 | 0.40 | -0.59 | -0.05 | 0.22 | 0.48 | 1.01 | 1 | 1000 |
| a0[2,3,1] | 0.55 | 0.41 | -0.25 | 0.28 | 0.55 | 0.81 | 1.35 | 1 | 1200 |
| a0[1,1,2] | 100.00 | 0.00 | 100.00 | 100.00 | 100.00 | 100.00 | 100.00 | 1 | 1 |
| a0[2,1,2] | 100.00 | 0.00 | 100.00 | 100.00 | 100.00 | 100.00 | 100.00 | 1 | 1 |
| a0[1,2,2] | 100.00 | 0.00 | 100.00 | 100.00 | 100.00 | 100.00 | 100.00 | 1 | 1 |
| a0[2,2,2] | 100.00 | 0.00 | 100.00 | 100.00 | 100.00 | 100.00 | 100.00 | 1 | 1 |
| a0[1,3,2] | 100.00 | 0.00 | 100.00 | 100.00 | 100.00 | 100.00 | 100.00 | 1 | 1 |
| a0[2,3,2] | 100.00 | 0.00 | 100.00 | 100.00 | 100.00 | 100.00 | 100.00 | 1 | 1 |
| a1 | -2.48 | 0.49 | -3.47 | -2.80 | -2.47 | -2.14 | -1.55 | 1 | 12000 |
| a2 | -0.67 | 0.19 | -1.03 | -0.79 | -0.67 | -0.54 | -0.29 | 1 | 1100 |
| a3 | 1.02 | 0.16 | 0.70 | 0.91 | 1.02 | 1.13 | 1.34 | 1 | 12000 |
| b0[1,1] | -0.91 | 0.79 | -2.38 | -1.46 | -0.95 | -0.38 | 0.73 | 1 | 3700 |
| b0[2,1] | -1.18 | 0.79 | -2.53 | -1.74 | -1.25 | -0.67 | 0.55 | 1 | 8200 |
| b0[1,2] | 1.10 | 0.48 | 0.20 | 0.77 | 1.09 | 1.42 | 2.10 | 1 | 12000 |
| b0[2,2] | 1.21 | 0.58 | 0.14 | 0.82 | 1.19 | 1.59 | 2.47 | 1 | 6100 |
| b0[1,3] | 1.16 | 0.25 | 0.69 | 0.98 | 1.15 | 1.31 | 1.68 | 1 | 7900 |
| b0[2,3] | 1.16 | 0.26 | 0.67 | 0.98 | 1.15 | 1.33 | 1.71 | 1 | 12000 |
| d0[1,1] | -1.94 | 0.84 | -3.40 | -2.52 | -2.00 | -1.43 | -0.11 | 1 | 7600 |
| d0[2,1] | -2.54 | 0.78 | -3.97 | -3.06 | -2.58 | -2.05 | -0.84 | 1 | 12000 |
| d0[1,2] | 0.08 | 0.79 | -1.34 | -0.47 | 0.02 | 0.55 | 1.82 | 1 | 7000 |
| d0[2,2] | -2.50 | 1.02 | -4.52 | -3.17 | -2.49 | -1.83 | -0.48 | 1 | 12000 |
| d0[1,3] | -2.39 | 0.51 | -3.46 | -2.72 | -2.36 | -2.04 | -1.45 | 1 | 12000 |
| d0[2,3] | -1.32 | 0.41 | -2.18 | -1.60 | -1.31 | -1.03 | -0.54 | 1 | 12000 |
| deviance | 3281.14 | 14.12 | 3254.98 | 3271.43 | 3280.53 | 3290.46 | 3310.20 | 1 | 9200 |
| m0[1,1] | 0.01 | 0.01 | 0.00 | 0.00 | 0.00 | 0.01 | 0.02 | 1 | 12000 |
| m0[2,1] | 0.01 | 0.01 | 0.00 | 0.01 | 0.01 | 0.02 | 0.04 | 1 | 12000 |
| m0[1,2] | 0.00 | 0.00 | 0.00 | 0.00 | 0.00 | 0.00 | 0.00 | 1 | 12000 |
| m0[2,2] | 0.00 | 0.00 | 0.00 | 0.00 | 0.00 | 0.00 | 0.00 | 1 | 2900 |
| m0[1,3] | 0.00 | 0.00 | 0.00 | 0.00 | 0.00 | 0.00 | 0.00 | 1 | 12000 |
| m0[2,3] | 0.00 | 0.00 | 0.00 | 0.00 | 0.00 | 0.00 | 0.00 | 1 | 2900 |
| propsex[1] | 0.53 | 0.07 | 0.40 | 0.48 | 0.53 | 0.58 | 0.66 | 1 | 2500 |
| propsex[2] | 0.47 | 0.07 | 0.34 | 0.42 | 0.47 | 0.52 | 0.60 | 1 | 2200 |
| sigmaSyear[1] | 0.98 | 0.56 | 0.07 | 0.57 | 0.93 | 1.32 | 2.20 | 1 | 5700 |
| sigmaSyear[2] | 0.67 | 0.49 | 0.03 | 0.29 | 0.59 | 0.96 | 1.83 | 1 | 12000 |
| sigmaSyear[3] | 0.44 | 0.24 | 0.03 | 0.27 | 0.43 | 0.59 | 0.93 | 1 | 12000 |
| sigmapind | 0.44 | 0.08 | 0.29 | 0.38 | 0.43 | 0.49 | 0.59 | 1 | 4300 |

```
# check convergence
 # plot(mod$sims.array[,1,1], type="l")
 # lines(mod$sims.array[,2,1], col=2)
 # lines(mod$sims.array[,3,1], col=3)

  # plot(mod$sims.array[,1,10], type="l")
  # lines(mod$sims.array[,2,10], col=2)
  # lines(mod$sims.array[,3,10], col=3)
```

```
S <- plogis(apply(mod$sims.list$b0, c(2,3), mean))
Slwr <- plogis(apply(mod$sims.list$b0, c(2,3), quantile, probs=0.025))
Supr <- plogis(apply(mod$sims.list$b0, c(2,3), quantile, probs=0.975))
   
plot(1:3, seq(0,1, length=3), type="n", las=1, xaxt="n", xlab="", ylim=c(0,1), ylab="Annual survival", xlim=c(0.5, 3.5))
#abline(h=0.5, col=grey(0.8), lwd=2) # prior mean
# females
segments(c(1:3)+0.1, Slwr[1,], c(1:3)+0.1, Supr[1,],lwd=2, lend="butt", col="orange")
points(c(1:3)+0.1, S[1,], pch=21, col="orange", bg="white")
# males
segments(c(1:3)-0.1, Slwr[2,], c(1:3)-0.1, Supr[2,],lwd=2, lend="butt", col="blue")
points(c(1:3)-0.1, S[2,], pch=21, col="blue", bg="white")
axis(1, at=1:3, labels=c("age 1", "age 2", "age 3"))
legend(0.5, 1.25, xpd=NA, lwd=2, col=c("orange", "blue"), pch=21, bg="white", legend=c("females", "males"), horiz=TRUE, bty="n")
```

Figure 2.1: Estimated average annual apparent survival probability per age class based on combined data. Vertical bars are 95% compatibility intervals.

```
tab <- expand.grid(age=c("First year", "Second year", "Adult"), 
                   sex=c("females", "males"))
tab$S <- as.numeric(t(S))
tab$S.lwr <- as.numeric(t(Slwr))
tab$S.upr <- as.numeric(t(Supr))
kable(tab, dig=2, caption="Average annual survival estimates from the combined model with 95% uncertainty interval.")
```

Table 2.2: Average annual survival estimates from the combined model with 95% uncertainty interval.

| age | sex | S | S.lwr | S.upr |
| --- | --- | --- | --- | --- |
| First year | females | 0.29 | 0.08 | 0.67 |
| Second year | females | 0.75 | 0.55 | 0.89 |
| Adult | females | 0.76 | 0.67 | 0.84 |
| First year | males | 0.24 | 0.07 | 0.63 |
| Second year | males | 0.77 | 0.53 | 0.92 |
| Adult | males | 0.76 | 0.66 | 0.85 |

```
nsim <- mod$n.sims
Spyear <- array(NA, dim=c(2,3, 22, nsim))
for(i in 1:2){
  for(j in 1:3){
    Spyear[i,j,,] <- matrix(mod$sims.list$b0[,i,j], ncol=nsim, nrow=22, byrow=TRUE)+ matrix(mod$sims.list$sigmaSyear[,j], ncol=nsim, nrow=22, byrow=TRUE)*t(mod$sims.list$yearSeff[,j,1:22])
  }
}
   
Spyearm <- plogis(apply(Spyear, c(1,2,3), mean))
Spyearlwr <- plogis(apply(Spyear, c(1,2,3), quantile, probs=0.025))
Spyearupr <- plogis(apply(Spyear, c(1,2,3), quantile, probs=0.975))


par(mfrow=c(3,1), mar=c(0.5, 3,1.5, 0.5), oma=c(3,3,0.5,0))
plot(1:22, seq(0,1, length=22), type="n", las=1, xaxt="n", xlab="", ylim=c(0,1), ylab="Annual survival", xlim=c(1,22))
legend(13, 1.05, xpd=NA, lwd=2, col=c("orange", "blue"), pch=21, bg="white", legend=c("females", "males"), horiz=TRUE, bty="n")
mtext("Adults", adj=0, side=3)

segments(c(1:22)+0.1, Spyearlwr[1,3,], c(1:22)+0.1, Spyearupr[1,3,],lwd=2, lend="butt", col="orange")
points(c(1:22)+0.1, Spyearm[1,3,], pch=21, col="orange", bg="white")

segments(c(1:22)-0.1, Spyearlwr[2,3,], c(1:22)-0.1, Spyearupr[2,3,],lwd=2, lend="butt", col="blue")
points(c(1:22)-0.1, Spyearm[2,3,], pch=21, col="blue", bg="white")

plot(1:22, seq(0,1, length=22), type="n", las=1, xaxt="n", xlab="", ylim=c(0,1), ylab="Annual survival")
segments(c(1:22)+0.1, Spyearlwr[1,2,], c(1:22)+0.1, Spyearupr[1,2,],lwd=2, lend="butt", col="orange")
points(c(1:22)+0.1, Spyearm[1,2,], pch=21, col="orange", bg="white")
segments(c(1:22)-0.1, Spyearlwr[2,2,], c(1:22)-0.1, Spyearupr[2,2,],lwd=2, lend="butt", col="blue")
points(c(1:22)-0.1, Spyearm[2,2,], pch=21, col="blue", bg="white")
mtext("Subadults", adj=0, side=3)

plot(1:22, seq(0,1, length=22), type="n", las=1, xaxt="n", xlab="", ylim=c(0,1), ylab="Annual survival")
segments(c(1:22)+0.1, Spyearlwr[1,1,], c(1:22)+0.1, Spyearupr[1,1,],lwd=2, lend="butt", col="orange")
points(c(1:22)+0.1, Spyearm[1,1,], pch=21, col="orange", bg="white")
segments(c(1:22)-0.1, Spyearlwr[2,1,], c(1:22)-0.1, Spyearupr[2,1,],lwd=2, lend="butt", col="blue")
points(c(1:22)-0.1, Spyearm[2,1,], pch=21, col="blue", bg="white")
mtext("Juveniles", adj=0, side=3)
mtext("Annual survival", side=2, outer=TRUE, line=0.5)

axis(1, at=1:22, labels=paste0(2000:2021))
```

Figure 2.2: Estimated annual apparent survival probability for adults based on combined data. Vertical bars are 95% compatibility intervals. Orange=females, blue=males.

```
# probability to be pictured during one month 
# Dimension 1: 1. females, 2. males
# Dimension 2: age classes 1,2,3
# deleted in final version: Dimension 3: 1. opportunistisch,2. determinisitsch
# Dimension 4: 1. ohne und 2. mit Telemetrie

tab <- data.frame(sex=c("females", "females", "females", "males", "males", "males"),
                  age=c(1,2,3,1,2,3))

tab$p_opp <- plogis(c(apply(mod$sims.list$a0[,1,,1], 2, mean),
                      apply(mod$sims.list$a0[,2,,1], 2, mean)))
tab$p_opp.lwr <- plogis(c(apply(mod$sims.list$a0[,1,,1], 2, quantile, prob=0.025),
                      apply(mod$sims.list$a0[,2,,1], 2, quantile, prob=0.025)))
tab$p_opp.upr <- plogis(c(apply(mod$sims.list$a0[,1,,1], 2, quantile, prob=0.975),
                      apply(mod$sims.list$a0[,2,,1], 2, quantile, prob=0.975)))
# tab$p_det <-  plogis(c(apply(mod$sims.list$a0[,1,,2,1], 2, mean),
#                       apply(mod$sims.list$a0[,2,,2,1], 2, mean)))
# tab$p_det.lwr <-  plogis(c(apply(mod$sims.list$a0[,1,,2,1], 2, quantile, prob=0.025),
#                       apply(mod$sims.list$a0[,2,,2,1], 2, quantile, prob=0.025)))
# tab$p_det.upr <-  plogis(c(apply(mod$sims.list$a0[,1,,2,1], 2, quantile, prob=0.975),
#                       apply(mod$sims.list$a0[,2,,2,1], 2, quantile, prob=0.975)))
tab$p_telemetry <-  plogis(c(apply(mod$sims.list$a0[,1,,2], 2, mean),
                      apply(mod$sims.list$a0[,2,,2], 2, mean)))

tab$p_telemetry.lwr <-  plogis(c(apply(mod$sims.list$a0[,1,,2], 2, quantile, prob=0.025),
                      apply(mod$sims.list$a0[,2,,2], 2, quantile, prob=0.025)))
tab$p_telemetry.upr <-  plogis(c(apply(mod$sims.list$a0[,1,,2], 2, quantile, prob=0.975),
                      apply(mod$sims.list$a0[,2,,2], 2, quantile, prob=0.975)))
kable(tab, dig=2, caption="Probabilities to get pictured within one month for the different sexes and age classes in the opportunisitc monitoring and if tagged by telemetry. Estimates are obtained from the model for the combined data set including age at death data.")
```

Table 2.3: Probabilities to get pictured within one month for the different sexes and age classes in the opportunisitc monitoring and if tagged by telemetry. Estimates are obtained from the model for the combined data set including age at death data.


| sex | age | p\_opp | p\_opp.lwr | p\_opp.upr | p\_telemetry | p\_telemetry.lwr | p\_telemetry.upr |
| --- | --- | --- | --- | --- | --- | --- | --- |
| females | 1 | 0.43 | 0.23 | 0.64 | 1 | 1 | 1 |
| females | 2 | 0.53 | 0.34 | 0.72 | 1 | 1 | 1 |
| females | 3 | 0.55 | 0.36 | 0.73 | 1 | 1 | 1 |
| males | 1 | 0.69 | 0.47 | 0.84 | 1 | 1 | 1 |
| males | 2 | 0.57 | 0.37 | 0.75 | 1 | 1 | 1 |
| males | 3 | 0.63 | 0.44 | 0.79 | 1 | 1 | 1 |

The odds (=probability of getting pictured/probability of not getting pictured) of getting pictured outside NE-CH is only 0.08 times it is inside NE-CH.

```
tab <- data.frame(sex=c("females", "females", "females", "males", "males", "males"),
                  age=c(1,2,3,1,2,3))

tab$r <- plogis(c(apply(mod$sims.list$d0[,1,], 2, mean),
                      apply(mod$sims.list$d0[,2,], 2, mean)))
tab$r.lwr <- plogis(c(apply(mod$sims.list$d0[,1,], 2, quantile, prob=0.025),
                      apply(mod$sims.list$d0[,2,], 2, quantile, prob=0.025)))
tab$r.upr <- plogis(c(apply(mod$sims.list$d0[,1,], 2, quantile, prob=0.975),
                      apply(mod$sims.list$d0[,2,], 2, quantile, prob=0.975)))

kable(tab, dig=2, caption="Probability that a dead lynx is found from the model fitted to the combined data including age at death data.")
```

Table 2.4: Probability that a dead lynx is found from the model fitted to the combined data including age at death data.

| sex | age | r | r.lwr | r.upr |
| --- | --- | --- | --- | --- |
| females | 1 | 0.13 | 0.03 | 0.47 |
| females | 2 | 0.52 | 0.21 | 0.86 |
| females | 3 | 0.08 | 0.03 | 0.19 |
| males | 1 | 0.07 | 0.02 | 0.30 |
| males | 2 | 0.08 | 0.01 | 0.38 |
| males | 3 | 0.21 | 0.10 | 0.37 |

The probability to move out of the study area within one month decreases with age and it is lower for females compared to males (2.5).

```
nrr <- 2
tab <- data.frame(data=c("combined", "combined"),
                  sex=c("female", "male"),
                  m1y=rep(NA, nrr),
                  m1y.lwr=rep(NA, nrr),
                  m1y.upr=rep(NA, nrr),
                  m2y=rep(NA, nrr),
                  m2y.lwr=rep(NA, nrr),
                  m2y.upr=rep(NA, nrr),
                  mad=rep(NA, nrr),
                  mad.lwr=rep(NA, nrr),
                  mad.upr=rep(NA, nrr))
tab[1:2, c(1,4,7)+2] <- apply(mod$sims.list$m0, c(2,3), mean) 
tab[1:2, c(2,5,8)+2] <- apply(mod$sims.list$m0, c(2,3), quantile, prob=0.025)
tab[1:2, c(3,6,9)+2] <- apply(mod$sims.list$m0, c(2,3), quantile, prob=0.975)


kable(tab, dig=3, caption="Probability to permanently move out of NE-CH for each age class and sex estimated based on the combined data.")
```

Table 2.5: Probability to permanently move out of NE-CH for each age class and sex estimated based on the combined data.


| data | sex | m1y | m1y.lwr | m1y.upr | m2y | m2y.lwr | m2y.upr | mad | mad.lwr | mad.upr |
| --- | --- | --- | --- | --- | --- | --- | --- | --- | --- | --- |
| combined | female | 0.006 | 0.000 | 0.024 | 0.001 | 0 | 0.004 | 0.001 | 0 | 0.004 |
| combined | male | 0.014 | 0.001 | 0.039 | 0.001 | 0 | 0.003 | 0.001 | 0 | 0.003 |

Survivor curves and average age

```
# mean age equals -1/ln(S) if S is constant with age (exponential distribution)
# that is not the case -> Monte Carlo simulation to estimate mean age

nsim <- mod$n.sims
virtpop <- array(dim=c(nsim, 100, 2)) # max 100 years
virtpop[,1,] <- 1 # all individuals are once born
for(i in 2:100){
  # survival of first year
  if(i==2){ 
    virtpop[,i,1] <- rbinom(nsim, size=virtpop[,i-1,1], prob=plogis(mod$sims.list$b0[,1,1]))
    virtpop[,i,2] <- rbinom(nsim, size=virtpop[,i-1,2], prob=plogis(mod$sims.list$b0[,2,1]))

  }
  if(i==3){ 
    virtpop[,i,1] <- rbinom(nsim, size=virtpop[,i-1,1], prob=plogis(mod$sims.list$b0[,1,2]))
    virtpop[,i,2] <- rbinom(nsim, size=virtpop[,i-1,2], prob=plogis(mod$sims.list$b0[,2,2]))
  }
  if(i>3){ 
   virtpop[,i,1] <- rbinom(nsim, size=virtpop[,i-1,1], prob=plogis(mod$sims.list$b0[,1,3]))
    virtpop[,i,2] <- rbinom(nsim, size=virtpop[,i-1,2], prob=plogis(mod$sims.list$b0[,2,3]))
  }
}

SurvF <- apply(virtpop[,,1], 2, sum)/nsim
SurvM <- apply(virtpop[,,2], 2, sum)/nsim


plot(0:99, SurvF, type="l", lwd=2, col="orange", xlim=c(0,12), las=1, ylab="Proportion survived", xlab="Age [years]")
lines(0:99, SurvM, lwd=2, col="blue")
legend(5,1, lwd=2, col=c("orange", "blue"),
       legend=c("females", "males "))
```

Figure 2.3: Survivor curve for the two areas per sex

```
# average age
agefun <- function(x) max(c(1:100)[x==1])-0.5
mean(apply(virtpop[,,1], 1, agefun)) # females
```

```
## [1] 1.854
```

```
mean(apply(virtpop[,,2], 1, agefun)) # males
```

```
## [1] 1.58625
```

# 3 Sensitivity to including both L and R individuals

When both L and R individuals are included, survival will be underestimated because if one individual dies there might two “individuals” in the data (an R and an L belonging to the same individual) disappear. However, if we reduce the data to one of L or R individuals, because individuals that die early may be overrepresented among those deleted from the data by such a selection. Therefore, we refitted the model to data that were not reduced. We found that survival estimates were slightly lower for juveniles whereas for the other age classes, survival estimates were similar when the model was fitted to the non-reduced data.

```
load("modelfits/modelfit_luno_combined_ageyearinklLR231022.rda") 


S <- plogis(apply(mod$sims.list$b0, c(2,3), mean))
Slwr <- plogis(apply(mod$sims.list$b0, c(2,3), quantile, probs=0.025))
Supr <- plogis(apply(mod$sims.list$b0, c(2,3), quantile, probs=0.975))

tab <- expand.grid(age=c("First year", "Second year", "Adult"), 
                   sex=c("females", "males"))
tab$S <- as.numeric(t(S))
tab$S.lwr <- as.numeric(t(Slwr))
tab$S.upr <- as.numeric(t(Supr))
kable(tab, dig=2, caption="Average annual survival estimates from the combined model with 95% uncertainty interval fitted to data that were not reduced to L lynxes.")
```

Table 3.1: Average annual survival estimates from the combined model with 95% uncertainty interval fitted to data that were not reduced to L lynxes.

| age | sex | S | S.lwr | S.upr |
| --- | --- | --- | --- | --- |
| First year | females | 0.14 | 0.04 | 0.45 |
| Second year | females | 0.80 | 0.62 | 0.92 |
| Adult | females | 0.75 | 0.66 | 0.83 |
| First year | males | 0.36 | 0.09 | 0.75 |
| Second year | males | 0.74 | 0.52 | 0.91 |
| Adult | males | 0.80 | 0.71 | 0.88 |
