# Supplementary material for: Long‐Term Changes in Survival of Eurasian Lynx in Three Reintroduced Populations in Switzerland
Source: Ecol Evol. 2025 Mar 30;15(4):e71095. doi: 10.1002/ece3.71095 (PMC11955280; doi:10.1002/ece3.71095)
Supplement: Supplementary file 1 — Appendix S1. [file ECE3-15-e71095-s001.zip › Supplementary_material__README_JURA.html]

Documentation and code of the survival model for the Jura population


Code 

- Show All Code
- Hide All Code

# Documentation and code of the survival model for the Jura population

#### Vogt et al.

#### 2024-07-12

```
library(knitr)
library(R2jags)
```

# 1 Data

## 1.1 Picture and telemetry data

```
load("data/dataxJURA.rda")
str(datax)
```

```
## List of 16
##  $ y           : num [1:380, 1:152] 7 1 7 7 7 1 1 1 7 7 ...
##  $ first       : num [1:380] 92 1 108 61 91 1 1 1 59 59 ...
##  $ sex         : num [1:380] 2 1 1 1 2 1 2 2 NA NA ...
##  $ nind        : int 380
##  $ last        : int [1:380] 115 152 121 67 103 152 152 152 152 152 ...
##  $ age         : num [1:380, 1:26] NA 3 NA NA NA 3 3 2 NA NA ...
##  $ monitoringIb: num [1:152] -0.274 -0.274 -0.274 -0.274 -0.274 ...
##  $ monitoringIa: num [1:152] -0.32 -0.32 -0.32 -0.32 -0.32 ...
##  $ oppeffIa    : num [1:152] 0.348 0.348 0.348 0.348 0.348 ...
##  $ oppeffIb    : num [1:152] -1.94 -1.94 -1.94 -1.94 -1.94 ...
##  $ telemetry   : num [1:380, 1:152] 1 2 1 1 1 2 2 2 1 1 ...
##  $ alpha       : num [1:2] 1 1
##  $ nyears      : num 26
##  $ year        : num [1:152] 1 1 2 2 2 2 2 2 3 3 ...
##  $ fr          : num [1:380] 1 1 1 1 1 1 1 1 1 1 ...
##  $ fr1         : num [1:380] 1 1 1 1 1 1 1 1 1 1 ...
```

The data object `datax` contains the following elements:

- `y`: observation matrix with one row per individual and one column per 2-month period. The entries specify the categorised observation (1 = recorded in I a alive, 2 = recovered in I a dead, 3 = recorded in I b alive, 4 = recovered in I b dead, 5 = recorded outside the study area alive, 6 = recovered outside the study area dead, 7 = no seen during the 2-month period)
- `first`: 2-month period of first release or first detection of the individual
- `sex`: sex per individual (1 = female, 2 = male, NA = unknown)
- `nind`: number of individuals in the data
- `last`: last 2-month period per individual that should be included in the data. For most individuals this is the last 2-month period of the study period. For individuals that were translocated to a different study area, it is the 2-month period of translocation (censoring of data at transolcation).
- `age`: matrix with rows corresponding to individuals and columns to lynx years. Values 1 = lynx in its first year, 2 = lynx in its second year, and 3 = older than second year.
- `monitoringIa`: category of monitoring per 2-month (1 = opportunistic, 2 = deterministic) in I a
- `monitoringIb`: category of monitoring per 2-month (1 = opportunistic, 2 = deterministic) in I b
- `oppeffIa`: observation effort per 2-month period in I a. The larger the value the more cameras were deployed in the study area. This variable referst to the opportunistic monitoring, thus it does not include the number of cameras of the deterministic monitoring for which a separate detection probability is estimated.
- `oppeffIb`: observation effort per 2-month period in I b. The larger the value the more cameras were deployed in the study area. This variable referst to the opportunistic monitoring, thus it does not include the number of cameras of the deterministic monitoring for which a separate detection probability is estimated.
- `telemetry`: specificator when lynxes were tagged by a telemetry device (1 = no telemetry device, 2 = with telemetry device)  
  `
- `alpha`: parameter values of prior dirichlet distribution for the proportion of females and males.
- `nyears`: number of years in the study period
- `year`: assignment of monthes to lynx years
- `fr`: frequency of the observation history
- `fr1`: auxiliary variable enabling the use of marginalised model formulation

Graphical display of age categories per individual and year:

```
image(t(datax$age))
```

Figure 1.1: Age categories (darker colour from 1 to 3) for each individual and year.

```
colkey <- c(rainbow(3), "white")
location <- c(1,1,2,2,3,3, NA)

ch <- datax$y[order(datax$first),]


par(mar=c(2,4,0.1,0.1))
plot(seq(1, ncol(datax$y), length=nrow(datax$y)),
    1:nrow(datax$y), type="n", xlab="2-month period", yaxt="n", ylab=NA)
axis(2, at=1:nrow(datax$y), 1:nrow(datax$y), las=1, cex.axis=0.6)
 nocc <- ncol(datax$y)
for(i in 1:nrow(datax$y)){
  points(1:nocc, rep(i, nocc), pch=15, col=colkey[location[ch[i,]]], cex=0.2)
  inddead <- is.element(ch[i,], c(2,4,6,8)); inddead[is.na(inddead)] <- FALSE
  if(sum(inddead, na.rm=TRUE)>0) points(c(1:nocc)[inddead], i, pch=1, cex=0.2)
  }

legend(0, nrow(ch), pch=c(rep(15, 4), 1), col=c(colkey[-4], 1), 
       legend=c("I a",  "I b", "outside", "found dead"), bty="n", cex=0.8)
```

Figure 1.2: Histories of the individuals.

## 1.2 Age at death data

```
load("data/data_ageatdeath_jura.rda")
str(djags)
```

```
## List of 7
##  $ y       : num [1:152] 9 2 3 1 1 1 1 3 1 1 ...
##  $ ageclass: num [1:18] 1 2 3 3 3 3 3 3 3 3 ...
##  $ sex     : num [1:152] NA 2 1 1 2 2 1 NA 1 2 ...
##  $ area    : num [1:152] 1 1 2 1 2 1 2 1 1 1 ...
##  $ nyears  : num 15
##  $ nind    : int 152
##  $ alpha   : num [1:2] 1 1
```

```
hist(djags$y, main=NA, xlab="Age at death (y)")
```

Figure 1.3: Number of individuals found dead per age in years.

The data contain the following elements:

- `y`: age (in years) at which the lynx has been recovered dead
- `ageclass`: classification of the years in age classes (1 = first year, 2 = second year, 3 = older)
- `sex`: sex per individual (1 = female, 2 = male)
- `area`: area (1 = I a, 2 = I b)
- `nyears`: maximum age
- `nind`: number of individuals
- `alpha`: parameter values for dirichlet prior distribution for the proportion of females and males.

# 2 Integrated model for picture, telemetry and age at death data

## 2.1 Data

```
datax <- list(y=datax$y, fr=rep(1,datax$nind), fr1=rep(1, datax$nind), 
              first=datax$first,
              sex=datax$sex,
              #nocc=datax$nocc,
              nind=datax$nind,
              last=datax$last,
              age=datax$age,
              monitoringIV=datax$monitoringIV,
              monitoringIII=datax$monitoringIII, 
              oppeffIII=datax$oppeffIII,
              oppeffIV=datax$oppeffIV,  
              telemetry=datax$telemetry,
              alpha=c(1,1),
              nyears=datax$nyears,
              year=datax$year,
              # age at death data
              yad=djags$y,
              ageclass=djags$ageclass,
              adsex=djags$sex,
              adnyears=djags$nyears,
              adnind=djags$nind,
              area=djags$area)
str(datax)
```

```
## List of 22
##  $ y            : num [1:380, 1:152] 7 1 7 7 7 1 1 1 7 7 ...
##  $ fr           : num [1:380] 1 1 1 1 1 1 1 1 1 1 ...
##  $ fr1          : num [1:380] 1 1 1 1 1 1 1 1 1 1 ...
##  $ first        : num [1:380] 92 1 108 61 91 1 1 1 59 59 ...
##  $ sex          : num [1:380] 2 1 1 1 2 1 2 2 NA NA ...
##  $ nind         : int 380
##  $ last         : int [1:380] 115 152 121 67 103 152 152 152 152 152 ...
##  $ age          : num [1:380, 1:26] NA 3 NA NA NA 3 3 2 NA NA ...
##  $ monitoringIV : NULL
##  $ monitoringIII: NULL
##  $ oppeffIII    : NULL
##  $ oppeffIV     : NULL
##  $ telemetry    : num [1:380, 1:152] 1 2 1 1 1 2 2 2 1 1 ...
##  $ alpha        : num [1:2] 1 1
##  $ nyears       : num 26
##  $ year         : num [1:152] 1 1 2 2 2 2 2 2 3 3 ...
##  $ yad          : num [1:152] 9 2 3 1 1 1 1 3 1 1 ...
##  $ ageclass     : num [1:18] 1 2 3 3 3 3 3 3 3 3 ...
##  $ adsex        : num [1:152] NA 2 1 1 2 2 1 NA 1 2 ...
##  $ adnyears     : num 15
##  $ adnind       : int 152
##  $ area         : num [1:152] 1 1 2 1 2 1 2 1 1 1 ...
```

## 2.2 Model code

```
cat(readLines('jags/Smod_jura_btocombined_ageyearint_ragedep_marginalisation.txt'), sep = '\n')
```

```
## # integrates the multi-state model with the BTO-dead recovery model
## 
## # for the multi-state model for combining camera trap data and lynx found dead
## # data:
## ## y:   (nind x nocc)
## ##  1 = Ia alive, 2 = Ia dead,  
## ##  3 = Ib alive, 4 = Ib dead, 5 = outside alive, 
## ##  6 = outside dead, 7 = not seen
##  
## 
## ## first: month of first capture (marking)
## ## nocc: number of capture occasions (month)
## 
## # predictors:
## ## sex: vector of length nind with values 1, 2 and NA
## ## age: matrix with 3 age classes
## 
## 
## ## latent variables
## #states z:
## ##  1 = Ia alive, 2 = Ia freshly dead, 
## ##  3 = Ib alive, 4 = Ib freshly dead, 5 = outside alive, 
## ##  6 = outside freshly dead, 7 = dead 
## 
## 
## # for the BTO dead recovery model
## 
## # data
## # y= vector with ages at death (1 = death in first year, 2 = death in second year, 3 = death later)
## # ad.sex = vector with sexes (1=female, 2 = male, NA=not identified)
## # ageclass = vector with ageclasses 1:3
## # ad.nyears: maximum age in years
## # ad.nind: number of individuals in data set with age at death
## 
## model{
## 
##   ## transition probabilities
## for(i in 1:nind){
##   for(t in first[i]:(last[i]-1)){
##   ps[1,i,t,1] <- (1-m12[i,t]-m13[i,t])*pow(s1[i,year[t]],1/6)
##   ps[1,i,t,2] <- (1-pow(s1[i,year[t]],1/6))*r1[i,t]
##   ps[1,i,t,3] <- m12[i,t]*pow(s1[i,year[t]],1/6)
##   ps[1,i,t,4] <- 0
##   ps[1,i,t,5] <- m13[i,t]*pow(s1[i,year[t]],1/6)
##   ps[1,i,t,6] <- 0
##   ps[1,i,t,7] <- (1-pow(s1[i,year[t]],1/6))*(1-r1[i,t])
## 
##   ps[2,i,t,1] <- 0
##   ps[2,i,t,2] <- 0
##   ps[2,i,t,3] <- 0
##   ps[2,i,t,4] <- 0
##   ps[2,i,t,5] <- 0
##   ps[2,i,t,6] <- 0
##   ps[2,i,t,7] <- 1
## 
##   ps[3,i,t,1] <- m21[i,t]*pow(s2[i,year[t]],1/6)
##   ps[3,i,t,2] <- 0
##   ps[3,i,t,3] <- (1-m21[i,t]-m23[i,t])*pow(s2[i,year[t]],1/6)
##   ps[3,i,t,4] <- (1-pow(s2[i,year[t]],1/6))*r2[i,t]
##   ps[3,i,t,5] <- m23[i,t]*pow(s2[i,year[t]],1/6)
##   ps[3,i,t,6] <- 0
##   ps[3,i,t,7] <- (1-pow(s2[i,year[t]],1/6))*(1-r2[i,t])
## 
##   ps[4,i,t,1] <- 0
##   ps[4,i,t,2] <- 0
##   ps[4,i,t,3] <- 0
##   ps[4,i,t,4] <- 0
##   ps[4,i,t,5] <- 0
##   ps[4,i,t,6] <- 0
##   ps[4,i,t,7] <- 1
## 
##   ps[5,i,t,1] <- 0
##   ps[5,i,t,2] <- 0
##   ps[5,i,t,3] <- 0
##   ps[5,i,t,4] <- 0
##   ps[5,i,t,5] <- pow(s3[i,year[t]],1/6)
##   ps[5,i,t,6] <- (1-pow(s3[i,year[t]],1/6))*r3[i,t]
##   ps[5,i,t,7] <- (1-pow(s3[i,year[t]],1/6))*(1-r3[i,t])
## 
##   ps[6,i,t,1] <- 0
##   ps[6,i,t,2] <- 0
##   ps[6,i,t,3] <- 0
##   ps[6,i,t,4] <- 0
##   ps[6,i,t,5] <- 0
##   ps[6,i,t,6] <- 0
##   ps[6,i,t,7] <- 1
## 
##   ps[7,i,t,1] <- 0
##   ps[7,i,t,2] <- 0
##   ps[7,i,t,3] <- 0
##   ps[7,i,t,4] <- 0
##   ps[7,i,t,5] <- 0
##   ps[7,i,t,6] <- 0
##   ps[7,i,t,7] <- 1
##   }
##   for(t in first[i]:last[i]){
##   po[1,i,t,1] <- p1[i,t]
##   po[1,i,t,2] <- 0
##   po[1,i,t,3] <- 0
##   po[1,i,t,4] <- 0
##   po[1,i,t,5] <- 0
##   po[1,i,t,6] <- 0
##   po[1,i,t,7] <- 1-p1[i,t]
## 
##   po[2,i,t,1] <- 0
##   po[2,i,t,2] <- 1
##   po[2,i,t,3] <- 0
##   po[2,i,t,4] <- 0
##   po[2,i,t,5] <- 0
##   po[2,i,t,6] <- 0
##   po[2,i,t,7] <- 0
## 
##   po[3,i,t,1] <- 0
##   po[3,i,t,2] <- 0
##   po[3,i,t,3] <- p2[i,t]
##   po[3,i,t,4] <- 0
##   po[3,i,t,5] <- 0
##   po[3,i,t,6] <- 0
##   po[3,i,t,7] <- 1-p2[i,t]
## 
##   po[4,i,t,1] <- 0
##   po[4,i,t,2] <- 0
##   po[4,i,t,3] <- 0
##   po[4,i,t,4] <- 1
##   po[4,i,t,5] <- 0
##   po[4,i,t,6] <- 0
##   po[4,i,t,7] <- 0
## 
##   po[5,i,t,1] <- 0
##   po[5,i,t,2] <- 0
##   po[5,i,t,3] <- 0
##   po[5,i,t,4] <- 0
##   po[5,i,t,5] <- p3[i,t]
##   po[5,i,t,6] <- 0
##   po[5,i,t,7] <- 1-p3[i,t]
## 
##   po[6,i,t,1] <- 0
##   po[6,i,t,2] <- 0
##   po[6,i,t,3] <- 0
##   po[6,i,t,4] <- 0
##   po[6,i,t,5] <- 0
##   po[6,i,t,6] <- 1
##   po[6,i,t,7] <- 0
## 
##   po[7,i,t,1] <- 0
##   po[7,i,t,2] <- 0
##   po[7,i,t,3] <- 0
##   po[7,i,t,4] <- 0
##   po[7,i,t,5] <- 0
##   po[7,i,t,6] <- 0
##   po[7,i,t,7] <- 1
##   } # t
## } # i
## 
##   ## likelihood 
##   for(i in 1:nind){
##     zeta[i,first[i],1]  <- equals(y[i,first[i]],1)
##     zeta[i,first[i],2]  <- 0
##     zeta[i,first[i],3]  <- equals(y[i,first[i]],3)
##     zeta[i,first[i],4]  <- 0
##     zeta[i,first[i],5]  <- equals(y[i,first[i]],5)
##     zeta[i,first[i],6]  <- 0
##     zeta[i,first[i],7]  <- 0
##     for(t in (first[i]+1):last[i]) { 
##       for(j in 1:7){  
##       zeta[i,t,j] <- inprod(zeta[i, t-1,], ps[,i,t-1,j])*po[j,i,t, y[i,t]] 
##       }
##     }
##    lik[i]<- sum(zeta[i,last[i],]) # sum the likelihood over all states (should have one number only)
##    fr[i] ~ dbin(lik[i],fr1[i])  # fr and fr1 are the same but with different names, number of observed capture histories (can be ones only)
##   }
## 
##   ## linear predictors
##   for(i in 1:nind){
##     ## impute unknown sexes
##     sex[i] ~ dcat(propsex)
##     for(v in year[first[i]]:nyears){
##       logit(s1[i,v]) <-  b10[sex[i], age[i,v]] + sigmaS1year*yearS1eff[v] 
##       logit(s2[i,v]) <-  b20[sex[i], age[i,v]] + sigmaS2year*yearS2eff[v] 
##       logit(s3[i,v]) <-  b30
##     }
##     for(t in first[i]:last[i]){
##       # hier multi-variate logit-link funktion, um Veränderung über die Jahre einzufügen
##       m12[i,t] <- m012[sex[i], age[i,year[t]]]
##       m21[i,t] <- m021[sex[i], age[i,year[t]]]
##       m23[i,t] <- m023[sex[i], age[i,year[t]]]
##       m13[i,t] <- m013[sex[i], age[i,year[t]]]
##       logit(p1[i,t]) <- a10[sex[i], age[i,year[t]],  telemetry[i,t]] + a1*monitoringIa[t] + a2*oppeffIa[t] + sigmapind*indeff[i]  #  
##       logit(p2[i,t]) <- a20[sex[i], age[i,year[t]],  telemetry[i,t]] + a1*monitoringIb[t]  + a2*oppeffIb[t] + sigmapind*indeff[i]  # 
##       logit(p3[i,t]) <- a30[sex[i], age[i,year[t]],  telemetry[i,t]]   # 
##      
##       logit(r1[i,t]) <-  d0[sex[i], age[i,year[t]]]
##       logit(r2[i,t]) <-  d0[sex[i], age[i,year[t]]] + d11
##       logit(r3[i,t]) <-  d0[sex[i], age[i,year[t]]] + d12
##   
##     }  
##   }
## 
##   ## priors
##   for(i in 1:nind){
##     indeff[i]~dnorm(0,1)
##   }
##   sigmapind ~ dt(0,1,2)I(0,)
## 
##   for(i in 1:nyears){
##     yearS1eff[i]~dnorm(0,1)
##     yearS2eff[i]~dnorm(0,1)
##   }
##   sigmaS1year ~ dt(0,1,2)I(0,)
##   sigmaS2year ~ dt(0,1,2)I(0,)
## 
##   propsex[1:2] ~ ddirch(alpha[1:2])
## 
## # prior für survival # = Normal(mean,1.5) for intercept: mean corresponds to logit(average survival) 
##   # for that age class, to prevent prior influence on among-year variance that is due to different sample sizes
##   b10[1,1] ~ dnorm(0, 0.44)  # juvenile females 0.55 in Ia, 0.46 in Ib - > average 0.5
##   b10[2,1] ~ dnorm(0, 0.44)  # juvenile males 0.24 in Ia, 0.20 in Ib - > average 0.22 = inv-logit(-1.2)
##   b10[1,2] ~ dnorm(0, 0.44) # subabdult females 0.92 in Ia, 0.69 in Ib - > average 0.8 = inv-logit(1.4)
##   b10[2,2] ~ dnorm(0, 0.44) # subabdult males 0.81 in Ia, 0.55 in Ib - > average 0.68 = inv-logit(0.75)
##   b10[1,3] ~ dnorm(0, 0.44) # abdult females 0.86 in Ia, 0.71 in Ib - > average 0.78 = inv-logit(1.2)
##   b10[2,3] ~ dnorm(0, 0.44) # abdult males 0.79 in Ia, 0.68 in Ib - > average 0.74 = inv-logit(1)
## 
##   b20[1,1] ~ dnorm(0, 0.44)  
##   b20[2,1] ~ dnorm(0, 0.44)
##   b20[1,2] ~ dnorm(0, 0.44)
##   b20[2,2] ~ dnorm(0, 0.44)
##   b20[1,3] ~ dnorm(0, 0.44)
##   b20[2,3] ~ dnorm(0, 0.44)
## 
##   b30 ~ dnorm(0, 0.044)
##   
##   a10[1,1,1] ~ dnorm(0, 0.44) 
##   a10[2,1,1] ~ dnorm(0, 0.44)
##   a10[1,2,1] ~ dnorm(0, 0.44)
##   a10[2,2,1] ~ dnorm(0, 0.44)
##   a10[1,3,1] ~ dnorm(0, 0.44)
##   a10[2,3,1] ~ dnorm(0, 0.44)
## 
##   a10[1,1,2] ~ dnorm(2.5, 4) #in 9 von 120 Monaten wurden telemetrierte nicht registriert  
##   a10[2,1,2] <- a10[1,1,2]     # telemetrierte Individuen haben alle die gleichen Entdeckungswahrscheinlichkeit
##   a10[1,2,2] <- a10[1,1,2]
##   a10[2,2,2] <- a10[1,1,2]
##   a10[1,3,2] <- a10[1,1,2]
##   a10[2,3,2] <- a10[1,1,2]
## 
##   a20[1,1,1] ~ dnorm(0, 0.44)  # = Normal(0,1.5) für Intercept 
##   a20[2,1,1] ~ dnorm(0, 0.44)
##   a20[1,2,1] ~ dnorm(0, 0.44)
##   a20[2,2,1] ~ dnorm(0, 0.44)
##   a20[1,3,1] ~ dnorm(0, 0.44)
##   a20[2,3,1] ~ dnorm(0, 0.44)
## 
##   a20[1,1,2] <- a10[1,1,2]  #  
##   a20[2,1,2] <- a20[1,1,2]     # telemetrierte Individuen haben alle die gleichen Entdeckungswahrscheinlichkeit
##   a20[1,2,2] <- a20[1,1,2]
##   a20[2,2,2] <- a20[1,1,2]
##   a20[1,3,2] <- a20[1,1,2]
##   a20[2,3,2] <- a20[1,1,2]
## 
##   a30[1,1,1] ~ dnorm(0, 0.44)  # = Normal(0,1.5) für Intercept 
##   a30[2,1,1] ~ dnorm(0, 0.44)
##   a30[1,2,1] ~ dnorm(0, 0.44)
##   a30[2,2,1] ~ dnorm(0, 0.44)
##   a30[1,3,1] ~ dnorm(0, 0.44)
##   a30[2,3,1] ~ dnorm(0, 0.44)
## 
##   a30[1,1,2] <- a10[1,1,2]  # 
##   a30[2,1,2] <- a30[1,1,2]     # telemetrierte Individuen haben alle die gleichen Entdeckungswahrscheinlichkeit
##   a30[1,2,2] <- a30[1,1,2]
##   a30[2,2,2] <- a30[1,1,2]
##   a30[1,3,2] <- a30[1,1,2]
##   a30[2,3,2] <- a30[1,1,2]
## 
##   a1 ~ dnorm(0, 0.04) 
##   a2 ~ dnorm(0, 0.04) 
##  
##   d0[1,1] ~ dnorm(0, 0.44)
##   d0[2,1] ~ dnorm(0, 0.44)
##   d0[1,2] ~ dnorm(0, 0.44)
##   d0[2,2] ~ dnorm(0, 0.44)
##   d0[1,3] ~ dnorm(0, 0.44)
##   d0[2,3] ~ dnorm(0, 0.44)
##   
##   d11 ~ dnorm(0, 0.04)
##   d12 ~ dnorm(0, 0.04)
## 
##   m012[1,1] ~ dbeta(1,1)
##   m012[2,1] ~ dbeta(1,1)
##   m012[1,2] ~ dbeta(1,1)
##   m012[2,2] ~ dbeta(1,1)
##   m012[1,3] <-  m012[1,2]
##   m012[2,3] <-  m012[2,2]
##  
##   u0[1,1] <- 1- m012[1,1]
##   u0[2,1] <- 1- m012[2,1]
##   u0[1,2] <- 1- m012[1,2]
##   u0[2,2] <- 1- m012[2,2]
##   u0[1,3] <- 1- m012[1,3]
##   u0[2,3] <- 1- m012[2,3]
##   
##   m013[1,1] ~ dunif(0,u0[1,1])
##   m013[2,1] ~ dunif(0,u0[2,1])
##   m013[1,2] ~ dunif(0,u0[1,2])
##   m013[2,2] ~ dunif(0,u0[2,2])
##   m013[1,3] <-  m013[1,2]
##   m013[2,3] <-  m013[2,2]
## 
##   m021[1,1] ~ dbeta(1,1)
##   m021[2,1] ~ dbeta(1,1)
##   m021[1,2] ~ dbeta(1,1)
##   m021[2,2] ~ dbeta(1,1)
##   m021[1,3] <-  m021[1,2]
##   m021[2,3] <-  m021[2,2]
##  
##   u20[1,1] <- 1- m021[1,1]
##   u20[2,1] <- 1- m021[2,1]
##   u20[1,2] <- 1- m021[1,2]
##   u20[2,2] <- 1- m021[2,2]
##   u20[1,3] <- 1- m021[1,3]
##   u20[2,3] <- 1- m021[2,3]
##   
##   m023[1,1] ~ dunif(0,u20[1,1])
##   m023[2,1] ~ dunif(0,u20[2,1])
##   m023[1,2] ~ dunif(0,u20[1,2])
##   m023[2,2] ~ dunif(0,u20[2,2])
##   m023[1,3] <-   m023[1,2]
##   m023[2,3] <-   m023[2,2]
## 
## 
## # model for age at death
##   for(s in 1:2){
##     for(a in 1:3){
##       logit(rsa[s,a,1]) <- d0[s,a] # get recovery probabilty per sex and ageclass 
##       logit(rsa[s,a,2]) <- d0[s,a] + d11 # get recovery probabilty per sex and ageclass 
##       logit(Ssa[s,a, 1]) <- b10[s,a] # get survival per sex, ageclass and area
##       logit(Ssa[s,a, 2]) <- b20[s,a] # get survival per sex, ageclass and area  
##     }
##   # propabiliy of beeing dying within 18 years and beeing found
##  for(aa in 1:2){
##   pfounddead[s, aa]  <- (1-Ssa[s,1,aa])*rsa[s,1,aa] + Ssa[s,1,aa]*(1-Ssa[s,2,aa])*rsa[s,2,aa] + 
##                         Ssa[s,1,aa]*Ssa[s,2,aa]*(1-Ssa[s,3,aa])*rsa[s,3,aa]*(pow(Ssa[s,3, aa],18-2)-1)/(Ssa[s,3, aa]-1)
##    }
##   }
## 
## # likelihood
##   for(i in 1:adnind){
##     yad[i] ~ dcat(adp[i,1:adnyears]) # y[i]: year of dead recovery of individual i
##     
##     adp[i,1] <- (1-adS[i,1])*adr[i, ageclass[1]]/pfounddead[adsex[i], area[i]]
##     adsex[i] ~ dcat(propsex)
##     for(j in 2:adnyears){ 
##       adp[i,j] <- prod(adS[i,1:(j-1)])*(1-adS[i,j])*adr[i, ageclass[j]]/pfounddead[adsex[i], area[i]]
##     }
##     for(j in 1:adnyears){
##       adS[i,j] <- Ssa[adsex[i],ageclass[j], area[i]] # same intercept as in multi-state model
##       adr[i,j] <- rsa[adsex[i],ageclass[j], area[i]]  
##     }
##   }
## }
```

```
# with interaction age x year for survival
mod <- jags.parallel(datax, inits=initfun, parameters.to.save=c("a10", "a20",  "a30", "a1", "b10", "b20","b30",  "d0", "m012", "m021", "m023", "m013", "sigmapind", "indeff", "sigmaS1year", "yearS1eff", "sigmaS2year", "yearS2eff", "propsex", "sex"),
            model.file="jags/Smod_jura_btocombined_ageyearint_ragedep_marginalisation.txt", n.chains=3, n.iter=20000, n.thin=3, n.burnin=5000)


mod <- mod$BUGSoutput
save(mod, file="modelfits/modelfit_jura_combined_ageyear231122.rda") 

# check convergence
 # plot(mod$sims.array[,1,1], type="l")
 # lines(mod$sims.array[,2,1], col=2)
 # lines(mod$sims.array[,3,1], col=3)

  # plot(mod$sims.array[,1,10], type="l")
  # lines(mod$sims.array[,2,10], col=2)
  # lines(mod$sims.array[,3,10], col=3)
```

## 2.3 Results

```
load("modelfits/modelfit_jura_combined_ageyear240610.rda") # m per sex and age

tab <- mod$summary[c(1:60, 441:469),]
kable(tab, dig=2, caption="Statistics of the model estimates including convergence assessment statistics. For interpretation of parameters, see first table")
```

Table 2.1: Statistics of the model estimates including convergence assessment statistics. For interpretation of parameters, see first table


|  | mean | sd | 2.5% | 25% | 50% | 75% | 97.5% | Rhat | n.eff |
| --- | --- | --- | --- | --- | --- | --- | --- | --- | --- |
| a1 | 0.17 | 0.03 | 0.11 | 0.15 | 0.17 | 0.20 | 0.24 | 1 | 15000 |
| a10[1,1,1] | -0.27 | 0.52 | -1.25 | -0.62 | -0.28 | 0.08 | 0.80 | 1 | 10000 |
| a10[2,1,1] | -0.49 | 0.49 | -1.44 | -0.82 | -0.49 | -0.17 | 0.47 | 1 | 15000 |
| a10[1,2,1] | -1.92 | 0.44 | -2.84 | -2.19 | -1.89 | -1.62 | -1.12 | 1 | 9800 |
| a10[2,2,1] | -2.36 | 0.49 | -3.27 | -2.70 | -2.38 | -2.04 | -1.36 | 1 | 2200 |
| a10[1,3,1] | -2.14 | 0.23 | -2.60 | -2.29 | -2.14 | -1.99 | -1.69 | 1 | 9800 |
| a10[2,3,1] | -1.65 | 0.25 | -2.13 | -1.81 | -1.64 | -1.48 | -1.16 | 1 | 1900 |
| a10[1,1,2] | 2.83 | 0.36 | 2.15 | 2.58 | 2.82 | 3.06 | 3.55 | 1 | 15000 |
| a10[2,1,2] | 2.83 | 0.36 | 2.15 | 2.58 | 2.82 | 3.06 | 3.55 | 1 | 15000 |
| a10[1,2,2] | 2.83 | 0.36 | 2.15 | 2.58 | 2.82 | 3.06 | 3.55 | 1 | 15000 |
| a10[2,2,2] | 2.83 | 0.36 | 2.15 | 2.58 | 2.82 | 3.06 | 3.55 | 1 | 15000 |
| a10[1,3,2] | 2.83 | 0.36 | 2.15 | 2.58 | 2.82 | 3.06 | 3.55 | 1 | 15000 |
| a10[2,3,2] | 2.83 | 0.36 | 2.15 | 2.58 | 2.82 | 3.06 | 3.55 | 1 | 15000 |
| a2 | 0.58 | 0.14 | 0.30 | 0.48 | 0.58 | 0.68 | 0.85 | 1 | 2100 |
| a20[1,1,1] | -1.29 | 0.39 | -2.06 | -1.55 | -1.28 | -1.02 | -0.52 | 1 | 12000 |
| a20[2,1,1] | -0.31 | 0.49 | -1.24 | -0.64 | -0.32 | 0.00 | 0.67 | 1 | 7800 |
| a20[1,2,1] | -1.78 | 0.31 | -2.40 | -1.99 | -1.78 | -1.57 | -1.18 | 1 | 15000 |
| a20[2,2,1] | 0.03 | 0.53 | -0.94 | -0.33 | 0.01 | 0.38 | 1.14 | 1 | 2800 |
| a20[1,3,1] | -1.44 | 0.27 | -1.98 | -1.62 | -1.44 | -1.26 | -0.94 | 1 | 2600 |
| a20[2,3,1] | -0.43 | 0.23 | -0.88 | -0.58 | -0.43 | -0.28 | 0.02 | 1 | 2500 |
| a20[1,1,2] | 2.83 | 0.36 | 2.15 | 2.58 | 2.82 | 3.06 | 3.55 | 1 | 15000 |
| a20[2,1,2] | 2.83 | 0.36 | 2.15 | 2.58 | 2.82 | 3.06 | 3.55 | 1 | 15000 |
| a20[1,2,2] | 2.83 | 0.36 | 2.15 | 2.58 | 2.82 | 3.06 | 3.55 | 1 | 15000 |
| a20[2,2,2] | 2.83 | 0.36 | 2.15 | 2.58 | 2.82 | 3.06 | 3.55 | 1 | 15000 |
| a20[1,3,2] | 2.83 | 0.36 | 2.15 | 2.58 | 2.82 | 3.06 | 3.55 | 1 | 15000 |
| a20[2,3,2] | 2.83 | 0.36 | 2.15 | 2.58 | 2.82 | 3.06 | 3.55 | 1 | 15000 |
| a30[1,1,1] | -1.91 | 1.37 | -4.26 | -2.82 | -2.03 | -1.15 | 1.31 | 1 | 4500 |
| a30[2,1,1] | -1.68 | 1.55 | -4.30 | -2.74 | -1.86 | -0.79 | 1.85 | 1 | 15000 |
| a30[1,2,1] | -2.80 | 0.92 | -4.66 | -3.38 | -2.79 | -2.22 | -1.05 | 1 | 3700 |
| a30[2,2,1] | -1.39 | 0.83 | -2.86 | -1.95 | -1.44 | -0.88 | 0.38 | 1 | 15000 |
| a30[1,3,1] | -1.96 | 1.16 | -4.08 | -2.79 | -2.02 | -1.18 | 0.40 | 1 | 6500 |
| a30[2,3,1] | -2.93 | 0.41 | -3.74 | -3.20 | -2.93 | -2.66 | -2.14 | 1 | 7600 |
| a30[1,1,2] | 2.83 | 0.36 | 2.15 | 2.58 | 2.82 | 3.06 | 3.55 | 1 | 15000 |
| a30[2,1,2] | 2.83 | 0.36 | 2.15 | 2.58 | 2.82 | 3.06 | 3.55 | 1 | 15000 |
| a30[1,2,2] | 2.83 | 0.36 | 2.15 | 2.58 | 2.82 | 3.06 | 3.55 | 1 | 15000 |
| a30[2,2,2] | 2.83 | 0.36 | 2.15 | 2.58 | 2.82 | 3.06 | 3.55 | 1 | 15000 |
| a30[1,3,2] | 2.83 | 0.36 | 2.15 | 2.58 | 2.82 | 3.06 | 3.55 | 1 | 15000 |
| a30[2,3,2] | 2.83 | 0.36 | 2.15 | 2.58 | 2.82 | 3.06 | 3.55 | 1 | 15000 |
| b10[1,1] | 0.37 | 0.67 | -0.88 | -0.10 | 0.34 | 0.81 | 1.73 | 1 | 2300 |
| b10[2,1] | -1.19 | 0.53 | -2.16 | -1.55 | -1.21 | -0.86 | -0.05 | 1 | 3400 |
| b10[1,2] | 2.31 | 0.68 | 1.05 | 1.83 | 2.28 | 2.75 | 3.69 | 1 | 15000 |
| b10[2,2] | 1.74 | 0.65 | 0.48 | 1.30 | 1.73 | 2.18 | 3.01 | 1 | 12000 |
| b10[1,3] | 1.73 | 0.25 | 1.30 | 1.56 | 1.72 | 1.89 | 2.26 | 1 | 15000 |
| b10[2,3] | 1.40 | 0.29 | 0.89 | 1.20 | 1.38 | 1.58 | 2.03 | 1 | 3000 |
| b20[1,1] | 0.04 | 0.64 | -1.13 | -0.41 | 0.01 | 0.46 | 1.39 | 1 | 3800 |
| b20[2,1] | -1.56 | 0.57 | -2.57 | -1.94 | -1.59 | -1.22 | -0.34 | 1 | 15000 |
| b20[1,2] | 0.79 | 0.54 | -0.20 | 0.41 | 0.76 | 1.14 | 1.90 | 1 | 8800 |
| b20[2,2] | 0.39 | 0.56 | -0.67 | 0.00 | 0.38 | 0.76 | 1.49 | 1 | 15000 |
| b20[1,3] | 0.87 | 0.26 | 0.37 | 0.70 | 0.87 | 1.05 | 1.41 | 1 | 2200 |
| b20[2,3] | 0.90 | 0.29 | 0.36 | 0.70 | 0.89 | 1.09 | 1.52 | 1 | 7500 |
| b30 | -0.48 | 0.43 | -1.36 | -0.76 | -0.47 | -0.19 | 0.34 | 1 | 15000 |
| d0[1,1] | -1.12 | 0.88 | -2.55 | -1.72 | -1.23 | -0.65 | 0.96 | 1 | 8100 |
| d0[2,1] | -2.83 | 0.55 | -3.82 | -3.20 | -2.84 | -2.48 | -1.69 | 1 | 2300 |
| d0[1,2] | -0.92 | 0.77 | -2.26 | -1.46 | -0.99 | -0.46 | 0.79 | 1 | 5200 |
| d0[2,2] | -0.25 | 0.94 | -1.80 | -0.91 | -0.37 | 0.29 | 1.88 | 1 | 7700 |
| d0[1,3] | -1.54 | 0.33 | -2.21 | -1.76 | -1.55 | -1.32 | -0.88 | 1 | 1400 |
| d0[2,3] | -1.28 | 0.37 | -1.99 | -1.53 | -1.29 | -1.03 | -0.52 | 1 | 3400 |
| d11 | -0.20 | 0.38 | -0.94 | -0.45 | -0.19 | 0.06 | 0.53 | 1 | 1900 |
| d12 | -2.50 | 0.95 | -4.56 | -3.08 | -2.44 | -1.84 | -0.82 | 1 | 11000 |
| deviance | 7387.26 | 33.31 | 7323.13 | 7364.71 | 7387.12 | 7409.79 | 7453.13 | 1 | 3900 |
| m012[1,1] | 0.10 | 0.05 | 0.02 | 0.06 | 0.09 | 0.13 | 0.21 | 1 | 15000 |
| m012[2,1] | 0.06 | 0.03 | 0.01 | 0.03 | 0.05 | 0.08 | 0.14 | 1 | 15000 |
| m012[1,2] | 0.00 | 0.00 | 0.00 | 0.00 | 0.00 | 0.00 | 0.01 | 1 | 15000 |
| m012[2,2] | 0.03 | 0.01 | 0.02 | 0.03 | 0.03 | 0.04 | 0.05 | 1 | 15000 |
| m012[1,3] | 0.00 | 0.00 | 0.00 | 0.00 | 0.00 | 0.00 | 0.01 | 1 | 15000 |
| m012[2,3] | 0.03 | 0.01 | 0.02 | 0.03 | 0.03 | 0.04 | 0.05 | 1 | 15000 |
| m013[1,1] | 0.09 | 0.06 | 0.01 | 0.04 | 0.07 | 0.12 | 0.24 | 1 | 3200 |
| m013[2,1] | 0.05 | 0.05 | 0.00 | 0.01 | 0.03 | 0.07 | 0.19 | 1 | 8600 |
| m013[1,2] | 0.00 | 0.00 | 0.00 | 0.00 | 0.00 | 0.00 | 0.01 | 1 | 15000 |
| m013[2,2] | 0.03 | 0.01 | 0.01 | 0.02 | 0.03 | 0.04 | 0.06 | 1 | 4800 |
| m013[1,3] | 0.00 | 0.00 | 0.00 | 0.00 | 0.00 | 0.00 | 0.01 | 1 | 15000 |
| m013[2,3] | 0.03 | 0.01 | 0.01 | 0.02 | 0.03 | 0.04 | 0.06 | 1 | 4800 |
| m021[1,1] | 0.02 | 0.02 | 0.00 | 0.01 | 0.01 | 0.03 | 0.06 | 1 | 15000 |
| m021[2,1] | 0.07 | 0.05 | 0.01 | 0.04 | 0.07 | 0.10 | 0.19 | 1 | 15000 |
| m021[1,2] | 0.00 | 0.00 | 0.00 | 0.00 | 0.00 | 0.01 | 0.01 | 1 | 15000 |
| m021[2,2] | 0.02 | 0.01 | 0.01 | 0.02 | 0.02 | 0.03 | 0.04 | 1 | 15000 |
| m021[1,3] | 0.00 | 0.00 | 0.00 | 0.00 | 0.00 | 0.01 | 0.01 | 1 | 15000 |
| m021[2,3] | 0.02 | 0.01 | 0.01 | 0.02 | 0.02 | 0.03 | 0.04 | 1 | 15000 |
| m023[1,1] | 0.04 | 0.03 | 0.00 | 0.01 | 0.03 | 0.05 | 0.12 | 1 | 15000 |
| m023[2,1] | 0.07 | 0.06 | 0.00 | 0.02 | 0.05 | 0.10 | 0.24 | 1 | 15000 |
| m023[1,2] | 0.00 | 0.00 | 0.00 | 0.00 | 0.00 | 0.01 | 0.02 | 1 | 13000 |
| m023[2,2] | 0.02 | 0.01 | 0.01 | 0.02 | 0.02 | 0.03 | 0.04 | 1 | 14000 |
| m023[1,3] | 0.00 | 0.00 | 0.00 | 0.00 | 0.00 | 0.01 | 0.02 | 1 | 13000 |
| m023[2,3] | 0.02 | 0.01 | 0.01 | 0.02 | 0.02 | 0.03 | 0.04 | 1 | 14000 |
| propsex[1] | 0.47 | 0.03 | 0.41 | 0.45 | 0.47 | 0.49 | 0.52 | 1 | 15000 |
| propsex[2] | 0.53 | 0.03 | 0.48 | 0.51 | 0.53 | 0.55 | 0.59 | 1 | 15000 |
| sigmaS1year | 0.79 | 0.40 | 0.06 | 0.51 | 0.80 | 1.06 | 1.60 | 1 | 3200 |
| sigmaS2year | 0.79 | 0.31 | 0.25 | 0.58 | 0.76 | 0.97 | 1.47 | 1 | 2800 |
| sigmapind | 1.21 | 0.11 | 1.01 | 1.13 | 1.20 | 1.28 | 1.45 | 1 | 800 |

```
S1 <- plogis(apply(mod$sims.list$b10, c(2,3), mean))
S1lwr <- plogis(apply(mod$sims.list$b10, c(2,3), quantile, probs=0.025))
S1upr <- plogis(apply(mod$sims.list$b10, c(2,3), quantile, probs=0.975))
 
S2 <- plogis(apply(mod$sims.list$b20, c(2,3), mean))
S2lwr <- plogis(apply(mod$sims.list$b20, c(2,3), quantile, probs=0.025))
S2upr <- plogis(apply(mod$sims.list$b20, c(2,3), quantile, probs=0.975))

  
plot(1:3, seq(0,1, length=3), type="n", las=1, xaxt="n", xlab="", ylim=c(0,1), ylab="Annual survival", xlim=c(0.5, 3.5))
#abline(h=0.5, col=grey(0.8), lwd=2) # prior mean
# females
segments(c(1:3)+0.1, S1lwr[1,], c(1:3)+0.1, S1upr[1,],lwd=2, lend="butt", col="orange")
points(c(1:3)+0.1, S1[1,], pch=21, col="orange", bg="white")
# males
segments(c(1:3)-0.1, S1lwr[2,], c(1:3)-0.1, S1upr[2,],lwd=2, lend="butt", col="blue")
points(c(1:3)-0.1, S1[2,], pch=21, col="blue", bg="white")


segments(c(1:3)+0.19, S2lwr[1,], c(1:3)+0.19, S2upr[1,],lwd=2, lend="butt", col="orange")
points(c(1:3)+0.19, S2[1,], pch=16, col="orange")
# males
segments(c(1:3)-0.01, S2lwr[2,], c(1:3)-0.01, S2upr[2,],lwd=2, lend="butt", col="blue")
points(c(1:3)-0.01, S2[2,], pch=16, col="blue")


axis(1, at=1:3, labels=c("age 1", "age 2", "age 3"))
legend(0.5, 1.25, xpd=NA, lwd=2, col=c("orange", "blue", "orange", "blue"),
       pch=c(21,21,16,16), pt.bg="white", legend=c("females I a", "males I a", "females I b", "males I b"), ncol=2, bty="n")
```

Figure 2.1: Estimated average annual apparent survival probability per age class based on combined data. Vertical bars are 95% compatibility intervals.

```
tab <- expand.grid(age=c("juveniles", "subadult", "adult"), 
                   sex=c("females", "males"))
tab$Ia_S <- as.numeric(t(S1))
tab$Ia_S.lwr <- as.numeric(t(S1lwr))
tab$Ia_S.upr <- as.numeric(t(S1upr))
tab$Ib_S <- as.numeric(t(S2))
tab$Ib_S.lwr <- as.numeric(t(S2lwr))
tab$Ib_S.upr <- as.numeric(t(S2upr))
kable(tab, dig=2, caption="Average annual survival estimates from the combined model with 95% uncertainty interval.")
```

Table 2.2: Average annual survival estimates from the combined model with 95% uncertainty interval.


| age | sex | Ia\_S | Ia\_S.lwr | Ia\_S.upr | Ib\_S | Ib\_S.lwr | Ib\_S.upr |
| --- | --- | --- | --- | --- | --- | --- | --- |
| juveniles | females | 0.59 | 0.29 | 0.85 | 0.51 | 0.24 | 0.80 |
| subadult | females | 0.91 | 0.74 | 0.98 | 0.69 | 0.45 | 0.87 |
| adult | females | 0.85 | 0.79 | 0.91 | 0.71 | 0.59 | 0.80 |
| juveniles | males | 0.23 | 0.10 | 0.49 | 0.17 | 0.07 | 0.41 |
| subadult | males | 0.85 | 0.62 | 0.95 | 0.60 | 0.34 | 0.82 |
| adult | males | 0.80 | 0.71 | 0.88 | 0.71 | 0.59 | 0.82 |

```
nsim <- mod$n.sims

S1pyear <- array(NA, dim=c(2,3, datax$nyears, mod$n.sims))
for(i in 1:2){
  for(j in 1:3){
    S1pyear[i,j,,] <- matrix(mod$sims.list$b10[,i,j], ncol=mod$n.sims, nrow=datax$nyears, byrow=TRUE)+ matrix(mod$sims.list$sigmaS1year, ncol=mod$n.sims, nrow=datax$nyears, byrow=TRUE)*t(mod$sims.list$yearS1eff[,1:datax$nyears])
  }
}


S2pyear <- array(NA, dim=c(2,3, datax$nyears, mod$n.sims))
for(i in 1:2){
  for(j in 1:3){
    S2pyear[i,j,,] <- matrix(mod$sims.list$b20[,i,j], ncol=mod$n.sims, nrow=datax$nyears, byrow=TRUE)+ matrix(mod$sims.list$sigmaS2year, ncol=mod$n.sims, nrow=datax$nyears, byrow=TRUE)*t(mod$sims.list$yearS2eff[,1:datax$nyears])
  }
}

S1pyearm <- plogis(apply(S1pyear, c(1,2,3), mean))
S1pyearlwr <- plogis(apply(S1pyear, c(1,2,3), quantile, probs=0.025))
S1pyearupr <- plogis(apply(S1pyear, c(1,2,3), quantile, probs=0.975))

S2pyearm <- plogis(apply(S2pyear, c(1,2,3), mean))
S2pyearlwr <- plogis(apply(S2pyear, c(1,2,3), quantile, probs=0.025))
S2pyearupr <- plogis(apply(S2pyear, c(1,2,3), quantile, probs=0.975))


par(mfrow=c(3,1), mar=c(0.5, 3,1.5, 0.5), oma=c(3,3,0.6,0))
plot(1:datax$nyears, seq(0,1, length=datax$nyears), type="n", las=1, xaxt="n", xlab="", ylim=c(0,1), ylab="Annual survival", xlim=c(1,datax$nyears))
mtext("a)", adj=0, side=3) # adults


legend(14, 1.26, xpd=NA, col=c("orange", "blue", "brown", "lightblue"), pch=c(21,21,16,16), bg="white", legend=c("females I a", "males I a", "females I b", "males I b"), ncol=2, bty="n")


segments(c(1:datax$nyears), S1pyearlwr[1,3,], c(1:datax$nyears), S1pyearupr[1,3,],lwd=2, lend="butt", col="orange")
points(c(1:datax$nyears), S1pyearm[1,3,], pch=21, col="orange", bg="white")

segments(c(1:datax$nyears)-0.15, S1pyearlwr[2,3,], c(1:datax$nyears)-0.15, S1pyearupr[2,3,],lwd=2, lend="butt", col="blue")
points(c(1:datax$nyears)-0.15, S1pyearm[2,3,], pch=21, col="blue", bg="white")


segments(c(1:datax$nyears)+0.2, S2pyearlwr[1,3,], c(1:datax$nyears)+0.2, S2pyearupr[1,3,],lwd=2, lend="butt", col="brown")
points(c(1:datax$nyears)+0.2, S2pyearm[1,3,], pch=16, col="brown")

segments(c(1:datax$nyears)+0.3, S2pyearlwr[2,3,], c(1:datax$nyears)+0.3, S2pyearupr[2,3,],lwd=2, lend="butt", col="lightblue")
points(c(1:datax$nyears)+0.3, S2pyearm[2,3,], pch=16, col="lightblue")

plot(1:datax$nyears, seq(0,1, length=datax$nyears), type="n", las=1, xaxt="n", xlab="", ylim=c(0,1), ylab="Annual survival", xlim=c(1,datax$nyears))

segments(c(1:datax$nyears), S1pyearlwr[1,2,], c(1:datax$nyears), S1pyearupr[1,2,],lwd=2, lend="butt", col="orange")
points(c(1:datax$nyears), S1pyearm[1,2,], pch=21, col="orange", bg="white")

segments(c(1:datax$nyears)-0.15, S1pyearlwr[2,2,], c(1:datax$nyears)-0.15, S1pyearupr[2,2,],lwd=2, lend="butt", col="blue")
points(c(1:datax$nyears)-0.15, S1pyearm[2,2,], pch=21, col="blue", bg="white")


segments(c(1:datax$nyears)+0.2, S2pyearlwr[1,2,], c(1:datax$nyears)+0.2, S2pyearupr[1,2,],lwd=2, lend="butt", col="brown")
points(c(1:datax$nyears)+0.2, S2pyearm[1,2,], pch=16, col="brown")

segments(c(1:datax$nyears)+0.35, S2pyearlwr[2,2,], c(1:datax$nyears)+0.35, S2pyearupr[2,2,],lwd=2, lend="butt", col="lightblue")
points(c(1:datax$nyears)+0.35, S2pyearm[2,2,], pch=16, col="lightblue")
mtext("b)", adj=0, side=3) # second year


plot(1:datax$nyears, seq(0,1, length=datax$nyears), type="n", las=1, xaxt="n", xlab="", ylim=c(0,1), ylab="Annual survival", xlim=c(1,datax$nyears))

segments(c(1:datax$nyears), S1pyearlwr[1,1,], c(1:datax$nyears), S1pyearupr[1,1,],lwd=2, lend="butt", col="orange")
points(c(1:datax$nyears), S1pyearm[1,1,], pch=21, col="orange", bg="white")

segments(c(1:datax$nyears)-0.15, S1pyearlwr[2,1,], c(1:datax$nyears)-0.15, S1pyearupr[2,1,],lwd=2, lend="butt", col="blue")
points(c(1:datax$nyears)-0.15, S1pyearm[2,1,], pch=21, col="blue", bg="white")


segments(c(1:datax$nyears)+0.2, S2pyearlwr[1,1,], c(1:datax$nyears)+0.2, S2pyearupr[1,1,],lwd=2, lend="butt", col="brown")
points(c(1:datax$nyears)+0.2, S2pyearm[1,1,], pch=16, col="brown")

segments(c(1:datax$nyears)+0.35, S2pyearlwr[2,1,], c(1:datax$nyears)+0.35, S2pyearupr[2,1,],lwd=2, lend="butt", col="lightblue")
points(c(1:datax$nyears)+0.35, S2pyearm[2,1,], pch=16, col="lightblue")

mtext("c)", adj=0, side=3) # first year
mtext("Annual survival", side=2, outer=TRUE, line=0.5)

axis(1, at=1:datax$nyears, labels=1997:c(1997+(datax$nyears-1)))
```

Figure 2.2: Estimated annual apparent survival probability for adults based on combined data. Vertical bars are 95% compatibility intervals. Orange=females, blue=males.

```
# Among-year SD in Ia
quantile(mod$sims.list$sigmaS1year, probs=c(0.025, 0.5, 0.975))
##       2.5%        50%      97.5% 
## 0.06308324 0.79646176 1.60066456

# Among-year SD in Ib
quantile(mod$sims.list$sigmaS2year, probs=c(0.025, 0.5, 0.975))
##     2.5%      50%    97.5% 
## 0.254814 0.760402 1.473444
```

```
# mean age equals -1/ln(S) if S is constant with age (exponential distribution)
# that is not the case -> Monte Carlo simulation to estimate mean age

nsim <- mod$n.sims
virtpop <- array(dim=c(nsim, 100, 2,2)) # max 100 years
virtpop[,1,,] <- 1 # all individuals are once born
for(i in 2:100){
  if(i==2){ 
    virtpop[,i,1,1] <- rbinom(nsim, size=virtpop[,i-1,1,1], prob=plogis(mod$sims.list$b10[,1,1]))
    virtpop[,i,2,1] <- rbinom(nsim, size=virtpop[,i-1,2,1], prob=plogis(mod$sims.list$b10[,2,1]))
        virtpop[,i,1,2] <- rbinom(nsim, size=virtpop[,i-1,1,2], prob=plogis(mod$sims.list$b20[,1,1]))
    virtpop[,i,2,2] <- rbinom(nsim, size=virtpop[,i-1,2,2], prob=plogis(mod$sims.list$b20[,2,1]))
  }
  if(i==3){ 
    virtpop[,i,1,1] <- rbinom(nsim, size=virtpop[,i-1,1,1], prob=plogis(mod$sims.list$b10[,1,2]))
    virtpop[,i,2,1] <- rbinom(nsim, size=virtpop[,i-1,2,1], prob=plogis(mod$sims.list$b10[,2,2]))
        virtpop[,i,1,2] <- rbinom(nsim, size=virtpop[,i-1,1,2], prob=plogis(mod$sims.list$b20[,1,2]))
    virtpop[,i,2,2] <- rbinom(nsim, size=virtpop[,i-1,2,2], prob=plogis(mod$sims.list$b20[,2,2]))
  }
  if(i>3){ 
   virtpop[,i,1,1] <- rbinom(nsim, size=virtpop[,i-1,1,1], prob=plogis(mod$sims.list$b10[,1,3]))
    virtpop[,i,2,1] <- rbinom(nsim, size=virtpop[,i-1,2,1], prob=plogis(mod$sims.list$b10[,2,3]))
        virtpop[,i,1,2] <- rbinom(nsim, size=virtpop[,i-1,1,2], prob=plogis(mod$sims.list$b20[,1,3]))
    virtpop[,i,2,2] <- rbinom(nsim, size=virtpop[,i-1,2,2], prob=plogis(mod$sims.list$b20[,2,3]))
  }
}

SurvF1 <- apply(virtpop[,,1,1], 2, sum)/nsim
SurvM1 <- apply(virtpop[,,2,1], 2, sum)/nsim
SurvF2 <- apply(virtpop[,,1,2], 2, sum)/nsim
SurvM2 <- apply(virtpop[,,2,2], 2, sum)/nsim

plot(0:99, SurvF1, type="l", lwd=2, col="orange", xlim=c(0,12), las=1, ylab="Proportion survived", xlab="Age [years]")
lines(0:99, SurvM1, lwd=2, col="blue")
lines(0:99, SurvF2, lwd=2, col="orange", lty=3)
lines(0:99, SurvM2, lwd=2, col="blue", lty=3)
legend(5,1, lwd=2, lty=c(1,1,3,3), col=c("orange", "blue", "orange", "blue"),
       legend=c("female I a", "males I a", "female I b", "male I b"))
```

Figure 2.3: Survivor curve for the two areas per sex

```
# average age
agefun <- function(x) max(c(1:100)[x==1])-0.5
mean(apply(virtpop[,,1,1], 1, agefun)) # females Ia
```

```
## [1] 4.6446
```

```
mean(apply(virtpop[,,2,1], 1, agefun)) # males Ia
```

```
## [1] 1.8332
```

```
mean(apply(virtpop[,,1,2], 1, agefun)) # females Ib
```

```
## [1] 2.208733
```

```
mean(apply(virtpop[,,2,2], 1, agefun)) # males Ib
```

```
## [1] 1.080867
```

```
# dimensions:
# 1: females, males
# 2: age classes
# 3: no telemtry vs. telemetry
tab <- data.frame(sex=c("females", "females", "females", "males", "males", "males", "telemetry"),
                  age=c(1,2,3,1,2,3,1))

tab$p_Ia <- plogis(c(apply(mod$sims.list$a10[,1,,1], 2, mean),
                      apply(mod$sims.list$a10[,2,,1], 2, mean), mean(mod$sims.list$a10[,1,1,2])))

tab$p_Ia.lwr <- plogis(c(apply(mod$sims.list$a10[,1,,1], 2, quantile, probs=0.025),
                      apply(mod$sims.list$a10[,2,,1], 2, quantile, probs=0.025), quantile(mod$sims.list$a10[,1,1,2], probs=0.025)))
tab$p_Ia.upr <- plogis(c(apply(mod$sims.list$a10[,1,,1], 2, quantile, probs=0.975),
                      apply(mod$sims.list$a10[,2,,1], 2, quantile, probs=0.975), quantile(mod$sims.list$a10[,1,1,2], probs=0.975)))


tab$p_Ib <- plogis(c(apply(mod$sims.list$a20[,1,,1], 2, mean),
                      apply(mod$sims.list$a20[,2,,1], 2, mean), mean(mod$sims.list$a20[,1,1,2])))

tab$p_Ib.lwr <- plogis(c(apply(mod$sims.list$a20[,1,,1], 2, quantile, probs=0.025),
                      apply(mod$sims.list$a20[,2,,1], 2, quantile, probs=0.025), quantile(mod$sims.list$a20[,1,1,2], probs=0.025)))
tab$p_Ib.upr <- plogis(c(apply(mod$sims.list$a20[,1,,1], 2, quantile, probs=0.975),
                      apply(mod$sims.list$a20[,2,,1], 2, quantile, probs=0.975), quantile(mod$sims.list$a20[,1,1,2], probs=0.975)))

kable(tab, dig=2, caption="Probabilities to get pictured within one 2-month period for the different sexes and age classes and if tagged by telemetry in the two areas.")
```

Table 2.3: Probabilities to get pictured within one 2-month period for the different sexes and age classes and if tagged by telemetry in the two areas.

| sex | age | p\_Ia | p\_Ia.lwr | p\_Ia.upr | p\_Ib | p\_Ib.lwr | p\_Ib.upr |
| --- | --- | --- | --- | --- | --- | --- | --- |
| females | 1 | 0.43 | 0.22 | 0.69 | 0.22 | 0.11 | 0.37 |
| females | 2 | 0.13 | 0.06 | 0.25 | 0.14 | 0.08 | 0.24 |
| females | 3 | 0.10 | 0.07 | 0.16 | 0.19 | 0.12 | 0.28 |
| males | 1 | 0.38 | 0.19 | 0.62 | 0.42 | 0.23 | 0.66 |
| males | 2 | 0.09 | 0.04 | 0.20 | 0.51 | 0.28 | 0.76 |
| males | 3 | 0.16 | 0.11 | 0.24 | 0.39 | 0.29 | 0.50 |
| telemetry | 1 | 0.94 | 0.90 | 0.97 | 0.94 | 0.90 | 0.97 |

```
quantile(mod$sims.list$a1, probs=c(0.5, 0.025, 0.975))
```

```
##       50%      2.5%     97.5% 
## 0.1726184 0.1052641 0.2386657
```

```
quantile(mod$sims.list$a2, probs=c(0.5, 0.025, 0.975))
```

```
##       50%      2.5%     97.5% 
## 0.5781956 0.3010586 0.8535705
```

```
tab <- data.frame(sex=c("females", "females", "females", "males", "males", "males"),
                  age=c(1,2,3,1,2,3))

tab$r <- plogis(c(apply(mod$sims.list$d0[,1,], 2, mean),
                      apply(mod$sims.list$d0[,2,], 2, mean)))
tab$r.lwr <- plogis(c(apply(mod$sims.list$d0[,1,], 2, quantile, prob=0.025),
                      apply(mod$sims.list$d0[,2,], 2, quantile, prob=0.025)))
tab$r.upr <- plogis(c(apply(mod$sims.list$d0[,1,], 2, quantile, prob=0.975),
                      apply(mod$sims.list$d0[,2,], 2, quantile, prob=0.975)))

kable(tab, dig=2, caption="Probability that a dead lynx is found from the model fitted to the combined data including age at death data.")
```

Table 2.4: Probability that a dead lynx is found from the model fitted to the combined data including age at death data.

| sex | age | r | r.lwr | r.upr |
| --- | --- | --- | --- | --- |
| females | 1 | 0.25 | 0.07 | 0.72 |
| females | 2 | 0.28 | 0.09 | 0.69 |
| females | 3 | 0.18 | 0.10 | 0.29 |
| males | 1 | 0.06 | 0.02 | 0.16 |
| males | 2 | 0.44 | 0.14 | 0.87 |
| males | 3 | 0.22 | 0.12 | 0.37 |

```
tab <- data.frame(sex=c("female", "female", "female", "male", "male","male"),
                  age=c(1,2,3,1,2,3))
tab$m12 <- c(apply(mod$sims.list$m012[,1,], 2, mean), apply(mod$sims.list$m012[,2,], 2, mean))
tab$m12.lwr <- c(apply(mod$sims.list$m012[,1,], 2, quantile, probs=0.025), apply(mod$sims.list$m012[,2,], 2, quantile, probs=0.025))
tab$m12.upr <- c(apply(mod$sims.list$m012[,1,], 2, quantile, probs=0.975), apply(mod$sims.list$m012[,2,], 2, quantile, probs=0.975))

tab$m21 <- c(apply(mod$sims.list$m021[,1,], 2, mean), apply(mod$sims.list$m021[,2,], 2, mean))
tab$m21.lwr <- c(apply(mod$sims.list$m021[,1,], 2, quantile, probs=0.025), apply(mod$sims.list$m021[,2,], 2, quantile, probs=0.025))
tab$m21.upr <- c(apply(mod$sims.list$m012[,1,], 2, quantile, probs=0.975), apply(mod$sims.list$m021[,2,], 2, quantile, probs=0.975))

tab$m13 <- c(apply(mod$sims.list$m013[,1,], 2, mean), apply(mod$sims.list$m013[,2,], 2, mean))
tab$m13.lwr <- c(apply(mod$sims.list$m013[,1,], 2, quantile, probs=0.025), apply(mod$sims.list$m013[,2,], 2, quantile, probs=0.025))
tab$m13.upr <- c(apply(mod$sims.list$m013[,1,], 2, quantile, probs=0.975), apply(mod$sims.list$m013[,2,], 2, quantile, probs=0.975))

tab$m23 <- c(apply(mod$sims.list$m023[,1,], 2, mean), apply(mod$sims.list$m023[,2,], 2, mean))
tab$m23.lwr <- c(apply(mod$sims.list$m023[,1,], 2, quantile, probs=0.025), apply(mod$sims.list$m023[,2,], 2, quantile, probs=0.025))
tab$m13.upr <- c(apply(mod$sims.list$m023[,1,], 2, quantile, probs=0.975), apply(mod$sims.list$m023[,2,], 2, quantile, probs=0.975))

kable(tab, dig=2, caption="Probabilities to move between the areas 1 (Ia), 2 (Ib) and 3 (outside) for each age and sex class.")
```

Table 2.5: Probabilities to move between the areas 1 (Ia), 2 (Ib) and 3 (outside) for each age and sex class.


| sex | age | m12 | m12.lwr | m12.upr | m21 | m21.lwr | m21.upr | m13 | m13.lwr | m13.upr | m23 | m23.lwr |
| --- | --- | --- | --- | --- | --- | --- | --- | --- | --- | --- | --- | --- |
| female | 1 | 0.10 | 0.02 | 0.21 | 0.02 | 0.00 | 0.21 | 0.09 | 0.01 | 0.12 | 0.04 | 0.00 |
| female | 2 | 0.00 | 0.00 | 0.01 | 0.00 | 0.00 | 0.01 | 0.00 | 0.00 | 0.02 | 0.00 | 0.00 |
| female | 3 | 0.00 | 0.00 | 0.01 | 0.00 | 0.00 | 0.01 | 0.00 | 0.00 | 0.02 | 0.00 | 0.00 |
| male | 1 | 0.06 | 0.01 | 0.14 | 0.07 | 0.01 | 0.19 | 0.05 | 0.00 | 0.24 | 0.07 | 0.00 |
| male | 2 | 0.03 | 0.02 | 0.05 | 0.02 | 0.01 | 0.04 | 0.03 | 0.01 | 0.04 | 0.02 | 0.01 |
| male | 3 | 0.03 | 0.02 | 0.05 | 0.02 | 0.01 | 0.04 | 0.03 | 0.01 | 0.04 | 0.02 | 0.01 |

# 3 Sensitivity to including both L and R lynxes

When both L and R individuals are included, survival will be underestimated because if one individual dies there might two “individuals” in the data (an R and an L belonging to the same individual) disappear. However, if we reduce the data to one of L or R individuals, because individuals that die early may be overrepresented among those deleted from the data by such a selection. Therefore, we refitted the model to data that were not reduced. We found that survival estimates were slightly lower for juveniles whereas for the other age classes, survival estimates were similar when the model was fitted to the non-reduced data.

```
load("modelfits/modelfit_jura_combined_ageyearinklLR231204.rda") # m per sex and age

S1 <- plogis(apply(mod$sims.list$b10, c(2,3), mean))
S1lwr <- plogis(apply(mod$sims.list$b10, c(2,3), quantile, probs=0.025))
S1upr <- plogis(apply(mod$sims.list$b10, c(2,3), quantile, probs=0.975))
 
S2 <- plogis(apply(mod$sims.list$b20, c(2,3), mean))
S2lwr <- plogis(apply(mod$sims.list$b20, c(2,3), quantile, probs=0.025))
S2upr <- plogis(apply(mod$sims.list$b20, c(2,3), quantile, probs=0.975))

tab <- expand.grid(age=c("juveniles", "subadult", "adult"), 
                   sex=c("females", "males"))
tab$Ia_S <- as.numeric(t(S1))
tab$Ia_S.lwr <- as.numeric(t(S1lwr))
tab$Ia_S.upr <- as.numeric(t(S1upr))
tab$Ib_S <- as.numeric(t(S2))
tab$Ib_S.lwr <- as.numeric(t(S2lwr))
tab$Ib_S.upr <- as.numeric(t(S2upr))
kable(tab, dig=2, caption="Average annual survival estimates from the combined model with 95% uncertainty interval fitted to data including both L and R lynxes.")
```

Table 3.1: Average annual survival estimates from the combined model with 95% uncertainty interval fitted to data including both L and R lynxes.


| age | sex | Ia\_S | Ia\_S.lwr | Ia\_S.upr | Ib\_S | Ib\_S.lwr | Ib\_S.upr |
| --- | --- | --- | --- | --- | --- | --- | --- |
| juveniles | females | 0.44 | 0.22 | 0.72 | 0.39 | 0.19 | 0.65 |
| subadult | females | 0.91 | 0.76 | 0.98 | 0.67 | 0.45 | 0.85 |
| adult | females | 0.87 | 0.80 | 0.92 | 0.70 | 0.59 | 0.79 |
| juveniles | males | 0.21 | 0.09 | 0.46 | 0.18 | 0.07 | 0.44 |
| subadult | males | 0.79 | 0.55 | 0.93 | 0.51 | 0.28 | 0.75 |
| adult | males | 0.79 | 0.70 | 0.87 | 0.68 | 0.57 | 0.78 |
